# A Novel Lactobacilli-Based Teat Disinfectant for Improving Bacterial Communities in the Milks of Cow Teats with Subclinical Mastitis

**DOI:** 10.3389/fmicb.2017.01782

**Published:** 2017-09-26

**Authors:** Jie Yu, Yan Ren, XiaoXia Xi, Weiqiang Huang, Heping Zhang

**Affiliations:** Key Laboratory of Dairy Biotechnology and Engineering, Ministry of Education, Key Laboratory of Dairy Products Processing, Ministry of Agriculture, Inner Mongolia Agricultural University, Hohhot, China

**Keywords:** subclinical mastitis, lactic acid bacteria, teat disinfectant, SMRT sequencing, bacterial community

## Abstract

Teat disinfection pre- and post-milking is important for the overall health and hygiene of dairy cows. The objective of this study was to evaluate the efficacy of a novel probiotic lactobacilli-based teat disinfectant based on changes in somatic cell count (SCC) and profiling of the bacterial community. A total of 69 raw milk samples were obtained from eleven Holstein-Friesian dairy cows over 12 days of teat dipping in China. Single molecule, real-time sequencing technology (SMRT) was employed to profile changes in the bacterial community during the cleaning protocol and to compare the efficacy of probiotic lactic acid bacteria (LAB) and commercial teat disinfectants. The SCC gradually decreased following the cleaning protocol and the SCC of the LAB group was slightly lower than that of the commercial disinfectant (CD) group. Our SMRT sequencing results indicate that raw milk from both the LAB and CD groups contained diverse microbial populations that changed over the course of the cleaning protocol. The relative abundances of some species were significantly changed during the cleaning process, which may explain the observed bacterial community differences. Collectively, these results suggest that the LAB disinfectant could reduce mastitis-associated bacteria and improve the microbial environment of the cow teat. It could be used as an alternative to chemical pre- and post-milking teat disinfectants to maintain healthy teats and udders. In addition, the Pacific Biosciences SMRT sequencing with the full-length 16S ribosomal RNA gene was shown to be a powerful tool for monitoring changes in the bacterial population during the cleaning protocol.

## Introduction

Bovine mastitis, an inflammation of the mammary gland, causes physical, chemical, and usually, bacteriological changes in milk and pathological changes in the glandular tissues of the udder that affect the quality and quantity of milk (Sharma and Jeong, 2013). It has a high incidence and prevalence in dairy cows, affecting the net earnings of milk producers worldwide (Frola et al., 2011). Generally, clinical mastitis is easily diagnosed by visible clinical manifestations, such as red, hot, and swollen mammary glands (Sharma et al., 2007). Subclinical mastitis has no visible clinical symptoms in mammary glands and in milk, but milk production decreases, somatic cell count (SCC) increases, pathogens are present in the secretion, and the milk composition is altered (Lafarge et al., 2004). Therefore, development of effective, reliable, and safe mastitis prevention and treatment strategies has become a focus of research.

In China, a teat disinfectant is usually applied to prevent mastitis in pasture. Teat disinfection pre- and post-milking is important not only to reduce the possibility of mastitis, but also to reduce the risk of bacterial contamination of milk (Suriyasathaporn and Chupia, 2011; Zucali et al., 2011). Currently, various teat cleaning disinfectants, including iodophor solution, iodine based gel, sodium hypochlorite, dodecyl benzene sulfonic acid, chlorine, chlorhexidine, phenolic compounds, alcohol, and guava leaf extract, have been used for pre-milking teat dipping (Ingawa et al., 1992; Foret et al., 2005; Gibson et al., 2008). These chemical disinfectants can reduce major pathogen infections; however, the high concentration of chemical substances has raised the concern of potential residues in milk (Galton et al., 1986). As a result, a natural substance that exhibits inhibitory activity against bacteria was developed as a teat disinfectant to minimize bacterial contamination in raw milk. A previous study showed that methanol extracts from guava leaves used as a pre-milking teat disinfectant could significantly reduce teat-end bacterial loads compared with routine udder sanitization without teat dipping (Kummee et al., 2015).

The application of probiotic lactic acid bacteria (LAB) is now considered the best choice for the treatment of many infectious human diseases and for the control of bovine mastitis (Tagg and Dierksen, 2003; Soleimani et al., 2010). It is well known that these bacteria are safe, non-pathogenic, and exhibit many properties that prevent spoilage and the growth of pathogenic bacteria (Mojgani and Ashtiani, 2006; Yezli et al., 2015). The *Lactobacillus* (*L.*) *plantarum* strains IMAU 80065 and IMAU 10155 were isolated from pickles and fermented camel milk from Sichuan and Inner Mongolia, China, respectively (Yu et al., 2012). They were screened from 347 isolates and produce bacteriocins that significantly inhibit the growth of *Escherichia* (*E.*) *coli*, *Staphylococcus* (*S.*) *aureus*, *Salmonella*, *Shigella*, and *Listeria* (Yu et al., 2015). The objective of this study was to compare the effectiveness of a novel teat disinfectant containing two probiotic *L. plantarum* strains with a commercial disinfectant (CD). We analyzed the SCC, the number of pathogenic bacteria, and the bacterial community of the raw milk. Furthermore, we used Pacific Biosciences (PacBio) single molecule, real-time sequencing technology (SMRT) based on the full-length 16S ribosomal RNA (rRNA) gene to accurately and systematically depict the bacterial profiles of cow milk.

## Materials and Methods

### Ethics Statement

The study protocol was approved by the Ethical Committee of Inner Mongolia Agricultural University (Hohhot, China) and was permitted by the owners of the sampled dairy farm. Every effort was made to minimize animal suffering.

### Treatment and Sample Collection

This study was conducted with 25 lactating Holstein-Friesian dairy cows (2- to 4-years-old) on the Mengele dairy farms of Inner Mongolia, China in May, 2016 when the cows were kept indoors at all times. All cows were fed a total mixed ration (hay 5 kg/d, corn silage 25 kg/d, mixed concentrate 10 kg/d, and sodium bicarbonate 0.15 kg/d) according to standard practice and were kept in a tied housing system. The average milk yield of each dairy cow was 30 kg. Milk samples were collected during the morning milking by members of the research team. At the beginning of the experiment, primary udder hygiene was performed by washing with sterile water and wiping with disposable cloths. Following surface cleaning, several streams of foremilk were then removed prior to sample collection. An approximately 100-mL milk sample was collected in a sterile bottle for determination of SCC. The SCC of raw milk was measured using a laser beam Bentley FTS/FCM400 Combi Instrument (Chaska, MN, United States). A threshold of 200,000 cells/mL is indicative of subclinical mastitis (Dervishi et al., 2016). Eleven cows used in this study were diagnosed with subclinical mastitis because the SCC in a majority of milk samples was ≥200,000 cells/mL. Sterile gloves were used throughout the sampling procedures.

The left and right two teats were treated separately as individual groups. Two teat treatments were applied to the two groups within each sampling phase. After the continued application of the primary hygiene protocol, the left two teats were immersed (dipping treatment) before and after milking with LAB disinfectant containing 5 × 10^10^ colony-forming units/mL LAB (LAB group). The control (CD) group (right) was treated with the CD (Dipal Concentrate 1+4, Delaval, Tianjin). A total of 50 mL of foremilk and milk was aseptically collected from a quarter and then the samples from the two quarters in the same group were mixed for further study. Cows were individually sampled before treatment (day 0), and after 1, 6, and 10 days of continued cleaning. After 10 days, five cows from each group were randomly selected to continue washing with sterile water, instead of the LAB and CD, and milk was collected 2 days later. A total of 76 raw milk samples were obtained from 11 cows. These samples were kept on ice and transferred to our laboratory. Seven samples may have been contaminated with fecal matter and were removed from our analysis. Detailed information about the samples and the corresponding cows is provided in **Table 1**. All analyses were performed in duplicate.

**Table 1:** Sample information, sequence abundance, and microbial diversity in cow milk samples.

No. of	Group	Sample	No. of	No. of	Shannon	Observed	Simpson	Chao1	Group	Sample	No. of	No. of	Shannon	Observed	Simpson	Chao1
cows			reads	OTU	index	species	index	index			reads	OTU	index	species	index	index
1	LAB-0d	A1	6555	1192	7.16	922.87	0.96	3392.02	CD-0d	A13	3688	1249	7.62	817.38	0.97	4483.40
2	(left)	A2	6679	3455	9.07	2042.06	0.97	16772.17	(right)	A14	4429	2572	8.76	1466.13	0.97	15423.85
3		A3	9897	2394	7.86	1640.09	0.97	8129.27		A15	13688	1333	6.93	1240.50	0.93	3431.12
4		A4	4300	2006	8.34	1208.03	0.97	8639.71		A16	10819	3001	8.51	2103.43	0.97	9601.19
6		A6	5687	751	6.89	667.01	0.96	1924.02		A18	9117	2189	8.03	771.51	0.96	6578.60
7		A7	/	/	/	/	/	/		A19	1527	342	6.25	1590.02	0.95	898.14
8		A8	5900	3150	9.23	1877.79	0.98	13951.11		A20	4812	2840	9.10	1592.44	0.98	16843.35
9		A9	6562	2961	8.93	1852.25	0.97	11596.76		A21	7635	3223	8.99	2050.98	0.98	12276.22
10		A10	/	/	/	/	/	/		A22	23183	11166	10.47	6572.17	0.99	56476.12
11		A11	5153	2052	8.30	1287.97	0.97	8157.45		A23	3287	1241	6.58	768.63	0.97	4980.30
12		A12	2475	1264	7.74	736.94	0.96	5977.67		A24	3618	550	7.57	476.21	0.95	1276.42

1	LAB-1d	B1	3755	1375	7.95	888.61	0.97	4824.68	CD-1d	B13	3292	2028	8.64	1118.53	0.98	13259.88
2	(left)	B2	5103	3188	9.24	1771.98	0.98	20551.50	(right)	B14	5671	3082	8.87	1789.23	0.96	15899.95
3		B3	6897	1609	7.50	1138.65	0.96	5058.56		B15	13711	2669	7.78	1891.32	0.97	8687.10
4		B4	2233	1242	7.87	718.42	0.97	6717.56		B16	3143	1615	8.26	965.31	0.97	7631.66
6		B6	14451	4104	8.73	452.17	0.97	14938.56		B18	3571	774	6.89	1329.25	0.97	2441.73
7		B7	12107	2347	7.97	2733.39	0.96	6779.81		B19	5017	1810	8.24	554.12	0.97	6122.95
8		B8	4789	2987	9.24	1789.25	0.98	18762.48		B20	3246	1504	7.83	904.15	0.96	6639.33
9		B9	6707	3074	9.02	1668.87	0.97	11995.95		B21	8187	3518	9.19	2247.88	0.98	12890.05
10		B10	8119	2065	7.78	1411.75	0.96	6915.19		B22	4892	2882	9.12	1632.40	0.98	16725.36
11		B11	9941	3385	8.87	2228.56	0.97	12402.71		B23	5948	1610	7.79	1119.12	0.97	5256.41
12		B12	8554	1719	7.62	1304.17	0.96	4996.50		B24	3012	1901	8.59	1039.87	0.98	12130.92

1	LAB-10d	C1	3178	892	7.38	645.95	0.97	2674.22	CD-10d	C13	/	/	/	/	/	/
2	(left)	C2	3145	1648	7.99	956.29	0.96	8580.05	(right)	C14	4063	1499	7.64	968.00	0.95	5391.37
3		C3	7374	2281	8.11	1489.71	0.97	8464.74		C15	7026	1839	7.63	1238.87	0.96	6279.81
4		C4	11108	4619	9.37	2913.94	0.98	18011.44		C16	3192	1686	8.25	981.91	0.97	8149.31
6		C6	3606	737	7.30	628.65	0.96	1832.79		C18	3463	689	6.94	554.82	0.96	1833.39
7		C7	9954	2079	7.98	1581.46	0.96	6002.89		C19	/	/	/	/	/	/
8		C8	3277	1793	8.15	1008.29	0.97	10711.01		C20	3424	1747	8.16	1036.35	0.97	8433.68
9		C9	5159	2806	9.07	1649.52	0.98	14030.57		C21	4758	1489	7.61	989.95	0.96	5243.47
10		C10	3852	2105	8.36	1189.64	0.97	12922.15		C22	3297	1844	8.34	1035.93	0.97	10845.60
11		C11	3749	1887	8.40	1114.21	0.98	9056.02		C23	3651	1723	8.32	1042.89	0.98	7694.28
12		C12	/	/	/	/	/	/		C24	/	/	/	/	/	/

1	LAB-12d	D1	15246	1597	7.56	1634.83	0.96	5325.72	CD-12d	D13	/	/	/	/	/	/
2	(left)	D2	3681	1956	8.33	1119.76	0.97	10771.09	(right)	D14	6146	3760	9.52	2140.72	0.98	22654.27
6		D6	3172	424	6.58	414.00	0.96	1026.22		D18	7358	1424	7.41	1075.02	0.96	4210.00
8		D8	8431	2463	8.05	1645.65	0.96	8424.55		D20	9590	2608	8.35	1816.12	0.97	8499.17
10		D10	6995	3206	8.62	1872.78	0.97	16433.13		D22	8338	2134	7.97	1494.37	0.97	6702.55


### DNA Extraction and Quantitative Polymerase Chain Reaction (qPCR)

A 3-mL milk sample was centrifuged at 7000 × *g* for 20 min in an Eppendorf 5810R centrifuge and the supernatant was removed. The remaining pellet was resuspended in 500 mL of sterile water and 500 mg of lysozyme were added prior to incubation for 12 h at 37°C to maximize bacterial DNA extraction. Genomic DNA was extracted using the DNeasy mericon Food Kit (69514, Qiagen, Germany) according to the manufacturer’s instructions. Finally, 100 μL of elution buffer were added, and elution was performed following a 20-min incubation at room temperature. DNA concentration and purity were evaluated using 1.0% agarose gel electrophoresis and optical density using a NanoDrop ND-1000 spectrophotometer (Thermo Fisher Scientific, Wilmington, DE, United States). All extracted DNA samples were stored at -20°C until further use.

To accurately quantify pathogenic bacteria and lactobacilli in raw milk, *E*. *coli*, *S*. *aureus*, *Streptococcus* (*S*.) *agalactiae*, and *Lactobacillus* were chosen as targets for qPCR analysis with primers designed with the Primer Premier 5.0 program (Premier Biosoft International, Palo Alto, CA, United States) (Supplementary Table S1). qPCR was performed using a one-step real-time PCR System (Applied Biosystems, Foster City, CA, United States) and a SYBR Premix Ex Taq II kit (Takara Bio Inc., Japan). The amplification reaction mixture and program were prepared as previously described (Ma et al., 2015). Corresponding strains were used for standard curve construction, the coefficient of determination (R^2^ value) of the q-PCR was greater than 0.990, and the efficiency value ranged from 96.3 to 101.8%. PAST software was used to perform statistical analyses (Hammer and Harper, 2009). Data generated by qPCR were expressed as log gene copy number per mL of sample. Bacterial amounts were expressed as means ± standard error and presented as box-plots. The Mann–Whitney test with Bonferroni correction for multiple testing was used to evaluate the difference between samples in a pairwise manner. Sample groups with corrected *p*-values < 0.05 were considered significantly different.

### PCR Amplification and SMRT Sequencing

Genomic DNA was used as a template for PCR amplification of the 16S rRNA gene with primers 27F (*5′-GAGAGTTTGATCCTGGCTCAG-3′*) and 1541R (*5′-AAGGAGGTGATCCAGCCGCA-3′*), which contained a set of 16-nucleotide barcodes for barcoded SMRT sequencing of the full-length 16S rRNA gene. The PCR conditions were as follows: 95°C for 4 min and 30 cycles at 95°C for 1 min, 60°C for 45 s, and 72°C for 1 min, followed by a final cycle at 72°C for 7 min (2720 Thermal Cycler, Applied Biosystems) (Liu et al., 2015). Amplicons were sequenced using P6-C4 chemistry on a PacBio RS II instrument (Pacific Biosciences, Menlo Park, CA, United States). Verification of the amplicons and sequence preprocessing were performed as previously described (Mosher et al., 2013).

### Data Analysis

Raw sequence data were processed using the RS_ReadsOfinsert.1 protocol available in the SMRT Portal (version 2.7) (Hou et al., 2015). The extracted high-quality sequences were analyzed using the Quantitative Insights into Microbial Ecology (QIIME) package (version 1.7) and alignment of high-quality sequences under 100% clustering of sequence identity was performed using PyNAST and UCLUST softwares (Caporaso et al., 2009; Edgar, 2010). The unique sequence set was classified into operational taxonomic units (OTUs) under a threshold of 98.6% identity using UCLUST after selection of the representative sequences (Lozupone et al., 2006). Each OTU representative sequence was identified using the ribosomal database project (RDP) II database with a minimum bootstrap threshold of 80% (Cole et al., 2007). The *de novo* taxonomic tree was constructed based on the representative OTU set in FastTree software (Price et al., 2009) for downstream analysis, including the beta diversity calculation. The Shannon–Wiener, Simpson’s diversity, Chao1, and rarefaction estimators were calculated to evaluate alpha diversity. UniFrac distance was calculated based on the phylogenetic tree (Lozupone and Knight, 2005). Both weighted and unweighted calculations were performed for principal coordinate analysis (PCoA). Graphs were generated with the R package (version 3.1.2) and Origin software (version 8.5).

## Results

### SCC Determination

**FIGURE 1:**
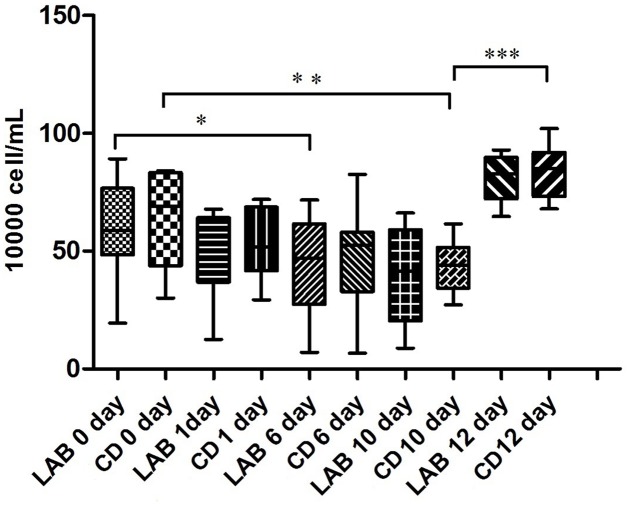
Changes in SCC in cow milk during the cleaning process with LAB or chemical disinfectant (CD). Significant differences between sample pairs were evaluated by pairwise Mann–Whitney test with Bonferroni correction; ^∗^*p* < 0.05, ^∗∗^*p* < 0.01, and ^∗∗∗^*p* < 0.001.

The SCC of 69 milk samples ranged from 6.7 to 102 × 10^4^ cells/mL during the cleaning protocol (**Figure 1**). The SCC gradually decreased over the course of the experiment and the SCC of the LAB group was slightly lower than that of the CD group. However, the SCC increased significantly after dipping with sterile water suggesting that LAB and CDs may help prevent mastitis.

### Quantitative PCR of the *Lactobacillus* Genus and Pathogenic Bacteria

**FIGURE 2:**
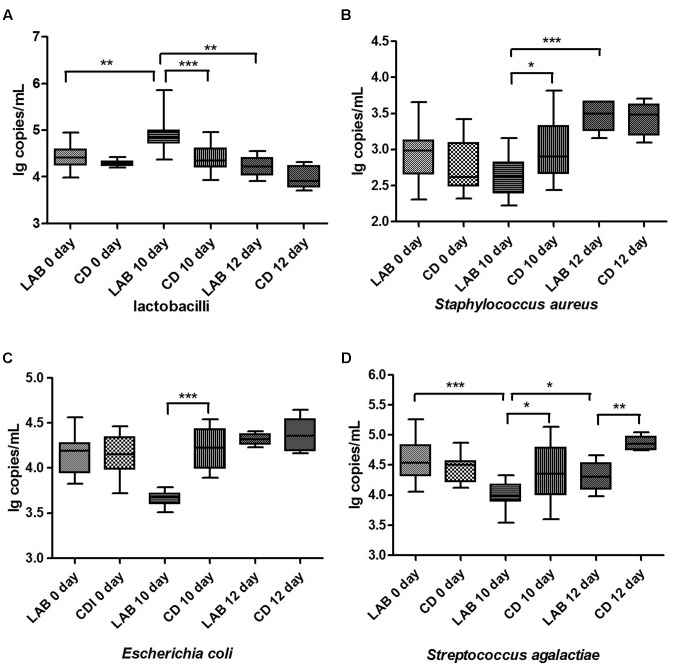
Quantification of *Lactobacillus* **(A)**, *Staphylococcus aureus* **(B)**, *Escherichia coli* **(C)** and *Streptococcus agalactiae* **(D)** in cow milk. Bacterial amounts are expressed in log copy number per milliliter of milk sample. Boxes show the median and 25th and 75th percentiles; the lower and upper adjacent hinges show the minimum and maximum values. Significant differences between sample pairs were evaluated by pairwise Mann–Whitney test with Bonferroni correction; ^∗^*p* < 0.05, ^∗∗^*p* < 0.01, and ^∗∗∗^*p* < 0.001.

The *Lactobacillus* genus and common pathogenic bacteria in raw milk, including *E*. *coli*, *S*. *aureus*, and *S. agalactiae*, were quantified by qPCR, and the bacterial composition over time is presented in **Figure 2** and Supplementary Table S2. The mean amounts of *E*. *coli*, *S*. *aureus*, and *S. agalactiae* in the LAB group after 10 days of the cleaning protocol were significantly lower than the other groups, while the *Lactobacillus* genus in the LAB group after 10 days was significantly higher than in the other groups (*p* < 0.05). In particular, after dipping with sterile water, the amount of *S. agalactiae* in the CD group was significantly higher than in the LAB group (*p* < 0.001). Our results show that both LAB and CDs can inhibit these pathogenic bacteria and the LAB disinfectant exhibits long-term effectiveness against *S. agalactiae.*

### Sequence Abundance and Diversity

**FIGURE 3:**
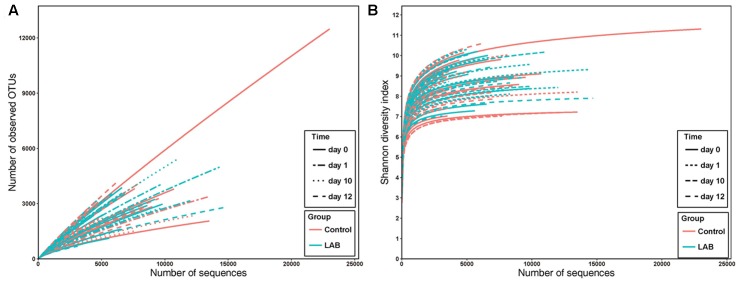
Rarefaction analysis **(A)** and Shannon diversity **(B)** estimates of the SMRT sequencing reads of bacteria in cow milk. Lines with colors represent samples from different groups. Lines with different textures represent samples from different times.

We performed SMRT sequencing of the full-length 16S rRNA gene to obtain accurate bacterial profiles of raw milk at the species level. A total of 503,162 raw reads were generated from 69 milk samples, with an average of 6,341 reads per sample. The total number of unique and classifiable representative bacterial OTU sequences was 15,274 (average = 2213.25 OTUs per sample, range = 342–11166, standard deviation = 913.65). The Shannon index, Simpson diversity index, Chao1, and observed species of each sample were used to evaluate species richness and diversity (**Table 1**). These values indicated that the majority of samples exhibited a high level of bacterial biodiversity. The Shannon diversity curves indicated that the sequence depth obtained was adequate for all samples (**Figure 3**).

### Change in Bacterial Composition Following Cleaning

Based on the homologous sequence alignment and clustering with the information extracted from the RDP and Greengenes (version 13_8) databases, we determined the lowest level of taxonomy of the identified OTUs. A total of 2.55% of the bacterial sequences could not be identified at the genus level (Supplementary Table S3). From the 69 samples, 25 phyla, 373 genera, and 796 species were identified. *Firmicutes* (91.14%) and *Proteobacteria* (7.96%) were the most prevalent phyla, which exhibited no significant differences in the LAB and CD groups during the cleaning process.

At the genus level, the major bacterial genera with relative abundances >1% belonged to *Bacillus* sp. (average 76.17%), *Pseudomonas* sp. (average 4.05%), *Lactococcus* sp. (average 3.55%), *Oceanobacillus* sp. (average 2.80%), and *Lactobacillus* sp. (average 1.49%) (Supplementary Table S3). Over the course of the experiment, the relative abundance of *Bacillus* sp. increased, although its relative abundance gradually decreased after dipping with sterile water. In the LAB and CD groups, *Bacillus* sp. showed no significant differences during the cleaning protocol. The relative abundance of *Pseudomonas* sp. was sharply decreased after cleaning with LAB or chemical disinfectant, but was significantly increased in the CD group after dipping with sterile water. The relative abundance of *Lactococcus* sp. was increased after cleaning with LAB, but decreased after cleaning with chemical disinfectant. This difference was significant in the two groups. After cleaning with sterile water, the amount of *Lactococcus* sp. in the LAB group decreased and was equal to that of the CD group. *Lactobacillus* sp. in the LAB group rapidly increased from 0 to 12 days, while there was almost no change in *Lactobacillus* sp. in the CD group.

**FIGURE 4:**
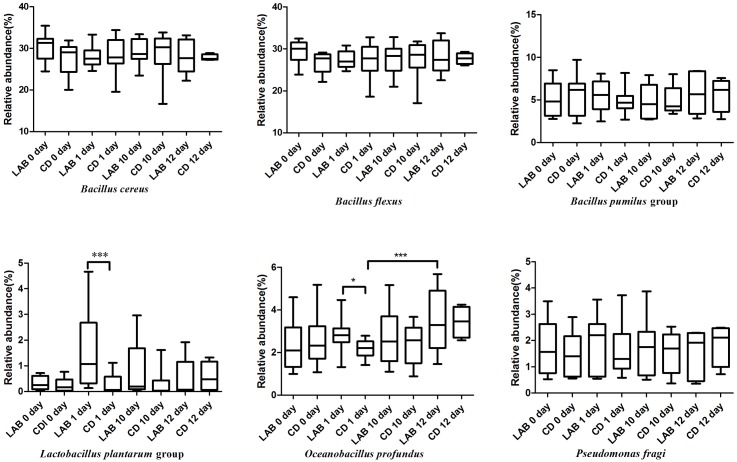
Boxplots of the relative abundance of six high abundance (>1%) bacteria detected in cow milk samples at the species level with significant differences.

In both the LAB and CD groups, the bacterial species primarily included *Bacillus* (*B.*) *cereus* (average 28.43%), *B. flexus* (average 27.94%), *Oceanobacillus* (*O.*) *profundus* (average 2.74%), the *B. pumilus* group (average 5.28%), *Lactococcus piscium* (average 2.74%), *Pseudomonas fragi* (average 1.67%), *Lactococcus lactis* (average 0.81%), and the *L. plantarum* group (average 0.96%) (Supplementary Table S3). As shown in **Figure 4**, the relative abundance of *B*. *flexus* increased during the cleaning protocol. *Bacillus cereus* increased slightly and then decreased after dipping with sterile water. The relative abundances of the *B*. *pumilus* group and *Pseudomonas stutzeri* were stable over the 12 days. The three species in the *Bacillus* genus exhibited no significant differences between the LAB and CD groups. The *L. plantarum* group increased sharply during the cleaning protocol with LAB, while there were no significant changes in the CD group. *Oceanobacillus profundus* increased and was significantly higher in the LAB group than in the CD group.

**FIGURE 5:**
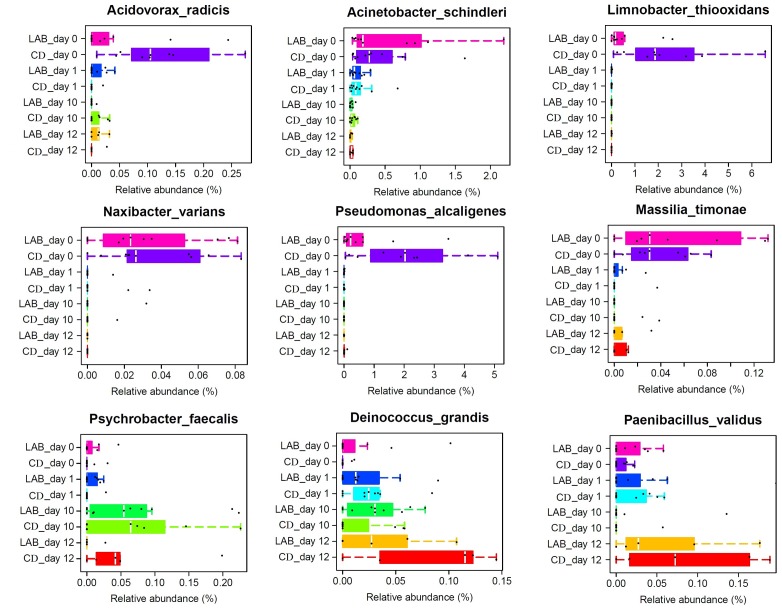
Boxplots of the relative abundance of nine low abundance (<1%) bacteria detected in cow milk samples at the species level with significant differences.

The relative abundance of some low abundance (<1%) species changed significantly during the cleaning protocol (**Figure 5**). These species included *Acinetobacter schindleri*, *Acidovorax radicis*, *Psychrobacter faecalis*, *Limnobacter thiooxidans*, *Massilia* (*M.*) *timonae*, *Naxibacter* (*N*.) *varians*, *Paenibacillus validus*, *Deinococcus* (*D.*) *grandis*, and *Pseudomonas alcaligenes*. *Acinetobacter schindleri*, *Acidovorax radicis*, *Limnobacter thiooxidans*, *N*. *varians*, and *Pseudomonas alcaligenes* were detected at 0 day and their relative abundances decreased to almost zero following cleaning. *Deinococcus grandis* increased during the cleaning protocol, while *M*. *timonae* decreased sharply at 6 days, but its relative abundance increased gradually after dipping with sterile water. The abundance of *Psychrobacter faecalis* initially increased then subsequently decreased.

### Comparison of the Bacterial Community Structure

**FIGURE 6:**
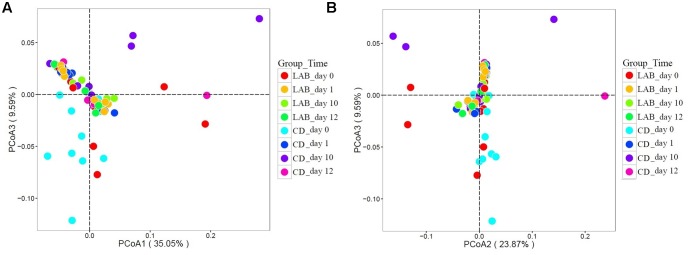
Weighted UniFrac principal coordinate analysis (PCoA) of the bacterial communities in cow milk samples. **(A)** PCoA scores plot based on weighted UniFrac principal components 1 and 3. **(B)** PCoA scores plot based on weighted UniFrac principal components 2 and 3. Each symbol represents the cow milk microbiota of one sample; sample group is represented by the respective color.

To compare the structure of the bacterial community in all groups, we performed weighted UniFrac PCoA based on the OTU abundance table. Principal coordinates based on the weighted (PC1 and PC3 accounted for 35.05 and 9.59% of the total variance, respectively) (**Figure 6A**) and (PC2 and PC3 accounted for 23.87 and 9.59% of the total variance, respectively) (**Figure 6B**) UniFrac distances revealed apparent bacterial structural differences, as the symbols representing samples of the CD groups at 0 and 10 days were separated on both PCoA score plots with only minor overlap. However, the symbols representing samples of the other five groups overlapped. Results from MANOVA based on unweighted (*p <* 0.05) UniFrac distances further confirmed the structural differences in bacterial composition between the two sample groups at 10 days.

## Discussion

Microorganisms in raw milk directly impact the flavor, quality, and shelf life of milk products. Moreover, the presence of pathogens in raw milk can lead to severe illness (Oliver et al., 2009; Quigley et al., 2013). The application of teat disinfection pre- and post-milking is recommended to reduce the number of bacteria on the teat skin and in milk (Oliver et al., 1993). The aims of this study were to harness the PacBio SMRT sequencing technology to investigate the influence that LAB teat disinfectant and CD have on teat milk microbiota from individual cows. Our findings confirm the efficacy of LAB disinfectant in inhibiting pathogenic bacterial growth and improving the bacterial populations of raw milk during the cleaning process.

Currently, the SCC and the California mastitis test (CMT) are used to diagnose mastitis. Somatic cells are naturally present in milk, which is widely used to distinguish healthy quarters from quarters with an inflammatory response most likely due to an intramammary infection (Schukken et al., 2003; Oikonomou et al., 2014). Usually, an SCC of <100,000 cells/mL is considered healthy, and a threshold of 200,000 cells/mL was shown to have high sensitivity and specificity as an identifier of subclinical mastitis (Hillerton, 1999; Diepers et al., 2016). In this study, cows were selected based on this criterion. During the course of teat disinfectant treatment, the SCC gradually decreased and the SCC of the LAB group was slightly lower than that of the CD group. Although the SCC did not decrease to a healthy level, changes were seen in the microfloral structure of teat milk. Previous research also showed that the microfloral structure of bulk tank milk is associated with the SCC (Rodrigues et al., 2017).

Generally, *S*. *aureus*, *S. uberis*, and *S. agalactiae* are recognized as major virulent mastitis-causing bacteria (Kuehn et al., 2013). Therefore, researchers have focused on developing a safe and effective alternative to the use of chemicals to inhibit these pathogenic bacteria. Previous studies demonstrated the inhibitory effects of secreted bacteriocins, like nisin and lacticin 3147 (Cao et al., 2007; Klostermann et al., 2010), and live probiotic microorganisms, in *in vitro* and *in vivo* approaches (Espeche et al., 2009; Diepers et al., 2016). Interestingly, Oikonomou et al. (2014) reported that these bacterial species were present in small quantities in healthy cow milk and hypothesized that they are part of the normal bacterial flora of the mammary gland. In our study, these bacterial species were also detected in lower quantities in the majority of milk samples by SMRT sequencing and qPCR. However, the mean amounts of *S*. *aureus* and *S. agalactiae* gradually decreased during the cleaning protocol, particularly in the LAB group, after 10 days (**Figure 2**). This result confirms that our LAB disinfectant possesses antibacterial activity primarily against mastitis-causing bacteria *in vivo*. Moreover, the antibacterial effect of LAB was marginally better than that of the chemical disinfectant.

Probiotic products against udder diseases have been developed over the past few decades and comprise bacteriocins for external application, intramammarily applied bacteriocins, and live probiotic microorganisms. Encouraging results were obtained with the injection of live cultures of selected LAB strains into the bovine udder, which were able to inhibit several mastitis pathogens (Crispie et al., 2008; Frola et al., 2011). Results have shown that preparations of the live culture were as effective as commonly used antibiotics for the treatment of intramammary infections and did not show adverse effects on mammary tissue. Moreover, the bovine teat surface can contain a high diversity of bacteria (Verdiermetz et al., 2012). Monsallier et al. (2012) proposed that the teat skin was a source of microbial populations in raw milk and that farm management and animal grazing practices influenced the diversity and microbiota of raw milk. Another study also highlights that the teat surface and herd habitats are significant drivers of milk microbiota composition (Doyle et al., 2017). Hence, it is beneficial to use a probiotic LAB teat disinfectant as a protective barrier to inhibit pathogens and improve the microbial balance of the teat. Our data provide new insights into probiotic LAB regulation of the bacterial composition of the teat and suggest a role for LAB in the prevention of mastitis. The results revealed differences in the bacterial composition of milk during the cleaning process with two disinfectants. These differences were reflected in the beta-diversity measurements, as illustrated in the PCoA analysis of the UniFrac distance (**Figure 6**). The UniFrac PCoA showed that the non-cleaned (0 day) and cleaned, LAB cleaned, and chemical cleaned samples mostly fell within separate clusters. That means the LAB teat disinfectant can alter the microbial structure of teat milk.

Analysis of the bacterial composition of the milk samples after 1, 6, 10, and 12 days of teat dipping in the same individuals revealed bacterial species diversity (**Figure 4** and Supplementary Figure S1). The relative abundance of the *L. plantarum* group in the LAB group following cleaning was slightly higher than in the CD group. This demonstrates that the LAB disinfectant could be transferred through the milk ducts into the teat and mammary glands to further regulate the bacterial community of the teat. In addition to its presence in raw milk, *O*. *profundus* has been found in deep-sea sediment core samples and wheat rhizospheric soil (Kim et al., 2007; Aislabie et al., 2008; Ranieri et al., 2012). The relatively high abundance of *O*. *profundus* in our samples may have been caused by potential contamination from the feeding environment and forage grass. It is worth noting that the relative abundance of *O*. *profundus* in the LAB group was significantly higher than in the CD group, suggesting that further research is needed to determine the role *O*. *profundus* may play in the microbiota of cow teats.

The relative abundances of *Acinetobacter schindleri*, *Acidovorax radicis*, *Limnobacter thiooxidans*, *N*. *varians*, *M. timonae*, *Paenibacillus validus*, and *Pseudomonas alcaligenes* gradually decreased following cleaning (**Figure 5**). With the exception of *Pseudomonas alcaligenes*, these species are not commonly found in milk. *Acinetobacter schindleri* was first found in the urine of a male outpatient with cystitis (Nemec et al., 2001) and the *Acinetobacter* genus is widely distributed in nature and frequently found in milk, soil, and water (Dortet et al., 2006; Li et al., 2016). *Acidovorax radicis* is a wheat root-colonizing bacterium that was previously identified from surface-sterilized wheat roots (Li et al., 2011). *Limnobacter thiooxidans* is a thiosulfate-oxidizing bacterium that was isolated from freshwater lake sediment (Spring et al., 2001). *Massilia timonae* was isolated from patient blood and soil (Lindquist et al., 2003) and *N*. *varians* was identified in clinical specimens and water (Kämpfer et al., 2008). *Pseudomonas alcaligenes* is a human pathogen, but occurrences are rare. Previous studies have shown that *Pseudomonas* spp. are responsible for the spoilage of milk and dairy products because of their ability to produce heat-resistant proteolytic and lipolytic enzymes at low temperatures (Ercolini et al., 2009; Zhang et al., 2015). The presence of these species may originate from components of the environment in contact with the teat skin. Our results suggest that teat dipping with LAB and CDs have identical efficacy in the reduction of these contaminating species. *Paenibacillus validus* was decreased at 10 days with both disinfectants, but increased sharply after cleaning with water for 2 days. This species is abundant in soil but rarely found in milk (Hildebrandt et al., 2006), while other *Paenibacillus* species have been detected in cow’s milk and various milk products. *Paenibacillus* spp. are of interest as they have been associated with the biocontrol of pathogenic bacteria and a decreased shelf life of milk products (Ziarno and Zareba, 2010; Debois et al., 2013). In particular, Ranieri et al. (2012) suggested that *Paenibacillus* spp. could represent an important tool for determining the quality of raw milk, as well as for identifying potential contamination sites at the farm and processing facility. Obviously, both LAB and CDs have the ability to decrease the relative abundance of *Paenibacillus validus*, although LAB disinfectants are more durable.

*Deinococcus grandis* and *Psychrobacter faecalis* increased during the cleaning protocol. *Deinococcus grandis* is a radioresistant bacterium that was initially isolated from freshwater fish and animal feces (Oyaizu et al., 1987). Some species in the *Deinococcus* genus are found in milk products. Members of the *Deinococcus* genus are also used as ingredients in food to ferment soymilk and as a feed additive for hens to enhance yolk coloration to meet customer demand (Wu et al., 2016). *Psychrobacter faecalis* was first isolated from pigeon feces and has not been detected in milk. While *Psychrobacter* spp. have been found at a relatively high level in the raw milk of healthy cows compared with cows with mastitis (Kuehn et al., 2013), these bacteria were not previously associated with mastitis (Vacheyrou et al., 2011). Given the change in the amounts of these species during the cleaning protocol, we favor the explanation that in small amounts they are part of the normal bacterial flora of the teat and may encourage teat microflora balance. Future research should aim to investigate the origin of these species and their role in teat microflora.

Three species of *Bacillus* were dominant in our samples. Previous studies have reported the presence of *Bacillus* spp. in raw milk (Hou et al., 2015) because of their heat resistance properties and intrinsic antagonism toward other microbes (Griffiths and Phillips, 1990). The season (summer, approximately 30°C) and sanitary conditions of the location of the sample collection may have resulted in the higher relative abundance of *Bacillus* spp. in the teat, which did not exhibit significant changes during the cleaning process. This suggests that these two different cleaning disinfectants were not able to inhibit *Bacillus* spp.; hence, it will be important to screen additional LAB strains and develop novel disinfectants aimed specifically at *Bacillus* spp.

In this study, we used the latest PacBio SMRT sequencing technology to describe the changes in the bacterial community during the cleaning protocol based on the full-length 16S rRNA gene. Compared with previous sequencing approaches, the PacBio SMRT technology has a high capacity for the production of long reads and has been shown to offer higher taxonomic resolution in the profiling of bacterial communities in dairy product samples to the species level (Hou et al., 2015; Li et al., 2016; Zheng et al., 2016). It was therefore not surprising to find more uncommon species and numerous unidentified sequences or sequences identified as uncultured bacteria in our study. Previous data on the bacterial profile of dairy products were generated largely by second-generation sequencing technology, which generates high quality, though short, sequence reads that preclude the accurate assignment of DNA sequences at the species level and even limits classification at the genus level (Amir et al., 2013). Furthermore, PacBio SMRT technology can greatly shorten the time required to detect and analyze the overall bacterial profile of a sample to 24 h (Zheng et al., 2016). By employing SMRT sequencing technology coupled with full-length 16S rRNA sequence determination in this study, clear bacterial population structural differences in the teat were revealed in 69 raw milk samples during the cleaning protocol using LAB or chemical disinfectant.

Rainard (2017) was skeptical about the potential of probiotics as a potential preventive solution for mastitis control. However, the present study demonstrates that teat dips containing LAB possess the ability to reduce both the SSC and pathogenic bacteria, thus regulating the bacterial composition of the teat. Moreover, the ability of LAB to increase beneficial microbes was relatively higher than chemical disinfectants. Overall, our findings indicate that LAB as a teat disinfectant is antimicrobial, non-irritating, and highly efficient and can be used for teat dipping following a routine udder sanitization procedure. Previous studies have been devoted to the development of novel disinfectants, such as plant extracts, to replace chemical disinfectants (Zeedan et al., 2014; Kummee et al., 2015).

This is the first report on the use of PacBio SMRT sequencing technology to evaluate the impacts of LAB disinfectant on the bacterial composition of the teat and comparison with a chemical disinfectant. Our results indicate that the teat bacterial community is highly diverse and complex. Both LAB and CD altered the bacterial composition of the teat and, in particular, reduced the number of mastitis-causing bacteria. Teat dipping with LAB has advantages over chemical disinfectant in that it is harmless to the milker’s health and non-toxic to milk consumers. Therefore, LAB disinfectant could be used as an alternative to chemical pre- and post-milking teat disinfectants to maintain udder health. Some species showed significant differences between cleaning and non-cleaning, suggesting these species could also be used as markers of udder health. In addition, the PacBio SMRT sequencing technology, a powerful and valuable tool, provided accurate microbiota profiling data in our study.

## Accession Number

Sequencing data for the 16S rRNA sequences have been deposited in the Metagenomics Rapid Annotation Server with the accession number MGP80281.

## Author Contributions

JY and HZ designed the experiments. JY, YR, XX, and WH performed the experiments. JY, YR, and HZ drafted the manuscript. All authors read and approved the final manuscript.

## Conflict of Interest Statement

The authors declare that the research was conducted in the absence of any commercial or financial relationships that could be construed as a potential conflict of interest.
