# Supplementary material for: A Novel Lactobacilli-Based Teat Disinfectant for Improving Bacterial Communities in the Milks of Cow Teats with Subclinical Mastitis
Source: Front Microbiol. 2017 Sep 26;8:1782. doi: 10.3389/fmicb.2017.01782 (PMC5622921; doi:10.3389/fmicb.2017.01782)
Supplement: Supplementary file 1 [file Table_1.DOCX]

Table S1. Quantitative PCR primer sequences and application condition used in this study

| Target microbial groups | Primers | DNA Sequence (5’- 3’) | Target gene | Predicted PCR product size (bp) | Annealing Temperature (oC) | References |
| --- | --- | --- | --- | --- | --- | --- |
| *Staphylococcus aureus* | Staphy-f | ACTGCTATCCACCCTCAAA | *mec*A gene | 279 | 60 | (1) |
|  | Staphy-r | TTTTGCCAACCTTTACCATC |  |  |  |  |
| *Escherichia coli* | Ecoli-f | CATGCCGCGTGTATGAAGAA | 16s rRNA gene | 428 | 60 | (2) |
|  | Ecoli-r | CGGGTAACGTCAATGAGCAAA |  |  |  |  |
| *Lactobacillus* | Lacto-f | AGCAGTAGGGAATCTTCCA | 16s-23s rRNA gene | 341 | 58 | (3) |
|  | Lacto-r | CACCGCTACACATGGAG |  |  |  |  |
| *Streptococcus agalactiae* | Strep-f | AGCTTAGTTATCCCAAATCCCAT | *cfb* gene | 154 | 56 | (4) |
|  | Strep-r | GTGCCAACCCTGAGACAGTT G |  |  |  |  |

Reference

1. Wada M, Lkhagvadorj E, Bian L, Wang C, Chiba Y, Nagata S, Shimizu T, Yamashiro Y, Asahara T, Nomoto K. 2010. Quantitative reverse transcription-PCR assay for the rapid detection of methicillin-resistant *Staphylococcus aureus*. J Appl Microbiol 108:779.

2. Furet JP, Firmesse O, Gourmelon M, Bridonneau C, Tap J, Mondot S, Doré J, Corthier G. 2009. Comparative assessment of human and farm animal faecal microbiota using real-time quantitative PCR. Fems Microbiol Ecol 68:351-362.

3. J Š, Sepp E, Kolk H, Lõivukene K, Songisepp E, Mikelsaar M. 2011. Diversity and metabolic impact of intestinal *Lactobacillus* species in healthy adults and the elderly. Brit J Nutr 105:1235.

4. Sebastião FDA, Lemos EGM, Pilarski F. 2015. Validation of absolute quantitative real-time PCR for the diagnosis of *Streptococcus agalactiae* in fish. J Microbiol Meth 119:168-175.
