# Supplementary material for: A Novel Lactobacilli-Based Teat Disinfectant for Improving Bacterial Communities in the Milks of Cow Teats with Subclinical Mastitis
Source: Front Microbiol. 2017 Sep 26;8:1782. doi: 10.3389/fmicb.2017.01782 (PMC5622921; doi:10.3389/fmicb.2017.01782)
Supplement: Supplementary file 2 [file Table_2.DOCX]

Table S2. Quantification of *Lactobacillus* genus, *Escherichia coli*, *Staphylococcus aureus* and *Streptococcus agalactiae* in the cow milk.

| Target microbial groups | No. of Samples | LAB 0d | CD 0d | LAB 10d | CD 0d | LAB 12d | CD 12d |
| --- | --- | --- | --- | --- | --- | --- | --- |
|  |  | Log gene copy number/mL of sample | | | | | |
| *Escherichia coli* | 1 | 4.42 | 3.87 | 3.63 | 4.54 | 4.33 | / |
|  | 2 | 3.90 | 4.46 | 3.67 | 4.45 | 4.29 | 4.36 |
|  | 3 | 3.82 | 4.38 | 3.67 | 4.38 | / | / |
|  | 4 | 3.93 | 4.16 | 3.71 | 4.28 | / | / |
|  | 6 | 4.57 | 4.15 | 3.67 | 3.99 | 4.32 | 4.64 |
|  | 7 | / | 4.23 | 3.72 | 4.05 | / | / |
|  | 8 | 4.18 | 4.02 | 3.72 | 4.20 | 4.23 | 4.44 |
|  | 9 | 4.13 | 3.97 | 3.51 | 4.49 | / | / |
|  | 10 | / | 4.38 | 3.55 | 3.92 | 4.41 | 4.17 |
|  | 11 | 4.20 | 4.10 | 3.79 | 3.89 | / | / |
|  | 12 | 4.20 | 3.72 | 3.60 | 4.26 | / | / |
|  | Means±SD | 4.15±0.24 | 4.13±0.22 | 3.66±0.08 | 4.22±0.22 | 4.32±0.31 | 4.40±0.50 |
| *Staphylococcus aureus* | 1 | 2.98 | 2.43 | 2.24 | 3.10 | 3.37 | / |
|  | 2 | 3.08 | 2.52 | 2.39 | 3.82 | 3.16 | 3.55 |
|  | 3 | 2.71 | 3.03 | 3.14 | 2.86 | / | / |
|  | 4 | 2.94 | 2.62 | 2.64 | 2.64 | / | / |
|  | 6 | 3.14 | 3.34 | 3.16 | 2.44 | 3.50 | 3.49 |
|  | 7 | / | 2.48 | 2.66 | 2.77 | / | / |
|  | 8 | 2.37 | 3.42 | 2.44 | 3.66 | 3.66 | 3.09 |
|  | 9 | 3.03 | 2.61 | 2.22 | 2.45 | / | / |
|  | 10 | / | 3.11 | 2.73 | 2.95 | 3.67 | 3.71 |
|  | 11 | 3.66 | 2.79 | 2.41 | 2.74 | / | / |
|  | 12 | 2.30 | 2.61 | 2.63 | 3.35 | / | / |
|  | Means±SD | 2.91±0.41 | 2.81±0.35 | 2.60±0.32 | 2.68±0.46 | 3.47±0.21 | 3.46±0.26 |
| *Lactobacillus* | 1 | 4.95 | 4.22 | 4.80 | 4.49 | 4.25 | 3.91 |
|  | 2 | 4.39 | 4.33 | 4.70 | 4.19 | 4.16 | 3.85 |
|  | 3 | 4.23 | 4.32 | 4.99 | 4.44 | / | / |
|  | 4 | 4.18 | 4.43 | 4.79 | 4.20 | / | / |
|  | 6 | 4.43 | 4.30 | 5.86 | 4.64 | 4.22 | 3.70 |
|  | 7 | / | 4.25 | 4.55 | 4.73 | / | / |
|  | 8 | 4.56 | 4.30 | 4.77 | 3.93 | 3.91 | 4.14 |
|  | 9 | 4.66 | 4.20 | 4.37 | 4.28 | / | / |
|  | 10 | / | 4.28 | 5.23 | 4.42 | 4.55 | 4.32 |
|  | 11 | 4.59 | 4.33 | 4.91 | 4.96 | / | / |
|  | 12 | 4.58 | 4.24 | 4.99 | 4.24 | / | / |
|  | Means±SD | 4.51±0.24 | 4.29±0.06 | 4.91±0.39 | 4.41±0.29 | 4.22±0.23 | 3.98±0.28 |
| *Streptococcus agalactiae* | 1 | 5.05 | 4.13 | 3.57 | 3.82 | 4.21 | / |
|  | 2 | 4.29 | 4.51 | 3.54 | 5.12 | 4.31 | 4.89 |
|  | 3 | 4.34 | 4.57 | 4.01 | 4.89 | / | / |
|  | 4 | 4.91 | 4.76 | 3.95 | 3.60 | / | / |
|  | 6 | 4.57 | 4.54 | 4.33 | 4.00 | 3.98 | 5.05 |
|  | 7 | / | 4.49 | 3.90 | 4.30 | / | / |
|  | 8 | 4.32 | 4.53 | 4.15 | 4.40 | 4.40 | 4.85 |
|  | 9 | 4.06 | 4.28 | 3.98 | 5.14 | / | / |
|  | 10 | / | 4.12 | 3.91 | 4.12 | 4.66 | 4.77 |
|  | 11 | 4.43 | 4.21 | 4.11 | 4.03 | / | / |
|  | 12 | 4.52 | 4.87 | 4.27 | 4.48 | / | / |
|  | Means±SD | 4.50±0.31 | 4.46±0.25 | 3.97±0.25 | 4.36±0.52 | 4.31±0.25 | 4.86±0.11 |
