# Supplementary material for: A Novel Lactobacilli-Based Teat Disinfectant for Improving Bacterial Communities in the Milks of Cow Teats with Subclinical Mastitis
Source: Front Microbiol. 2017 Sep 26;8:1782. doi: 10.3389/fmicb.2017.01782 (PMC5622921; doi:10.3389/fmicb.2017.01782)
Supplement: Supplementary file 3 [file Table_3.DOCX]

**Table S3. The relative abundances (in percentage) of bacteria at phylum, class, order, family, genus and species level**

| Taxonomy | LAB-0d | CD-0d | LAB-1d | CD-1d | LAB-10d | CD-10d | LAB-12d | CD-12d |
| --- | --- | --- | --- | --- | --- | --- | --- | --- |
| phylum |  |  |  |  |  |  |  |  |
| *Acidobacteria* | 0.020831±0.040149 | 0.007040±0.012065 | 0.014388±0.018761 | 0.008851±0.019525 | 0.006844±0.014731 | 0.014729±0.021963 | 0.028254±0.055909 | 0.021499±0.021074 |
| *Actinobacteria* | 0.140811±0.115485 | 0.144031±0.135012 | 0.231785±0.181701 | 0.187477±0.150510 | 0.288304±0.319455 | 0.216062±0.144751 | 0.182010±0.147908 | 0.283814±0.154079 |
| *Armatimonadetes* | 0 | 0 | 0 | 0 | 0.002792±0.008828 | 0 | 0.004067±0.009095 | 0.006818±0.013635 |
| *BRC1* | 0 | 0 | 0 | 0 | 0 | 0.003644±0.010308 | 0 | 0 |
| *Bacteroidetes* | 0.191627±0.194056 | 0.274072±0.196944 | 0.607797±0.495313 | 0.319646±0.383902 | 0.298619±0.322190 | 0.235101±0.167629 | 0.415079±0.390988 | 0.475674±0.346320 |
| *Chloroflexi* | 0 | 0 | 0 | 0 | 0 | 0.001787±0.005055 | 0 | 0 |
| *Cyanobacteria* | 0.001958±0.005874 | 0.001694±0.005619 | 0.030911±0.050226 | 0.001342±0.004450 | 0.008375±0.026485 | 0.006203±0.011662 | 0.017232±0.024878 | 0 |
| *Deferribacteres* | 0 | 0 | 0.001326±0.004194 | 0 | 0 | 0 | 0 | 0 |
| *Deinococcus-Thermus* | 0.035486±0.044573 | 0.040658±0.060326 | 0.046539±0.054824 | 0.065863±0.098935 | 0.048924±0.045936 | 0.036172±0.041053 | 0.111265±0.071115 | 0.110631±0.064486 |
| *Fibrobacteres* | 0 | 0 | 0.002459±0.007776 | 0 | 0 | 0 | 0 | 0 |
| *Firmicutes* | 91.256489±5.857502 | 85.130054±7.31451 | 91.495773±4.125706 | 92.490037±5.571955 | 91.765081±4.421997 | 93.224085±3.564273 | 92.759470±5.588502 | 90.998538±4.353469 |
| *Fusobacteria* | 0.004515±0.013545 | 0.002494±0.008272 | 0.010141±0.032069 | 0 | 0.011567±0.027275 | 0 | 0 | 0.015072±0.011827 |
| *Gemmatimonadetes* | 0 | 0 | 0 | 0.000671±0.002225 | 0 | 0.007289±0.020615 | 0 | 0 |
| *Lentisphaerae* | 0 | 0.002561±0.006443 | 0.001326±0.004194 | 0 | 0 | 0 | 0.004769±0.010664 | 0 |
| *Nitrospirae* | 0 | 0 | 0 | 0 | 0 | 0 | 0 | 0.003015±0.006031 |
| *OD1* | 0.001958±0.005874 | 0 | 0 | 0 | 0 | 0 | 0 | 0 |
| *Planctomycetes* | 0 | 0.003002±0.009955 | 0.001679±0.003582 | 0 | 0 | 0 | 0 | 0.003409±0.006818 |
| *Proteobacteria* | 8.277234±5.549309 | 14.186580±7.393903 | 7.096777±3.273652 | 6.733043±5.066908 | 7.122884±3.708116 | 6.142363±3.223904 | 6.229372±4.759299 | 7.931582±3.866961 |
| *Spirochaetes* | 0 | 0.002739±0.006614 | 0.008408±0.024074 | 0.011931±0.031407 | 0.002845±0.006479 | 0 | 0.020930±0.026519 | 0.004077±0.008154 |
| *TM6* | 0 | 0 | 0 | 0 | 0.001013±0.003204 | 0 | 0 | 0 |
| *TM7* | 0 | 0 | 0.012861±0.030862 | 0.008537±0.020134 | 0.003301±0.007093 | 0.002628±0.007434 | 0.008510±0.015519 | 0.011666±0.017076 |
| *Tenericutes* | 0.014770±0.019972 | 0.026576±0.032698 | 0.071221±0.152778 | 0.044588±0.089065 | 0.062886±0.094908 | 0.014791±0.020375 | 0.019223±0.026390 | 0.071192±0.058831 |
| *Verrucomicrobia* | 0.001129±0.003386 | 0 | 0.004667±0.008837 | 0.002765±0.009170 | 0.013959±0.044141 | 0 | 0.001356±0.003032 | 0.002620±0.005239 |
| *Viridiplantae* | 0 | 0.001001±0.003318 | 0.024925±0.052071 | 0.032257±0.092336 | 0.030784±0.071194 | 0.021112±0.023589 | 0.001356±0.003032 | 0 |
| *unclassified* | 0.053193±0.047000 | 0.177498±0.166861 | 0.337017±0.576626 | 0.092993±0.107017 | 0.331822±0.358233 | 0.074034±0.044697 | 0.197108±0.413014 | 0.060393±0.053478 |
| *Verrucomicrobia* | 0.001129±0.003386 | 0 | 0.004667±0.008837 | 0.002765±0.009170 | 0.013959±0.044141 | 0 | 0.001356±0.003032 | 0.002620±0.005239 |
| ***class*** |  |  |  |  |  |  |  |  |
| *4C0d-2* | 0 | 0 | 0 | 0 | 0.008375±0.026485 | 0 | 0.006386±0.014279 | 0 |
| *Acidobacteria-6* | 0.001958±0.005874 | 0.002522±0.008364 | 0 | 0.001876±0.006221 | 0 | 0.001876±0.006221 | 0 | 0 |
| *Acidobacteriia* | 0 | 0.001001±0.003318 | 0.000922±0.003058 | 0 | 0 | 0 | 0 | 0.003015±0.006031 |
| *Actinobacteria* | 0.140811±0.115485 | 0.144031±0.135012 | 0.231785±0.190570 | 0.187477±0.150510 | 0.287291±0.319737 | 0.187477±0.150510 | 0.182010±0.147908 | 0.283814±0.154079 |
| *Actinomycetales* | 0 | 0 | 0 | 0 | 0.001013±0.003204 | 0 | 0 | 0 |
| *Alphaproteobacteria* | 0.605386±0.729107 | 1.805987±1.710007 | 0.187778±0.130089 | 0.192798±0.212157 | 0.105551±0.082619 | 0.192798±0.212157 | 0.161313±0.149710 | 0.214635±0.148225 |
| *Armatimonadia* | 0 | 0 | 0 | 0 | 0.002792±0.008828 | 0 | 0.004067±0.009095 | 0 |
| *Bacilli* | 90.901983±5.977019 | 84.657468±7.219394 | 90.002353±5.412264 | 91.408523±6.438398 | 90.365064±5.982295 | 91.408523±6.438398 | 91.962686±6.012224 | 89.868143±4.612613 |
| *Bacteroidetes* | 0.002257±0.006772 | 0.010330±0.014872 | 0.027231±0.037741 | 0.010676±0.018673 | 0.011894±0.015996 | 0.010676±0.018673 | 0.009490±0.021221 | 0.015345±0.012887 |
| *Bacteroidia* | 0.076564±0.088050 | 0.128484±0.144762 | 0.377344±0.377796 | 0.203670±0.291557 | 0.147652±0.210259 | 0.203670±0.291557 | 0.268343±0.318365 | 0.282209±0.288331 |
| *Betaproteobacteria* | 1.330471±1.506256 | 3.880135±3.047743 | 0.643841±0.589854 | 0.422131±0.480009 | 0.279234±0.184293 | 0.422131±0.480009 | 0.429456±0.519750 | 0.651312±0.432542 |
| *Chloracidobacteria* | 0.001129±0.003386 | 0.001848±0.004128 | 0.006495±0.011273 | 0.002553±0.008467 | 0 | 0.002553±0.008467 | 0.002711±0.006063 | 0.018483±0.016291 |
| *Chloroplast* | 0.001958±0.005874 | 0.001694±0.005619 | 0.000631±0.002094 | 0.000671±0.002225 | 0 | 0.000671±0.002225 | 0.001356±0.003032 | 0 |
| *Clostridia* | 0.323752±0.222456 | 0.449111±0.368207 | 1.381181±1.649756 | 1.006630±1.265145 | 1.343293±2.923989 | 1.006630±1.265145 | 0.746260±0.646855 | 1.039705±0.743774 |
| *Cytophagia* | 0.001129±0.003386 | 0.003395±0.009910 | 0.005336±0.012528 | 0 | 0.001013±0.003204 | 0 | 0 | 0.003015±0.006031 |
| *Deferribacteres* | 0 | 0 | 0.001326±0.004398 | 0 | 0 | 0 | 0 | 0 |
| *Deinococci* | 0.035486±0.044573 | 0.040658±0.060326 | 0.046539±0.057500 | 0.065863±0.098935 | 0.048924±0.045936 | 0.065863±0.098935 | 0.111265±0.071115 | 0.110631±0.064486 |
| *Deltaproteobacteria* | 0.003396±0.006739 | 0.015647±0.023637 | 0.041422±0.096761 | 0.004428±0.009993 | 0.013959±0.044141 | 0.004428±0.009993 | 0.032388±0.041872 | 0.011936±0.015103 |
| *Epsilonproteobacteria* | 0.003652±0.007272 | 0.005016±0.011160 | 0.008826±0.013187 | 0.005099±0.009913 | 0.011716±0.019896 | 0.005099±0.009913 | 0.011482±0.013098 | 0.021769±0.009088 |
| *Erysipelotrichi* | 0.017424±0.031776 | 0.017711±0.027926 | 0.097663±0.186061 | 0.053405±0.055714 | 0.051035±0.073237 | 0.053405±0.055714 | 0.045077±0.043534 | 0.059430±0.040113 |
| *Erysipelotrichia* | 0 | 0 | 0 | 0 | 0 | 0 | 0 | 0.002620±0.005239 |
| *Fibrobacteria* | 0 | 0 | 0.002459±0.008156 | 0 | 0 | 0 | 0 | 0 |
| *Fimbriimonadia* | 0 | 0 | 0 | 0 | 0 | 0 | 0 | 0.006818±0.013635 |
| *Flavobacteria* | 0.016250±0.022068 | 0.032080±0.027492 | 0.028991±0.036678 | 0.019220±0.032640 | 0.019068±0.032233 | 0.019220±0.032640 | 0.021020±0.025239 | 0.036177±0.029796 |
| *Flavobacteriia* | 0.084008±0.096975 | 0.079886±0.049331 | 0.146898±0.102392 | 0.065753±0.050363 | 0.103579±0.083311 | 0.065753±0.050363 | 0.108484±0.079917 | 0.114809±0.079495 |
| *Fusobacteria* | 0.004515±0.013545 | 0.002494±0.008272 | 0.010141±0.033634 | 0 | 0.005984±0.012650 | 0 | 0 | 0.015072±0.011827 |
| *Fusobacteriia* | 0 | 0 | 0 | 0 | 0.005583±0.017656 | 0 | 0 | 0 |
| *Gammaproteobacteria* | 6.325760±3.719568 | 8.463712±3.292435 | 6.213127±2.951924 | 6.108586±4.435474 | 6.712425±3.514888 | 6.108586±4.435474 | 5.594733±4.117869 | 7.031930±3.353874 |
| *Gemm-3* | 0 | 0 | 0 | 0.000671±0.002225 | 0 | 0.000671±0.002225 | 0 | 0 |
| *Lentisphaeria* | 0 | 0.002561±0.006443 | 0.001326±0.004398 | 0 | 0 | 0 | 0.004769±0.010664 | 0 |
| *Mollicutes* | 0.014770±0.019972 | 0.025729±0.032514 | 0.069895±0.160810 | 0.043917±0.088420 | 0.062886±0.094908 | 0.043917±0.088420 | 0.019223±0.026390 | 0.063039±0.064894 |
| *Nitrospira* | 0 | 0 | 0 | 0 | 0 | 0 | 0 | 0.003015±0.006031 |
| *Nostocophycideae* | 0 | 0 | 0.002766±0.009173 | 0 | 0 | 0 | 0 | 0 |
| *Opitutae* | 0.001129±0.003386 | 0 | 0 | 0 | 0 | 0 | 0 | 0 |
| *Oscillatoriophycideae* | 0 | 0 | 0.016305±0.028009 | 0.000671±0.002225 | 0 | 0.000671±0.002225 | 0.006779±0.015158 | 0 |
| *Phycisphaerae* | 0 | 0.001001±0.003318 | 0 | 0 | 0 | 0 | 0 | 0 |
| *Planctomycetia* | 0 | 0.002001±0.006637 | 0.001679±0.003757 | 0 | 0 | 0 | 0 | 0.003409±0.006818 |
| *RF3* | 0 | 0.000847±0.002810 | 0.001326±0.004398 | 0.000671±0.002225 | 0 | 0.000671±0.002225 | 0 | 0.008154±0.016308 |
| *Rhodothermi* | 0 | 0 | 0.000757±0.002509 | 0 | 0 | 0 | 0 | 0 |
| *SJA-4* | 0 | 0 | 0 | 0 | 0.001013±0.003204 | 0 | 0 | 0 |
| *Solibacteres* | 0 | 0 | 0 | 0.000671±0.002225 | 0.002792±0.008828 | 0.000671±0.002225 | 0.019157±0.042837 | 0 |
| *Sphingobacteriia* | 0.009260±0.019208 | 0.019897±0.020651 | 0.019727±0.032403 | 0.020328±0.030322 | 0.013471±0.017519 | 0.020328±0.030322 | 0.007741±0.013836 | 0.024118±0.019864 |
| *Spirochaetia* | 0 | 0.002739±0.006614 | 0.008408±0.025250 | 0.011931±0.031407 | 0.002845±0.006479 | 0.011931±0.031407 | 0.020930±0.026519 | 0.004077±0.008154 |
| *Synechococcophycideae* | 0 | 0 | 0 | 0 | 0 | 0 | 0.002711±0.006063 | 0 |
| *TA18* | 0.001702±0.005105 | 0 | 0 | 0 | 0 | 0 | 0 | 0 |
| *TM7-3* | 0 | 0 | 0.012861±0.032368 | 0.008537±0.020134 | 0.003301±0.007093 | 0.008537±0.020134 | 0.008510±0.015519 | 0.011666±0.017076 |
| *Verrucomicrobiae* | 0 | 0 | 0.004667±0.009268 | 0.002765±0.009170 | 0.013959±0.044141 | 0.002765±0.009170 | 0.001356±0.003032 | 0.002620±0.005239 |
| *ZB2* | 0.001958±0.005874 | 0 | 0 | 0 | 0 | 0 | 0 | 0 |
| *iii1-8* | 0 | 0 | 0 | 0 | 0 | 0 | 0.006386±0.014279 | 0 |
| *unclassified* | 0.093293±0.048804 | 0.202015±0.186202 | 0.397993±0.619974 | 0.150480±0.148001 | 0.374291±0.365925 | 0.150480±0.148001 | 0.203911±0.412963 | 0.089033±0.088745 |
| Order |  |  |  |  |  |  |  |  |
| *32-20* | 0 | 0 | 0 | 0 | 0 | 0 | 0.006386±0.014279 | 0 |
| *34P16* | 0 | 0 | 0 | 0 | 0.001510±0.004529 | 0 | 0 | 0 |
| *Acholeplasmatales* | 0.001702±0.005105 | 0.005350±0.010064 | 0.002015±0.004503 | 0 | 0.004013±0.012040 | 0 | 0.019223±0.026390 | 0.005239±0.010479 |
| *Acidimicrobiales* | 0 | 0 | 0.000922±0.003058 | 0 | 0 | 0.003239±0.009718 | 0 | 0 |
| *Acidobacteriales* | 0 | 0 | 0.000922±0.003058 | 0 | 0 | 0 | 0 | 0 |
| *Actinomycetales* | 0.140811±0.115485 | 0.144031±0.135012 | 0.229942±0.190329 | 0.187477±0.150510 | 0.312135±0.328736 | 0.192653±0.130265 | 0.182010±0.147908 | 0.280405±0.150081 |
| *Aeromonadales* | 0.015101±0.022239 | 0.029171±0.024782 | 0.050591±0.058364 | 0.044466±0.055475 | 0.062063±0.168720 | 0.030294±0.055958 | 0.019706±0.020126 | 0.020584±0.022377 |
| *Alteromonadales* | 0.009069±0.011983 | 0.003770±0.009451 | 0.004916±0.008082 | 0.004640±0.010554 | 0.007255±0.010007 | 0.012096±0.021960 | 0.011809±0.016259 | 0.035243±0.049402 |
| *Anaeroplasmatales* | 0 | 0 | 0 | 0.001876±0.006221 | 0 | 0 | 0 | 0 |
| *Armatimonadales* | 0 | 0 | 0 | 0 | 0.003102±0.009306 | 0 | 0.004067±0.009095 | 0 |
| *BD7-3* | 0 | 0 | 0.000631±0.002094 | 0 | 0 | 0 | 0 | 0 |
| *Bacillales* | 85.332390±9.032124 | 79.528259±6.626624 | 82.791070±7.275118 | 84.472169±13.197108 | 83.599449±9.976915 | 88.023787±7.101421 | 84.942295±12.854064 | 84.087102±6.521261 |
| *Bacteroidales* | 0.078822±0.088445 | 0.138813±0.158926 | 0.404575±0.409072 | 0.214345±0.300220 | 0.173735±0.231915 | 0.128114±0.121774 | 0.277833±0.320182 | 0.297554±0.299527 |
| *Bdellovibrionales* | 0.001695±0.005084 | 0.013253±0.024176 | 0.005088±0.009729 | 0.002553±0.008467 | 0.015510±0.046529 | 0.005576±0.011249 | 0.024253±0.040496 | 0.007859±0.015718 |
| *Bifidobacteriales* | 0 | 0 | 0 | 0 | 0 | 0 | 0 | 0.003409±0.006818 |
| *Burkholderiales* | 1.319151±1.502619 | 3.855573±3.068311 | 0.628296±0.591575 | 0.404002±0.458792 | 0.264855±0.181107 | 0.193266±0.191106 | 0.429456±0.519750 | 0.623781±0.438127 |
| *CCU21* | 0.001958±0.005874 | 0 | 0 | 0 | 0 | 0 | 0 | 0 |
| *CW040* | 0 | 0 | 0.012861±0.032368 | 0.006661±0.014964 | 0 | 0 | 0.002385±0.005332 | 0.006031±0.012061 |
| *Campylobacterales* | 0.003652±0.007272 | 0.005016±0.011160 | 0.008826±0.013187 | 0.005099±0.009913 | 0.013018±0.020646 | 0.003239±0.009718 | 0.011482±0.013098 | 0.021769±0.009088 |
| *Caryophyllales* | 0 | 0 | 0.000631±0.002094 | 0.001112±0.003687 | 0 | 0.001589±0.004766 | 0 | 0 |
| *Caulobacterales* | 0.029591±0.032030 | 0.057647±0.064467 | 0.021977±0.030381 | 0.034626±0.046523 | 0.013976±0.017030 | 0.019669±0.025501 | 0.022703±0.021597 | 0.033558±0.030795 |
| *Chloracidobacterales* | 0.001129±0.003386 | 0.001848±0.004128 | 0.006495±0.011273 | 0.002553±0.008467 | 0 | 0.003498±0.010495 | 0.002711±0.006063 | 0.018483±0.016291 |
| *Chlorophyta* | 0 | 0 | 0.000631±0.002094 | 0 | 0 | 0 | 0 | 0 |
| *Chromatiales* | 0.002594±0.007781 | 0.000847±0.002810 | 0.011050±0.015735 | 0.006918±0.012593 | 0 | 0.002336±0.007009 | 0 | 0.006029±0.007079 |
| *Chroococcales* | 0 | 0 | 0.016305±0.028009 | 0.000671±0.002225 | 0 | 0.001589±0.004766 | 0.006779±0.015158 | 0 |
| *Clostridiales* | 0.323752±0.222456 | 0.449111±0.368207 | 1.381181±1.649756 | 1.006630±1.265145 | 1.489009±3.062605 | 0.572791±0.559352 | 0.746260±0.646855 | 1.039705±0.743774 |
| *Coriobacteriales* | 0 | 0 | 0 | 0 | 0 | 0.003239±0.009718 | 0 | 0 |
| *Cytophagales* | 0.001129±0.003386 | 0.003395±0.009910 | 0.005336±0.012528 | 0 | 0.001126±0.003377 | 0.003044±0.009132 | 0 | 0.003015±0.006031 |
| *Deferribacterales* | 0 | 0 | 0.001326±0.004398 | 0 | 0 | 0 | 0 | 0 |
| *Deinococcales* | 0.035486±0.044573 | 0.039988±0.060772 | 0.046539±0.057500 | 0.065863±0.098935 | 0.047282±0.048411 | 0.039230±0.039482 | 0.111265±0.071115 | 0.110631±0.064486 |
| *Desulfovibrionales* | 0 | 0.002394±0.006635 | 0.005486±0.012284 | 0.001876±0.006221 | 0 | 0 | 0.002711±0.006063 | 0 |
| *Desulfuromonadales* | 0.001702±0.005105 | 0 | 0 | 0 | 0 | 0.015205±0.031638 | 0.001356±0.003032 | 0 |
| *Enterobacteriales* | 0.066121±0.082804 | 0.226838±0.268628 | 0.502691±0.755624 | 0.766470±2.116286 | 0.378742±0.379135 | 0.249886±0.293279 | 0.860497±1.411019 | 0.169340±0.107265 |
| *Entomoplasmatales* | 0 | 0.001892±0.006275 | 0 | 0 | 0 | 0 | 0 | 0 |
| *Erysipelotrichales* | 0.017424±0.031776 | 0.017711±0.027926 | 0.097663±0.186061 | 0.053405±0.055714 | 0.053167±0.077350 | 0.036557±0.035454 | 0.045077±0.043534 | 0.062050±0.044197 |
| *Fabales* | 0 | 0 | 0.002766±0.009173 | 0 | 0 | 0 | 0 | 0 |
| *Fibrobacterales* | 0 | 0 | 0.002459±0.008156 | 0 | 0 | 0 | 0 | 0 |
| *Fimbriimonadales* | 0 | 0 | 0 | 0 | 0 | 0 | 0 | 0.006818±0.013635 |
| *Flavobacteriales* | 0.100258±0.117068 | 0.111966±0.067732 | 0.175889±0.126158 | 0.084973±0.071615 | 0.125658±0.109187 | 0.090385±0.074817 | 0.129504±0.100980 | 0.150986±0.105537 |
| *Fusobacteriales* | 0.004515±0.013545 | 0.002494±0.008272 | 0.010141±0.033634 | 0 | 0.012852±0.028606 | 0 | 0 | 0.015072±0.011827 |
| *GMD14H09* | 0 | 0 | 0 | 0 | 0 | 0 | 0 | 0.004077±0.008154 |
| *Gallionellales* | 0 | 0 | 0 | 0 | 0.001510±0.004529 | 0.001589±0.004766 | 0 | 0 |
| *Hydrogenophilales* | 0 | 0.000393±0.001304 | 0 | 0.001876±0.006221 | 0 | 0 | 0 | 0 |
| *Lactobacillales* | 5.505570±3.374592 | 5.078756±2.471755 | 7.055893±2.906388 | 6.860234±8.950989 | 6.150025±4.233887 | 4.758755±3.656910 | 6.982881±7.765816 | 5.715285±2.408973 |
| *Legionellales* | 0.001958±0.005874 | 0 | 0.001326±0.004398 | 0.003023±0.010027 | 0 | 0 | 0.002385±0.005332 | 0 |
| *ML615J-28* | 0 | 0.000847±0.002810 | 0.001326±0.004398 | 0.000671±0.002225 | 0 | 0.006479±0.019436 | 0 | 0.008154±0.016308 |
| *Malvales* | 0 | 0 | 0.020265±0.055481 | 0.031145±0.092674 | 0.033079±0.074088 | 0.017177±0.021746 | 0.001356±0.003032 | 0 |
| *Methylophilales* | 0 | 0.006025±0.019984 | 0 | 0 | 0 | 0 | 0 | 0 |
| *Micrococcineae* | 0 | 0.001001±0.003318 | 0 | 0 | 0 | 0 | 0 | 0.003015±0.006031 |
| *Mycoplasmatales* | 0 | 0 | 0 | 0.001876±0.006221 | 0 | 0 | 0 | 0 |
| *Myxococcales* | 0 | 0 | 0.030849±0.094331 | 0 | 0 | 0.001589±0.004766 | 0 | 0 |
| *Neisseriales* | 0.009435±0.012628 | 0.002517±0.004388 | 0.007555±0.012930 | 0.007302±0.010627 | 0.012303±0.016583 | 0.022284±0.037923 | 0 | 0.027531±0.046420 |
| *Nitrospirales* | 0 | 0 | 0 | 0 | 0 | 0 | 0 | 0.003015±0.006031 |
| *Oceanospirillales* | 0.001666±0.004999 | 0.005219±0.011617 | 0.004025±0.008039 | 0.010934±0.019747 | 0.025632±0.049852 | 0 | 0.001356±0.003032 | 0.028173±0.027528 |
| *Oscillatoriales* | 0 | 0 | 0.005531±0.018346 | 0 | 0 | 0.002336±0.007009 | 0 | 0 |
| *PHOS-HD29* | 0.001702±0.005105 | 0 | 0 | 0 | 0 | 0 | 0 | 0 |
| *Pasteurellales* | 0.002823±0.005745 | 0.005969±0.019797 | 0.005102±0.008861 | 0.001876±0.006221 | 0.025260±0.064678 | 0.004672±0.014017 | 0.004769±0.010664 | 0.009044±0.006165 |
| *Phycisphaerales* | 0 | 0.001001±0.003318 | 0 | 0 | 0 | 0 | 0 | 0 |
| *Pinales* | 0 | 0.001001±0.003318 | 0.001263±0.004188 | 0 | 0.001126±0.003377 | 0 | 0 | 0 |
| *Pirellulales* | 0 | 0 | 0.000757±0.002509 | 0 | 0 | 0 | 0 | 0 |
| *Planctomycetales* | 0 | 0.002001±0.006637 | 0.000922±0.003058 | 0 | 0 | 0 | 0 | 0.003409±0.006818 |
| *Propionibacterineae* | 0 | 0 | 0 | 0 | 0.001126±0.003377 | 0 | 0 | 0 |
| *Pseudanabaenales* | 0 | 0 | 0 | 0 | 0 | 0 | 0.002711±0.006063 | 0 |
| *Pseudomonadales* | 6.091808±3.576248 | 8.019184±3.270594 | 5.379404±2.515430 | 5.037816±2.818418 | 6.323070±3.340692 | 5.170057±2.503872 | 4.288839±2.761379 | 5.981485±2.647112 |
| *Puniceicoccales* | 0.001129±0.003386 | 0 | 0 | 0 | 0 | 0 | 0 | 0 |
| *RF32* | 0 | 0.002893±0.006799 | 0 | 0 | 0 | 0 | 0 | 0.002620±0.005239 |
| *RF39* | 0.013068±0.020564 | 0.018488±0.028627 | 0.067880±0.160656 | 0.040166±0.077147 | 0.065860±0.100170 | 0.006668±0.010344 | 0 | 0.057799±0.054824 |
| *Rhizobiales* | 0.495922±0.626402 | 1.648191±1.649027 | 0.032598±0.035186 | 0.049854±0.051415 | 0.038170±0.044672 | 0.028909±0.041277 | 0.040522±0.050282 | 0.085626±0.107803 |
| *Rhodobacterales* | 0.043062±0.076797 | 0.040656±0.049713 | 0.066807±0.049981 | 0.068813±0.095228 | 0.034374±0.051714 | 0.041904±0.040389 | 0.069308±0.061427 | 0.061624±0.038558 |
| *Rhodocyclales* | 0.001885±0.005655 | 0.003833±0.008989 | 0.007990±0.019317 | 0.008952±0.016231 | 0.003284±0.006917 | 0 | 0 | 0 |
| *Rhodospirillales* | 0.003287±0.006922 | 0.003369±0.008553 | 0.015441±0.025024 | 0.007984±0.015393 | 0.002251±0.006754 | 0.006479±0.019436 | 0.009097±0.014048 | 0.006424±0.007446 |
| *Rhodothermales* | 0 | 0 | 0.000757±0.002509 | 0 | 0 | 0 | 0 | 0 |
| *Rickettsiales* | 0 | 0.001694±0.005619 | 0 | 0.001531±0.005079 | 0 | 0 | 0 | 0.002620±0.005239 |
| *Rubrobacterales* | 0 | 0 | 0.000922±0.003058 | 0 | 0 | 0 | 0 | 0 |
| *SBR1031* | 0 | 0 | 0 | 0 | 0 | 0.001589±0.004766 | 0 | 0 |
| *Solibacterales* | 0 | 0 | 0 | 0.000671±0.002225 | 0.003102±0.009306 | 0 | 0.019157±0.042837 | 0 |
| *Sphingobacteriales* | 0.009260±0.019208 | 0.019897±0.020651 | 0.019727±0.032403 | 0.020328±0.030322 | 0.014968±0.017890 | 0.001589±0.004766 | 0.007741±0.013836 | 0.024118±0.019864 |
| *Sphingomonadales* | 0.033523±0.032197 | 0.049645±0.046502 | 0.050323±0.042035 | 0.029990±0.034510 | 0.028509±0.021690 | 0.041312±0.045713 | 0.019683±0.032372 | 0.019149±0.006520 |
| *Spirochaetales* | 0 | 0.002739±0.006614 | 0.008408±0.025250 | 0.011931±0.031407 | 0.003161±0.006790 | 0 | 0.020930±0.026519 | 0.004077±0.008154 |
| *Stigonematales* | 0 | 0 | 0.002766±0.009173 | 0 | 0 | 0 | 0 | 0 |
| *Streptophyta* | 0.001958±0.005874 | 0.001694±0.005619 | 0 | 0.000671±0.002225 | 0 | 0.001589±0.004766 | 0.001356±0.003032 | 0 |
| *Syntrophobacterales* | 0 | 0 | 0 | 0 | 0 | 0 | 0.004067±0.009095 | 0 |
| *Thermales* | 0 | 0.000669±0.002220 | 0 | 0 | 0 | 0 | 0 | 0 |
| *Tremblayales* | 0 | 0.011006±0.036503 | 0 | 0 | 0 | 0 | 0 | 0 |
| *Verrucomicrobiales* | 0 | 0 | 0.004667±0.009268 | 0.002765±0.009170 | 0.015510±0.046529 | 0 | 0.001356±0.003032 | 0.002620±0.005239 |
| *Vibrionales* | 0 | 0.000393±0.001304 | 0.000631±0.002094 | 0 | 0.006204±0.018612 | 0.003539±0.010616 | 0.006802±0.011792 | 0 |
| *Victivallales* | 0 | 0.002561±0.006443 | 0.001326±0.004398 | 0 | 0 | 0 | 0.004769±0.010664 | 0 |
| *Xanthomonadales* | 0.134619±0.123676 | 0.164056±0.120032 | 0.249193±0.286437 | 0.223668±0.303473 | 0.147004±0.105620 | 0.088478±0.067657 | 0.395710±0.450353 | 0.767749±0.534160 |
| *YS2* | 0 | 0 | 0 | 0 | 0.009306±0.027917 | 0 | 0.006386±0.014279 | 0 |
| *iii1-15* | 0 | 0.002522±0.008364 | 0 | 0.001876±0.006221 | 0 | 0.003239±0.009718 | 0 | 0 |
| *unclassified* | 0.159275±0.101855 | 0.262409±0.223815 | 0.527125±0.604063 | 0.205665±0.168964 | 0.476715±0.374900 | 0.158484±0.079919 | 0.249051±0.389435 | 0.177722±0.117591 |
| family |  |  |  |  |  |  |  |  |
| *0319-6A21* | 0 | 0 | 0 | 0 | 0 | 0 | 0 | 0.003015±0.006031 |
| *A4b* | 0 | 0 | 0 | 0 | 0 | 0.001589±0.004766 | 0 | 0 |
| *Acetobacteraceae* | 0 | 0 | 0.004003±0.005834 | 0.006201±0.015632 | 0 | 0 | 0.009097±0.014048 | 0 |
| *Acholeplasmataceae* | 0.001702±0.005105 | 0.005350±0.010064 | 0.002015±0.004503 | 0 | 0.004013±0.012040 | 0 | 0.019223±0.026390 | 0.005239±0.010479 |
| *Acidimicrobiaceae* | 0 | 0 | 0.000922±0.003058 | 0 | 0 | 0 | 0 | 0 |
| *Acidobacteriaceae* | 0 | 0 | 0.000922±0.003058 | 0 | 0 | 0 | 0 | 0 |
| *Actinomycetaceae* | 0 | 0 | 0.001124±0.003727 | 0 | 0 | 0 | 0 | 0 |
| *Actinomycetales* | 0.099560±0.092449 | 0.111039±0.100145 | 0.126247±0.083169 | 0.094820±0.054805 | 0.116641±0.081006 | 0.107357±0.077167 | 0.110203±0.096164 | 0.150475±0.117549 |
| *Aerococcaceae* | 0.027946±0.028827 | 0.018004±0.020380 | 0.047121±0.048315 | 0.089087±0.151951 | 0.123888±0.191948 | 0.095238±0.155825 | 0.055733±0.050060 | 0.136382±0.137081 |
| *Aeromonadaceae* | 0.010576±0.022982 | 0.013687±0.018186 | 0.037034±0.047109 | 0.039367±0.051107 | 0.051809±0.148282 | 0.025214±0.047875 | 0.011548±0.016207 | 0.006029±0.007079 |
| *Alcaligenaceae* | 0 | 0.003341±0.008466 | 0.008194±0.011439 | 0.008794±0.012990 | 0.019426±0.024921 | 0.002336±0.007009 | 0.002711±0.006063 | 0.009712±0.006927 |
| *Alcanivoracaceae* | 0 | 0.002678±0.008882 | 0 | 0 | 0 | 0 | 0 | 0 |
| *Alteromonadaceae* | 0.004781±0.007421 | 0.001001±0.003318 | 0.001093±0.003625 | 0 | 0.002968±0.008903 | 0.001589±0.004766 | 0.007741±0.013836 | 0.011270±0.013078 |
| *Amaranthaceae* | 0 | 0 | 0.000631±0.002094 | 0.001112±0.003687 | 0 | 0.001589±0.004766 | 0 | 0 |
| *Anaeroplasmataceae* | 0 | 0 | 0 | 0.001876±0.006221 | 0 | 0 | 0 | 0 |
| *Armatimonadaceae* | 0 | 0 | 0 | 0 | 0.003102±0.009306 | 0 | 0.004067±0.009095 | 0 |
| *Aurantimonadaceae* | 0 | 0 | 0 | 0.001112±0.003687 | 0 | 0 | 0 | 0 |
| *BS11* | 0 | 0 | 0.001093±0.003625 | 0.004899±0.011259 | 0 | 0 | 0 | 0.002620±0.005239 |
| *Bacillaceae* | 83.621810±9.189276 | 76.852423±7.004279 | 80.202782±7.832331 | 81.419804±13.002171 | 80.473273±9.361627 | 83.452624±7.031498 | 81.951731±12.086129 | 80.271261±5.907278 |
| *Bacteroidaceae* | 0.011675±0.029206 | 0.028351±0.032530 | 0.090489±0.104044 | 0.049553±0.080089 | 0.053922±0.108375 | 0.038555±0.050097 | 0.101236±0.126811 | 0.052043±0.056793 |
| *Bacteroidales* | 0.002257±0.006772 | 0.008438±0.014728 | 0.026600±0.038043 | 0.010005±0.018934 | 0.013216±0.016377 | 0.002743±0.008230 | 0.009490±0.021221 | 0.015345±0.012887 |
| *Bdellovibrionaceae* | 0.001695±0.005084 | 0.013253±0.024176 | 0.005088±0.009729 | 0.002553±0.008467 | 0.015510±0.046529 | 0.005576±0.011249 | 0.024253±0.040496 | 0.007859±0.015718 |
| *Beijerinckiaceae* | 0 | 0.000847±0.002810 | 0 | 0.002553±0.008467 | 0 | 0 | 0 | 0 |
| *Bifidobacteriaceae* | 0 | 0 | 0 | 0 | 0 | 0 | 0 | 0.003409±0.006818 |
| *Bogoriellaceae* | 0 | 0 | 0 | 0.001876±0.006221 | 0 | 0 | 0 | 0 |
| *Bradyrhizobiaceae* | 0.028691±0.036780 | 0.067730±0.076319 | 0.006936±0.011228 | 0.003665±0.008890 | 0 | 0 | 0 | 0.009316±0.010924 |
| *Brevibacteriaceae* | 0 | 0.001001±0.003318 | 0 | 0 | 0 | 0 | 0 | 0 |
| *Brucellaceae* | 0.003396±0.006739 | 0.002364±0.005832 | 0.004806±0.008602 | 0.004290±0.009906 | 0.009606±0.019342 | 0.001589±0.004766 | 0.002385±0.005332 | 0.010226±0.020453 |
| *Bryobacteraceae* | 0 | 0 | 0 | 0.000671±0.002225 | 0.003102±0.009306 | 0 | 0.019157±0.042837 | 0 |
| *Burkholderiaceae* | 0.867145±1.029072 | 2.836865±2.596233 | 0.321749±0.396785 | 0.218186±0.313977 | 0.077196±0.064293 | 0.086693±0.098794 | 0.309811±0.441466 | 0.419367±0.349693 |
| *Campylobacteraceae* | 0.003652±0.007272 | 0.005016±0.011160 | 0.008826±0.013187 | 0.005099±0.009913 | 0.013018±0.020646 | 0.003239±0.009718 | 0.011482±0.013098 | 0.021769±0.009088 |
| *Carnobacteriaceae* | 0.110825±0.076775 | 0.095246±0.070340 | 0.179561±0.203921 | 0.190750±0.139232 | 0.237170±0.261089 | 0.199994±0.203909 | 0.101010±0.045235 | 0.127553±0.055034 |
| *Caulobacteraceae* | 0.029591±0.032030 | 0.057647±0.064467 | 0.021977±0.030381 | 0.034626±0.046523 | 0.013976±0.017030 | 0.019669±0.025501 | 0.022703±0.021597 | 0.033558±0.030795 |
| *Cellulomonadaceae* | 0 | 0 | 0 | 0 | 0 | 0 | 0 | 0.003015±0.006031 |
| *Chitinophagaceae* | 0 | 0.003918±0.007542 | 0.001844±0.006115 | 0.000671±0.002225 | 0 | 0.001589±0.004766 | 0.006386±0.014279 | 0 |
| *Chloracidobacteraceae* | 0.001129±0.003386 | 0.001848±0.004128 | 0.006495±0.011273 | 0.002553±0.008467 | 0 | 0.003498±0.010495 | 0.002711±0.006063 | 0.018483±0.016291 |
| *Christensenellaceae* | 0 | 0.003671±0.009981 | 0.003284±0.008837 | 0 | 0 | 0.003239±0.009718 | 0.002385±0.005332 | 0 |
| *Chromatiaceae* | 0.002594±0.007781 | 0.000847±0.002810 | 0.011050±0.015735 | 0.006918±0.012593 | 0.002158±0.006474 | 0.002336±0.007009 | 0 | 0.006029±0.007079 |
| *Clostridiaceae* | 0.083657±0.059427 | 0.103705±0.068984 | 0.370631±0.548723 | 0.243983±0.313539 | 0.338323±0.592459 | 0.181263±0.218540 | 0.137595±0.080143 | 0.280631±0.146518 |
| *Clostridiales* | 0.001695±0.005084 | 0.003833±0.008989 | 0.002415±0.006073 | 0.003751±0.012441 | 0 | 0.002336±0.007009 | 0 | 0.006697±0.008091 |
| *ClostridialesFamilyXI* | 0 | 0 | 0 | 0.001531±0.005079 | 0 | 0 | 0 | 0.005239±0.010479 |
| *Comamonadaceae* | 0.248138±0.304058 | 0.752753±0.496790 | 0.084868±0.068185 | 0.066586±0.072496 | 0.074874±0.071289 | 0.055328±0.048516 | 0.052838±0.054370 | 0.063993±0.040064 |
| *Coriobacteriaceae* | 0 | 0 | 0 | 0 | 0 | 0.003239±0.009718 | 0 | 0 |
| *Corynebacteriaceae* | 0.006882±0.015755 | 0.013510±0.020274 | 0.050288±0.081058 | 0.039885±0.055252 | 0.116554±0.180687 | 0.045304±0.053743 | 0.020760±0.036141 | 0.080263±0.024169 |
| *Coxiellaceae* | 0 | 0 | 0.001326±0.004398 | 0 | 0 | 0 | 0 | 0 |
| *Cyclobacteriaceae* | 0 | 0.001394±0.003442 | 0.002270±0.007528 | 0 | 0 | 0 | 0 | 0.003015±0.006031 |
| *Cystobacteraceae* | 0 | 0 | 0.001513±0.005019 | 0 | 0 | 0 | 0 | 0 |
| *Cytophagaceae* | 0.001129±0.003386 | 0.002001±0.006637 | 0.003067±0.005698 | 0 | 0.001126±0.003377 | 0.003044±0.009132 | 0 | 0 |
| *Deferribacteraceae* | 0 | 0 | 0.001326±0.004398 | 0 | 0 | 0 | 0 | 0 |
| *Dehalobacteriaceae* | 0 | 0.000669±0.002220 | 0 | 0 | 0 | 0 | 0 | 0 |
| *Deinococcaceae* | 0.035486±0.044573 | 0.039988±0.060772 | 0.046539±0.057500 | 0.065193±0.096898 | 0.047282±0.048411 | 0.039230±0.039482 | 0.111265±0.071115 | 0.110631±0.064486 |
| *Dermabacteraceae* | 0 | 0 | 0.004080±0.009106 | 0 | 0 | 0 | 0 | 0.002620±0.005239 |
| *Dermatophilaceae* | 0 | 0 | 0 | 0.002910±0.009651 | 0 | 0 | 0 | 0 |
| *Desulfomicrobiaceae* | 0 | 0 | 0.000757±0.002509 | 0 | 0 | 0 | 0.002711±0.006063 | 0 |
| *Desulfovibrionaceae* | 0 | 0.002394±0.006635 | 0.004729±0.010531 | 0.001876±0.006221 | 0 | 0 | 0 | 0 |
| *Dietziaceae* | 0 | 0 | 0.002459±0.008156 | 0.001876±0.006221 | 0.004409±0.008752 | 0.006997±0.020991 | 0 | 0.002620±0.005239 |
| *Enterobacteriaceae* | 0.066121±0.082804 | 0.226838±0.268628 | 0.502691±0.755624 | 0.766470±2.116286 | 0.378742±0.379135 | 0.249886±0.293279 | 0.860497±1.411019 | 0.169340±0.107265 |
| *Enterococcaceae* | 0.015622±0.017274 | 0.020899±0.043327 | 0.031305±0.021651 | 0.009171±0.020343 | 0.026121±0.022295 | 0.007164±0.011372 | 0.013530±0.013971 | 0.023204±0.027393 |
| *Erysipelotrichaceae* | 0.017424±0.031776 | 0.017711±0.027926 | 0.097663±0.186061 | 0.053405±0.055714 | 0.053167±0.077350 | 0.036557±0.035454 | 0.045077±0.043534 | 0.062050±0.044197 |
| *Erythrobacteraceae* | 0.004288±0.008746 | 0.000847±0.002810 | 0.003372±0.005860 | 0.002553±0.008467 | 0.001126±0.003377 | 0.008067±0.019425 | 0.001356±0.003032 | 0 |
| *Eubacteriaceae* | 0.020544±0.039385 | 0.028661±0.040290 | 0.092690±0.100453 | 0.057080±0.066203 | 0.080262±0.152890 | 0.073099±0.102860 | 0.079586±0.117860 | 0.081419±0.058526 |
| *Exiguobacteraceae* | 0 | 0 | 0 | 0.001112±0.003687 | 0 | 0 | 0 | 0 |
| *F16* | 0 | 0 | 0.012861±0.032368 | 0.006661±0.014964 | 0 | 0 | 0.002385±0.005332 | 0.006031±0.012061 |
| *Fabaceae* | 0 | 0 | 0.002766±0.009173 | 0 | 0 | 0 | 0 | 0 |
| *Fibrobacteraceae* | 0 | 0 | 0.002459±0.008156 | 0 | 0 | 0 | 0 | 0 |
| *Fimbriimonadaceae* | 0 | 0 | 0 | 0 | 0 | 0 | 0 | 0.006818±0.013635 |
| *Flavobacteriaceae* | 0.097664±0.118651 | 0.111966±0.067732 | 0.175889±0.126158 | 0.078887±0.069802 | 0.125658±0.109187 | 0.090385±0.074817 | 0.129504±0.100980 | 0.150986±0.105537 |
| *Fusobacteriaceae* | 0.004515±0.013545 | 0.002494±0.008272 | 0.010141±0.033634 | 0 | 0.006648±0.013230 | 0 | 0 | 0.015072±0.011827 |
| *Gallionellaceae* | 0 | 0 | 0 | 0 | 0.001510±0.004529 | 0.001589±0.004766 | 0 | 0 |
| *Geodermatophilaceae* | 0 | 0 | 0.000922±0.003058 | 0 | 0 | 0 | 0 | 0 |
| *HTCC2188* | 0 | 0 | 0.000922±0.003058 | 0 | 0 | 0 | 0.002711±0.006063 | 0 |
| *Halomonadaceae* | 0.001666±0.004999 | 0.002541±0.008429 | 0.004025±0.008039 | 0.010934±0.019747 | 0.025632±0.049852 | 0 | 0.001356±0.003032 | 0.028173±0.027528 |
| *Hydrogenophilaceae* | 0 | 0.000393±0.001304 | 0 | 0.001876±0.006221 | 0 | 0 | 0 | 0 |
| *Hyphomicrobiaceae* | 0.024408±0.037337 | 0.045200±0.068070 | 0.003947±0.007222 | 0.011563±0.022654 | 0.004316±0.012947 | 0 | 0.002711±0.006063 | 0 |
| *Idiomarinaceae* | 0.001695±0.005084 | 0 | 0.002270±0.007528 | 0.004640±0.010554 | 0.002129±0.004234 | 0.008171±0.016427 | 0 | 0.021353±0.035129 |
| *IncertaeSedisXI* | 0.001702±0.005105 | 0.000847±0.002810 | 0.014459±0.025006 | 0.021882±0.055379 | 0.002635±0.005300 | 0.018293±0.027435 | 0.015924±0.022506 | 0.006424±0.007446 |
| *IncertaeSedisXIII* | 0.004317±0.012950 | 0.001694±0.005619 | 0.016252±0.031837 | 0.012081±0.026924 | 0.002968±0.008903 | 0.003239±0.009718 | 0.007741±0.013836 | 0 |
| *IncertaeSedisXIV* | 0.004260±0.008707 | 0.006197±0.010797 | 0.008258±0.017123 | 0.001531±0.005079 | 0.003102±0.009306 | 0 | 0.012771±0.028558 | 0.019916±0.025181 |
| *Incertaesedis6* | 0.118655±0.128519 | 0.119339±0.097564 | 0.160174±0.113037 | 0.132315±0.102724 | 0.112275±0.072620 | 0.100089±0.068996 | 0.273617±0.366618 | 0.351813±0.442256 |
| *Intrasporangiaceae* | 0 | 0 | 0.000757±0.002509 | 0 | 0 | 0 | 0.001356±0.003032 | 0.006818±0.013635 |
| *Kineosporiaceae* | 0 | 0 | 0.000757±0.002509 | 0 | 0 | 0 | 0 | 0 |
| *Lachnospiraceae* | 0.048897±0.038621 | 0.053033±0.055547 | 0.215767±0.288437 | 0.167642±0.232946 | 0.199337±0.341319 | 0.082536±0.119585 | 0.135624±0.117123 | 0.174728±0.106253 |
| *Lactobacillaceae* | 1.722992±2.416351 | 0.711966±0.448118 | 2.276490±1.850826 | 0.760919±1.369297 | 1.204093±1.825931 | 0.770694±1.747806 | 3.509718±7.077021 | 1.023590±0.870122 |
| *Legionellaceae* | 0 | 0 | 0 | 0.003023±0.010027 | 0 | 0 | 0 | 0 |
| *Leptotrichiaceae* | 0 | 0 | 0 | 0 | 0.006204±0.018612 | 0 | 0 | 0 |
| *Leuconostocaceae* | 0.093866±0.093111 | 0.094842±0.075475 | 0.109848±0.080256 | 0.089529±0.051818 | 0.122628±0.137882 | 0.056960±0.045598 | 0.094003±0.073323 | 0.074070±0.085755 |
| *Listeriaceae* | 0.771534±0.252079 | 0.752608±0.376440 | 0.864884±0.246818 | 0.790422±0.191778 | 0.721397±0.153897 | 0.728404±0.281401 | 0.657336±0.253618 | 0.816769±0.174053 |
| *Malvaceae* | 0 | 0 | 0.020265±0.055481 | 0.031145±0.092674 | 0.033079±0.074088 | 0.017177±0.021746 | 0.001356±0.003032 | 0 |
| *Methylobacteriaceae* | 0.414798±0.519650 | 1.443108±1.452608 | 0.007577±0.008230 | 0.017368±0.021143 | 0.013453±0.027139 | 0.011089±0.017064 | 0.011809±0.016259 | 0.049554±0.085707 |
| *Methylocystaceae* | 0.007039±0.011325 | 0.017680±0.028203 | 0 | 0.002765±0.009170 | 0 | 0 | 0 | 0 |
| *Methylophilaceae* | 0 | 0.006025±0.019984 | 0 | 0 | 0 | 0 | 0 | 0 |
| *Microbacteriaceae* | 0.001885±0.005655 | 0.002001±0.006637 | 0.004474±0.008711 | 0.001783±0.004111 | 0.001003±0.003010 | 0.002743±0.008230 | 0.010216±0.014040 | 0.009437±0.012874 |
| *Micrococcaceae* | 0.004680±0.007236 | 0.004523±0.010144 | 0.005625±0.006729 | 0.006019±0.010930 | 0.007185±0.010741 | 0.007164±0.011372 | 0.006125±0.010332 | 0.002620±0.005239 |
| *Mogibacteriaceae* | 0.002158±0.006475 | 0.001848±0.004128 | 0.007871±0.013742 | 0.012476±0.020288 | 0.004316±0.012947 | 0.005714±0.011579 | 0.002711±0.006063 | 0.008255±0.010200 |
| *Moraxellaceae* | 1.527438±1.021336 | 1.543988±0.821492 | 1.407010±0.807753 | 1.329406±1.013235 | 2.326536±2.243446 | 1.976624±1.284962 | 0.886071±0.536962 | 1.304155±0.599195 |
| *Mycobacteriaceae* | 0 | 0 | 0 | 0 | 0.003102±0.009306 | 0 | 0 | 0 |
| *Mycoplasmataceae* | 0 | 0 | 0 | 0.001876±0.006221 | 0 | 0 | 0 | 0 |
| *Neisseriaceae* | 0.009435±0.012628 | 0.002517±0.004388 | 0.007555±0.012930 | 0.007302±0.010627 | 0.012303±0.016583 | 0.022284±0.037923 | 0 | 0.027531±0.046420 |
| *Nocardiaceae* | 0 | 0 | 0.001679±0.003757 | 0.001531±0.005079 | 0 | 0 | 0 | 0 |
| *Nocardioidaceae* | 0 | 0 | 0 | 0 | 0.001126±0.003377 | 0 | 0 | 0 |
| *Oscillospiraceae* | 0.010498±0.008265 | 0.024328±0.038635 | 0.031197±0.033381 | 0.032408±0.038676 | 0.082043±0.201949 | 0.029216±0.031688 | 0.023291±0.028115 | 0.021890±0.023400 |
| *Oxalobacteraceae* | 0.183807±0.186484 | 0.235658±0.123743 | 0.144158±0.128834 | 0.089762±0.109255 | 0.065846±0.042783 | 0.041940±0.048239 | 0.052547±0.056415 | 0.092955±0.048669 |
| *Paenibacillaceae* | 0.104078±0.092236 | 0.101314±0.075059 | 0.213640±0.235904 | 0.139916±0.162400 | 0.090899±0.150349 | 0.051898±0.047804 | 0.203311±0.242350 | 0.241682±0.178364 |
| *Paraprevotellaceae* | 0.003722±0.008088 | 0.002695±0.006234 | 0.025504±0.039531 | 0.013032±0.020673 | 0.005384±0.011119 | 0.008726±0.018039 | 0 | 0.021376±0.008049 |
| *Pasteurellaceae* | 0.002823±0.005745 | 0.005969±0.019797 | 0.005102±0.008861 | 0.001876±0.006221 | 0.025260±0.064678 | 0.004672±0.014017 | 0.004769±0.010664 | 0.009044±0.006165 |
| *Pelobacteraceae* | 0.001702±0.005105 | 0 | 0 | 0 | 0 | 0.015205±0.031638 | 0 | 0 |
| *Peptococcaceae* | 0 | 0.007783±0.017122 | 0.005096±0.009359 | 0.005609±0.011266 | 0.003102±0.009306 | 0 | 0 | 0 |
| *Peptostreptococcaceae* | 0.010083±0.020008 | 0.009763±0.011853 | 0.046473±0.111670 | 0.040295±0.068088 | 0.018833±0.015590 | 0.018303±0.027439 | 0.024082±0.018164 | 0.040402±0.026671 |
| *Phormidiaceae* | 0 | 0 | 0 | 0 | 0 | 0.002336±0.007009 | 0 | 0 |
| *Phycisphaeraceae* | 0 | 0.001001±0.003318 | 0 | 0 | 0 | 0 | 0 | 0 |
| *Phyllobacteriaceae* | 0.005344±0.011081 | 0.011984±0.027743 | 0.000757±0.002509 | 0 | 0.004316±0.012947 | 0 | 0.012771±0.028558 | 0.006818±0.013635 |
| *Pinaceae* | 0 | 0.001001±0.003318 | 0.001263±0.004188 | 0 | 0.001126±0.003377 | 0 | 0 | 0 |
| *Pirellulaceae* | 0 | 0 | 0.000757±0.002509 | 0 | 0 | 0 | 0 | 0 |
| *Planctomycetaceae* | 0 | 0.002001±0.006637 | 0.000922±0.003058 | 0 | 0 | 0 | 0 | 0.003409±0.006818 |
| *Planococcaceae* | 0.580060±0.303902 | 0.758111±0.375897 | 0.977298±0.491183 | 0.726432±0.327031 | 1.038682±0.801203 | 0.916014±0.574953 | 0.651059±0.260332 | 1.304026±0.593783 |
| *Polyangiaceae* | 0 | 0 | 0.029336±0.094568 | 0 | 0 | 0 | 0 | 0 |
| *Porphyromonadaceae* | 0.019683±0.037993 | 0.010030±0.013091 | 0.014946±0.024286 | 0.006298±0.018572 | 0.010701±0.020143 | 0.014987±0.020124 | 0.005423±0.012126 | 0.016905±0.019616 |
| *Prevotellaceae* | 0.001958±0.005874 | 0.014912±0.031173 | 0.051695±0.102755 | 0.018621±0.020857 | 0.007207±0.018478 | 0.015981±0.021138 | 0.011809±0.016259 | 0.017296±0.020848 |
| *Prolixibacteraceae* | 0.001885±0.005655 | 0 | 0 | 0 | 0 | 0 | 0 | 0 |
| *Propionibacteriaceae* | 0.026109±0.037999 | 0.009957±0.017287 | 0.029073±0.027870 | 0.036778±0.057586 | 0.063240±0.126599 | 0.023087±0.025185 | 0.033350±0.053914 | 0.025553±0.023299 |
| *Pseudanabaenaceae* | 0 | 0 | 0 | 0 | 0 | 0 | 0.002711±0.006063 | 0 |
| *Pseudomonadaceae* | 4.433029±2.539996 | 6.353179±2.833828 | 3.811297±1.662659 | 3.566672±1.775722 | 3.875012±1.647736 | 3.089011±1.633588 | 3.129151±1.943075 | 4.321440±1.864868 |
| *Puniceicoccaceae* | 0.001129±0.003386 | 0 | 0 | 0 | 0 | 0 | 0 | 0 |
| *RB40* | 0 | 0.002522±0.008364 | 0 | 0 | 0 | 0 | 0 | 0 |
| *RF16* | 0 | 0.003369±0.008553 | 0.012308±0.016889 | 0.010388±0.019884 | 0.010639±0.031918 | 0.002336±0.007009 | 0.003740±0.005435 | 0.005635±0.006539 |
| *Rhizobiaceae* | 0.012247±0.023474 | 0.045713±0.048486 | 0.007483±0.012763 | 0.006538±0.009847 | 0.006479±0.012871 | 0.009752±0.021600 | 0.004067±0.009095 | 0 |
| *Rhodobacteraceae* | 0.043062±0.076797 | 0.040656±0.049713 | 0.066807±0.049981 | 0.068813±0.095228 | 0.034374±0.051714 | 0.041904±0.040389 | 0.069308±0.061427 | 0.061624±0.038558 |
| *Rhodocyclaceae* | 0.001885±0.005655 | 0.003833±0.008989 | 0.007990±0.019317 | 0.008952±0.016231 | 0.003284±0.006917 | 0 | 0 | 0 |
| *Rhodospirillaceae* | 0.003287±0.006922 | 0.003369±0.008553 | 0.011438±0.022591 | 0.001783±0.004111 | 0.002251±0.006754 | 0.006479±0.019436 | 0 | 0.006424±0.007446 |
| *Rhodothermaceae* | 0 | 0 | 0.000757±0.002509 | 0 | 0 | 0 | 0 | 0 |
| *Rikenellaceae* | 0.009498±0.023402 | 0.011564±0.020270 | 0.027823±0.040950 | 0.016403±0.028330 | 0.021214±0.045867 | 0.007912±0.016027 | 0.031995±0.054195 | 0.019795±0.029730 |
| *Rivulariaceae* | 0 | 0 | 0.002766±0.009173 | 0 | 0 | 0 | 0 | 0 |
| *Rubrobacteraceae* | 0 | 0 | 0.000922±0.003058 | 0 | 0 | 0 | 0 | 0 |
| *Ruminococcaceae* | 0.097041±0.078641 | 0.151192±0.167124 | 0.378230±0.373160 | 0.313016±0.397910 | 0.331388±0.675038 | 0.097376±0.093211 | 0.226343±0.170833 | 0.282104±0.294489 |
| *S24-7* | 0 | 0.005889±0.013251 | 0.006625±0.011735 | 0.009711±0.019662 | 0.007093±0.021279 | 0 | 0.007741±0.013836 | 0.005239±0.010479 |
| *Shewanellaceae* | 0.002594±0.007781 | 0.002770±0.009187 | 0.000631±0.002094 | 0 | 0 | 0.002336±0.007009 | 0.001356±0.003032 | 0 |
| *Sphingobacteriaceae* | 0.009260±0.019208 | 0.015980±0.019252 | 0.017884±0.032941 | 0.019657±0.029633 | 0.014968±0.017890 | 0 | 0.001356±0.003032 | 0.024118±0.019864 |
| *Sphingomonadaceae* | 0.029235±0.031460 | 0.048798±0.046647 | 0.046951±0.042542 | 0.027437±0.033401 | 0.027383±0.022987 | 0.033245±0.039154 | 0.018327±0.029515 | 0.019149±0.006520 |
| *Spirochaetaceae* | 0 | 0.002739±0.006614 | 0.008408±0.025250 | 0.011931±0.031407 | 0.003161±0.006790 | 0 | 0.020930±0.026519 | 0.004077±0.008154 |
| *Spiroplasmataceae* | 0 | 0.001892±0.006275 | 0 | 0 | 0 | 0 | 0 | 0 |
| *Staphylococcaceae* | 0.067246±0.065617 | 0.890294±2.538393 | 0.371279±0.955131 | 1.127536±2.215954 | 1.089652±2.394181 | 2.646913±2.810164 | 1.328786±1.641316 | 1.202256±1.730311 |
| *Streptococcaceae* | 3.517345±1.618629 | 4.111579±2.233830 | 4.375381±2.075258 | 5.679102±7.454299 | 4.347447±2.501747 | 3.599489±2.168216 | 3.185598±1.622820 | 4.301504±1.648892 |
| *Succinivibrionaceae* | 0.004525±0.006947 | 0.015484±0.023468 | 0.013557±0.025565 | 0.005099±0.009913 | 0.010254±0.021176 | 0.005080±0.010121 | 0.008158±0.012172 | 0.014556±0.019794 |
| *Sutterellaceae* | 0.004116±0.008180 | 0.003389±0.011239 | 0.001093±0.003625 | 0.004846±0.010397 | 0.012408±0.037223 | 0 | 0 | 0 |
| *Syntrophobacteraceae* | 0 | 0 | 0 | 0 | 0 | 0 | 0.004067±0.009095 | 0 |
| *Thermaceae* | 0 | 0.000669±0.002220 | 0 | 0 | 0 | 0 | 0 | 0 |
| *Thermoactinomycetaceae* | 0 | 0 | 0 | 0.002910±0.009651 | 0 | 0 | 0 | 0 |
| *Tissierellaceae* | 0 | 0.003369±0.008553 | 0.002083±0.004841 | 0.007339±0.012832 | 0.002158±0.006474 | 0.006284±0.012476 | 0 | 0 |
| *Tremblayaceae* | 0 | 0.011006±0.036503 | 0 | 0 | 0 | 0 | 0 | 0 |
| *Trueperaceae* | 0 | 0 | 0 | 0.000671±0.002225 | 0 | 0 | 0 | 0 |
| *Veillonellaceae* | 0.007157±0.015574 | 0.023159±0.027221 | 0.050342±0.047171 | 0.034721±0.047671 | 0.047484±0.092539 | 0.017611±0.024964 | 0.040087±0.067780 | 0.028446±0.031579 |
| *Verrucomicrobiaceae* | 0 | 0 | 0.004667±0.009268 | 0.002765±0.009170 | 0.015510±0.046529 | 0 | 0.001356±0.003032 | 0.002620±0.005239 |
| *Vibrionaceae* | 0 | 0.000393±0.001304 | 0.000631±0.002094 | 0 | 0.006204±0.018612 | 0.003539±0.010616 | 0.006802±0.011792 | 0 |
| *Victivallaceae* | 0 | 0.002561±0.006443 | 0.001326±0.004398 | 0 | 0 | 0 | 0.004769±0.010664 | 0 |
| *Weeksellaceae* | 0.002594±0.007781 | 0 | 0 | 0.003023±0.010027 | 0 | 0 | 0 | 0 |
| *Xanthobacteraceae* | 0 | 0.005274±0.009123 | 0.001093±0.003625 | 0 | 0 | 0.003239±0.009718 | 0.006779±0.015158 | 0.009712±0.006927 |
| *Xanthomonadaceae* | 0.134619±0.123676 | 0.164056±0.120032 | 0.249193±0.286437 | 0.223668±0.303473 | 0.147004±0.105620 | 0.088478±0.067657 | 0.395710±0.450353 | 0.767749±0.534160 |
| *Xenococcaceae* | 0 | 0 | 0.015043±0.024815 | 0.000671±0.002225 | 0 | 0.001589±0.004766 | 0.006779±0.015158 | 0 |
| *Yaniellaceae* | 0 | 0.003002±0.009955 | 0.002459±0.008156 | 0 | 0 | 0 | 0 | 0 |
| *mb2424* | 0 | 0 | 0 | 0.001876±0.006221 | 0 | 0 | 0 | 0 |
| *p-2534-18B5* | 0.001958±0.005874 | 0.001892±0.006275 | 0.012329±0.025331 | 0.002546±0.006394 | 0.002158±0.006474 | 0.003044±0.009132 | 0 | 0.006424±0.007446 |
| *unclassified* | 0.472808±0.230136 | 0.599324±0.439506 | 1.142217±0.776188 | 0.706902±0.440793 | 1.267225±1.227673 | 0.521089±0.175835 | 0.596347±0.361667 | 0.795962±0.401063 |
| Genus |  |  |  |  |  |  |  |  |
| *Acetobacter* | 0 | 0 | 0.002046±0.004578 | 0 | 0 | 0 | 0.007741±0.013836 | 0 |
| *Acholeplasma* | 0.001702±0.005105 | 0.005350±0.010064 | 0.002015±0.004503 | 0 | 0.004013±0.012040 | 0 | 0.019223±0.026390 | 0.005239±0.010479 |
| *Achromobacter* | 0 | 0 | 0.002185±0.004932 | 0.001342±0.004450 | 0.003546±0.010639 | 0.002336±0.007009 | 0 | 0.004077±0.008154 |
| *Acidovorax* | 0.190245±0.275011 | 0.624340±0.456006 | 0.025883±0.026773 | 0.027612±0.048843 | 0.013054±0.024508 | 0.008367±0.013410 | 0.016905±0.018621 | 0.030165±0.034519 |
| *Acinetobacter* | 0.722877±0.714438 | 0.849880±0.606562 | 0.506301±0.344840 | 0.443492±0.424739 | 1.412111±2.127555 | 1.170163±1.139383 | 0.289542±0.370699 | 0.301354±0.180961 |
| *Actinomyces* | 0 | 0 | 0.001124±0.003727 | 0 | 0 | 0 | 0 | 0 |
| *Adhaeribacter* | 0 | 0.001001±0.003318 | 0 | 0 | 0.001126±0.003377 | 0 | 0 | 0 |
| *Aerococcus* | 0.010325±0.019951 | 0 | 0.005620±0.010334 | 0.010863±0.025367 | 0.060474±0.072701 | 0.013291±0.022397 | 0.038654±0.045840 | 0.080360±0.097087 |
| *Aeromonas* | 0.010576±0.022982 | 0.013687±0.018186 | 0.037034±0.047109 | 0.036343±0.050319 | 0.051809±0.148282 | 0.025214±0.047875 | 0.011548±0.016207 | 0.006029±0.007079 |
| *Afipia* | 0 | 0.000669±0.002220 | 0.002651±0.006527 | 0 | 0 | 0 | 0 | 0 |
| *Agromyces* | 0 | 0 | 0.000922±0.003058 | 0 | 0 | 0 | 0 | 0 |
| *Akkermansia* | 0 | 0 | 0.003745±0.009173 | 0.002765±0.009170 | 0.015510±0.046529 | 0 | 0.001356±0.003032 | 0.002620±0.005239 |
| *Albidiferax* | 0 | 0.002494±0.008272 | 0 | 0.002910±0.009651 | 0.002251±0.006754 | 0 | 0 | 0 |
| *Alcaligenes* | 0 | 0 | 0.002186±0.007250 | 0 | 0 | 0 | 0 | 0 |
| *Alcanivorax* | 0 | 0.002678±0.008882 | 0 | 0 | 0 | 0 | 0 | 0 |
| *Aliidiomarina* | 0.001695±0.005084 | 0 | 0.002270±0.007528 | 0.004640±0.010554 | 0.002129±0.004234 | 0.008171±0.016427 | 0 | 0.021353±0.035129 |
| *Alishewanella* | 0 | 0 | 0 | 0 | 0.002158±0.006474 | 0 | 0 | 0 |
| *Alistipes* | 0.009498±0.023402 | 0.011564±0.020270 | 0.027823±0.040950 | 0.016403±0.028330 | 0.021214±0.045867 | 0.007912±0.016027 | 0.031995±0.054195 | 0.019795±0.029730 |
| *Alkalibacterium* | 0.001666±0.004999 | 0.000847±0.002810 | 0.004841±0.013526 | 0.008947±0.014297 | 0.014962±0.020029 | 0.021859±0.030155 | 0 | 0.002620±0.005239 |
| *Alkalibaculum* | 0 | 0 | 0 | 0.005463±0.012186 | 0.002968±0.008903 | 0 | 0 | 0 |
| *Alkanindiges* | 0.002158±0.006475 | 0.000669±0.002220 | 0.001124±0.003727 | 0.001112±0.003687 | 0.002158±0.006474 | 0.005586±0.011273 | 0 | 0.006031±0.012061 |
| *Allobaculum* | 0 | 0.001001±0.003318 | 0.001326±0.004398 | 0 | 0.009306±0.027917 | 0 | 0.004067±0.009095 | 0 |
| *Alloiococcus* | 0 | 0.000393±0.001304 | 0 | 0 | 0 | 0 | 0 | 0 |
| *Alloprevotella* | 0 | 0.005083±0.016858 | 0.002802±0.004884 | 0.005570±0.011157 | 0 | 0.005993±0.011947 | 0 | 0 |
| *Altererythrobacter* | 0 | 0 | 0 | 0 | 0.001126±0.003377 | 0 | 0 | 0 |
| *Amaricoccus* | 0 | 0 | 0 | 0.001531±0.005079 | 0 | 0 | 0 | 0.003409±0.006818 |
| *Aminobacter* | 0.004215±0.008392 | 0.011984±0.027743 | 0.000757±0.002509 | 0 | 0 | 0 | 0 | 0 |
| *Amphibacillus* | 0 | 0 | 0.001093±0.003625 | 0 | 0 | 0 | 0 | 0 |
| *Anaerococcus* | 0 | 0 | 0.012445±0.025125 | 0.015946±0.040653 | 0.002635±0.005300 | 0.009328±0.019588 | 0.007154±0.015997 | 0 |
| *Anaerofilum* | 0.001129±0.003386 | 0 | 0.005633±0.015130 | 0.000671±0.002225 | 0.003546±0.010639 | 0 | 0.002385±0.005332 | 0.003409±0.006818 |
| *Anaerofustis* | 0 | 0 | 0.002459±0.008156 | 0 | 0.003102±0.009306 | 0 | 0 | 0 |
| *Anaeroplasma* | 0 | 0.000847±0.002810 | 0.003090±0.008215 | 0.001876±0.006221 | 0.003102±0.009306 | 0.002743±0.008230 | 0 | 0.003409±0.006818 |
| *Anaerostipes* | 0 | 0 | 0.011983±0.016888 | 0.005828±0.012987 | 0 | 0 | 0.002711±0.006063 | 0.010874±0.014840 |
| *Anaerovibrio* | 0.001695±0.005084 | 0.003833±0.008989 | 0.002415±0.006073 | 0.003751±0.012441 | 0 | 0.002336±0.007009 | 0 | 0.006697±0.008091 |
| *Anaerovorax* | 0 | 0 | 0.006964±0.016275 | 0.001876±0.006221 | 0 | 0.003239±0.009718 | 0 | 0 |
| *Anoxybacillus* | 0.104125±0.106959 | 0.210332±0.370710 | 0.184311±0.423699 | 0.043649±0.069242 | 0.618819±1.624885 | 0.124244±0.121390 | 2.258321±4.336155 | 0.467131±0.331398 |
| *Aquabacterium* | 0.002594±0.007781 | 0.004296±0.007313 | 0.058040±0.131009 | 0.009411±0.014037 | 0.008351±0.012921 | 0.006969±0.010955 | 0.011548±0.016207 | 0.025299±0.029847 |
| *Aquamicrobium* | 0 | 0 | 0 | 0 | 0.004316±0.012947 | 0 | 0 | 0.006818±0.013635 |
| *Aquincola* | 0.002158±0.006475 | 0 | 0 | 0 | 0 | 0 | 0 | 0 |
| *Aquitalea* | 0.001695±0.005084 | 0 | 0 | 0 | 0 | 0 | 0 | 0 |
| *Arcobacter* | 0 | 0.005016±0.011160 | 0.006556±0.010411 | 0.003224±0.008537 | 0.001126±0.003377 | 0 | 0.003740±0.005435 | 0.012057±0.014159 |
| *Armatimonas* | 0 | 0 | 0 | 0 | 0.003102±0.009306 | 0 | 0.004067±0.009095 | 0 |
| *Arthrobacter* | 0.104240±0.091059 | 0.114561±0.106148 | 0.131240±0.082906 | 0.096695±0.056982 | 0.119276±0.082624 | 0.111282±0.080989 | 0.112588±0.098052 | 0.150475±0.117549 |
| *Atopobium* | 0 | 0 | 0 | 0 | 0 | 0.003239±0.009718 | 0 | 0 |
| *Atopostipes* | 0.017469±0.031220 | 0.022437±0.027705 | 0.074003±0.084711 | 0.056862±0.089720 | 0.091241±0.169074 | 0.089126±0.158995 | 0.020996±0.014560 | 0.055747±0.039259 |
| *Aurantimonas* | 0 | 0 | 0 | 0.001112±0.003687 | 0 | 0 | 0 | 0 |
| *Azonexus* | 0 | 0 | 0 | 0.000671±0.002225 | 0 | 0 | 0 | 0 |
| *Azospira* | 0 | 0 | 0 | 0.008281±0.014706 | 0 | 0 | 0 | 0 |
| *Azospirillum* | 0.002158±0.006475 | 0.003369±0.008553 | 0.011438±0.022591 | 0.001783±0.004111 | 0 | 0 | 0 | 0.003015±0.006031 |
| *BD2-13* | 0.002823±0.005745 | 0.001001±0.003318 | 0.001093±0.003625 | 0 | 0.002968±0.008903 | 0 | 0.006386±0.014279 | 0 |
| *Bacillus* | 79.646269±9.477887 | 72.601854±6.546227 | 75.607923±7.729766 | 77.399274±12.502635 | 75.509401±9.229390 | 79.222954±7.135204 | 74.551076±11.940780 | 74.596198±6.129435 |
| *Bacteroides* | 0.011675±0.029206 | 0.025829±0.033639 | 0.089396±0.104042 | 0.049553±0.080089 | 0.053922±0.108375 | 0.038555±0.050097 | 0.101236±0.126811 | 0.052043±0.056793 |
| *Barnesiella* | 0.002257±0.006772 | 0.008438±0.014728 | 0.026600±0.038043 | 0.010005±0.018934 | 0.013216±0.016377 | 0.002743±0.008230 | 0.009490±0.021221 | 0.015345±0.012887 |
| *Bdellovibrio* | 0 | 0 | 0 | 0 | 0 | 0 | 0.001356±0.003032 | 0 |
| *Bhargavaea* | 0 | 0 | 0.001124±0.003727 | 0 | 0 | 0.003044±0.009132 | 0 | 0 |
| *Bifidobacterium* | 0 | 0 | 0 | 0 | 0 | 0 | 0 | 0.003409±0.006818 |
| *Blastocatella* | 0.017745±0.035049 | 0.001670±0.003804 | 0.006971±0.011097 | 0.003751±0.012441 | 0.004503±0.013508 | 0.006355±0.019064 | 0 | 0 |
| *Blautia* | 0.004260±0.008707 | 0.006197±0.010797 | 0.008258±0.017123 | 0.001531±0.005079 | 0.003102±0.009306 | 0 | 0.012771±0.028558 | 0.014677±0.017032 |
| *Bordetella* | 0 | 0 | 0 | 0 | 0.003546±0.010639 | 0 | 0 | 0 |
| *Bosea* | 0.025404±0.033518 | 0.063897±0.068751 | 0.000757±0.002509 | 0 | 0 | 0 | 0 | 0.002620±0.005239 |
| *Brachybacterium* | 0 | 0 | 0.004080±0.009106 | 0 | 0 | 0 | 0 | 0.002620±0.005239 |
| *Bradyrhizobium* | 0.003287±0.006922 | 0 | 0.003528±0.006225 | 0.003665±0.008890 | 0 | 0 | 0 | 0.006697±0.008091 |
| *Brevibacillus* | 0.083896±0.081112 | 0.061739±0.062437 | 0.142377±0.215058 | 0.089307±0.124597 | 0.043011±0.069177 | 0.011329±0.021285 | 0.120213±0.204245 | 0.088856±0.067748 |
| *Brevibacterium* | 0 | 0.001001±0.003318 | 0 | 0 | 0 | 0 | 0 | 0 |
| *Brevundimonas* | 0.022235±0.028199 | 0.051913±0.066594 | 0.014547±0.016200 | 0.022742±0.028438 | 0.009882±0.015702 | 0.010404±0.019597 | 0.012577±0.017491 | 0.018088±0.012331 |
| *Brochothrix* | 0.749191±0.256144 | 0.737908±0.383177 | 0.841199±0.251392 | 0.759772±0.191461 | 0.701228±0.159327 | 0.708945±0.290563 | 0.643305±0.272203 | 0.802486±0.161569 |
| *Brucella* | 0.001695±0.005084 | 0.001694±0.005619 | 0.003713±0.006393 | 0 | 0.006474±0.019421 | 0 | 0 | 0 |
| *Bryobacter* | 0 | 0 | 0 | 0.000671±0.002225 | 0.003102±0.009306 | 0 | 0.019157±0.042837 | 0 |
| *Budvicia* | 0 | 0 | 0 | 0 | 0.001510±0.004529 | 0 | 0 | 0 |
| *Bulleidia* | 0 | 0 | 0.002186±0.007250 | 0.001876±0.006221 | 0 | 0.003250±0.009749 | 0 | 0 |
| *Burkholderia* | 0 | 0.002532±0.005645 | 0 | 0.000671±0.002225 | 0 | 0 | 0 | 0 |
| *Buttiauxella* | 0 | 0 | 0.000922±0.003058 | 0.000671±0.002225 | 0 | 0.014946±0.025759 | 0.002385±0.005332 | 0 |
| *Butyricicoccus* | 0 | 0.000669±0.002220 | 0.002652±0.008797 | 0.001876±0.006221 | 0.003102±0.009306 | 0 | 0 | 0 |
| *Butyrivibrio* | 0.008405±0.012077 | 0.007758±0.015490 | 0.019872±0.023464 | 0.018555±0.042848 | 0.015939±0.016047 | 0.026568±0.041723 | 0.018284±0.012701 | 0.018633±0.021848 |
| *Byssovorax* | 0 | 0 | 0.001844±0.006115 | 0 | 0 | 0 | 0 | 0 |
| *CF231* | 0.002594±0.007781 | 0.002695±0.006234 | 0.017613±0.024111 | 0.005960±0.010523 | 0.003377±0.010131 | 0.003239±0.009718 | 0 | 0.010874±0.014840 |
| *Calothrix* | 0 | 0 | 0.002766±0.009173 | 0 | 0 | 0 | 0 | 0 |
| *Campylobacter* | 0.003652±0.007272 | 0 | 0.002270±0.007528 | 0 | 0.009885±0.021293 | 0 | 0.006386±0.014279 | 0.004077±0.008154 |
| *Candidatus Hamiltonella* | 0 | 0 | 0 | 0.000671±0.002225 | 0 | 0 | 0 | 0 |
| *Candidatus Tremblaya* | 0 | 0.011006±0.036503 | 0 | 0 | 0 | 0 | 0 | 0 |
| *Capnocytophaga* | 0 | 0 | 0 | 0.003751±0.012441 | 0 | 0 | 0 | 0 |
| *Carnobacterium* | 0.078795±0.078510 | 0.041808±0.040925 | 0.043397±0.034879 | 0.076050±0.029487 | 0.047999±0.060846 | 0.044026±0.033043 | 0.073413±0.037910 | 0.032272±0.011666 |
| *Caryophanon* | 0.005084±0.015251 | 0.000669±0.002220 | 0.009322±0.018750 | 0.009369±0.014644 | 0.041360±0.094362 | 0.009523±0.020416 | 0.006386±0.014279 | 0.017967±0.013477 |
| *Caulobacter* | 0.007356±0.011709 | 0.005734±0.008824 | 0.007430±0.016092 | 0.011884±0.021009 | 0.004093±0.009119 | 0.001589±0.004766 | 0.006386±0.014279 | 0.012061±0.024123 |
| *Cellulomonas* | 0 | 0 | 0 | 0 | 0 | 0 | 0 | 0.003015±0.006031 |
| *Cellulosilyticum* | 0 | 0 | 0.004390±0.007710 | 0.010358±0.018886 | 0.010935±0.012665 | 0.006088±0.018265 | 0.007481±0.010870 | 0.013099±0.026197 |
| *Cellvibrio* | 0 | 0.070851±0.211029 | 0.007518±0.016690 | 0.009367±0.016313 | 0.017668±0.046169 | 0 | 0.006386±0.014279 | 0.009046±0.018092 |
| *Chromohalobacter* | 0.001666±0.004999 | 0.000847±0.002810 | 0.000757±0.002509 | 0 | 0 | 0 | 0 | 0.003015±0.006031 |
| *Chryseobacterium* | 0.016250±0.022068 | 0.032080±0.027492 | 0.028991±0.036678 | 0.019220±0.032640 | 0.021186±0.033442 | 0.014050±0.028912 | 0.021020±0.025239 | 0.036177±0.029796 |
| *Chryseomicrobium* | 0 | 0.001517±0.003403 | 0.001357±0.004502 | 0.007490±0.019063 | 0 | 0.003239±0.009718 | 0 | 0 |
| *Citrobacter* | 0 | 0 | 0 | 0 | 0.002888±0.008663 | 0.039204±0.106193 | 0.012771±0.028558 | 0.003015±0.006031 |
| *Clostridium* | 0.081443±0.056929 | 0.104945±0.070284 | 0.358963±0.524243 | 0.241430±0.312820 | 0.340576±0.579579 | 0.184513±0.219149 | 0.143042±0.080943 | 0.284708±0.150203 |
| *Comamonas* | 0.011144±0.014426 | 0.017000±0.016187 | 0.021389±0.024991 | 0.014018±0.017886 | 0.032247±0.044290 | 0.018967±0.020405 | 0.003740±0.005435 | 0.012059±0.009932 |
| *Coprobacillus* | 0 | 0 | 0.001783±0.005914 | 0 | 0.006204±0.018612 | 0 | 0 | 0 |
| *Coprococcus* | 0.006509±0.013255 | 0.005063±0.011265 | 0.017677±0.031423 | 0.033811±0.043212 | 0.070439±0.173769 | 0.008124±0.012290 | 0.028696±0.042814 | 0.006818±0.013635 |
| *Corynebacterium* | 0.006882±0.015755 | 0.013510±0.020274 | 0.050288±0.081058 | 0.039885±0.055252 | 0.116554±0.180687 | 0.045304±0.053743 | 0.020760±0.036141 | 0.080263±0.024169 |
| *Cupriavidus* | 0.149815±0.148603 | 0.112170±0.152543 | 0.197901±0.215086 | 0.204058±0.306353 | 0.077196±0.064293 | 0.080110±0.099461 | 0.305744±0.439816 | 0.417416±0.336718 |
| *Curtobacterium* | 0 | 0 | 0 | 0 | 0 | 0 | 0.005447±0.012179 | 0 |
| *Cystobacter* | 0 | 0 | 0.001513±0.005019 | 0 | 0 | 0 | 0 | 0 |
| *Dehalobacterium* | 0 | 0.000669±0.002220 | 0 | 0 | 0 | 0 | 0 | 0 |
| *Deinococcus* | 0.035486±0.044573 | 0.039988±0.060772 | 0.046539±0.057500 | 0.065193±0.096898 | 0.047282±0.048411 | 0.039230±0.039482 | 0.111265±0.071115 | 0.110631±0.064486 |
| *Delftia* | 0.010852±0.010449 | 0.007300±0.014637 | 0.010731±0.019918 | 0.004405±0.010675 | 0.003516±0.007067 | 0.003539±0.010616 | 0.005096±0.007002 | 0.003409±0.006818 |
| *Desemzia* | 0.001695±0.005084 | 0.008364±0.022314 | 0.014159±0.033331 | 0.017770±0.023430 | 0.012796±0.032175 | 0.009587±0.019896 | 0 | 0.022732±0.028980 |
| *Desulfomicrobium* | 0 | 0 | 0.000757±0.002509 | 0 | 0 | 0 | 0.002711±0.006063 | 0 |
| *Desulfonispora* | 0 | 0 | 0 | 0.003063±0.010159 | 0 | 0 | 0 | 0 |
| *Desulfovibrio* | 0 | 0.002394±0.006635 | 0.001513±0.005019 | 0 | 0 | 0 | 0 | 0 |
| *Desulfurispora* | 0 | 0 | 0 | 0.000671±0.002225 | 0 | 0 | 0 | 0 |
| *Devosia* | 0 | 0 | 0.002046±0.004578 | 0.007158±0.018844 | 0.004316±0.012947 | 0 | 0.001356±0.003032 | 0 |
| *Dietzia* | 0 | 0 | 0.002459±0.008156 | 0.001876±0.006221 | 0.004409±0.008752 | 0.006997±0.020991 | 0 | 0.002620±0.005239 |
| *Dokdonella* | 0 | 0 | 0 | 0 | 0.001126±0.003377 | 0 | 0 | 0 |
| *Dolosigranulum* | 0 | 0.005356±0.017763 | 0 | 0 | 0 | 0 | 0 | 0 |
| *Dorea* | 0 | 0.005597±0.010863 | 0.022269±0.048592 | 0.012703±0.016209 | 0.003546±0.010639 | 0 | 0.014544±0.014893 | 0.017967±0.013477 |
| *Duganella* | 0 | 0 | 0 | 0 | 0.001510±0.004529 | 0 | 0 | 0 |
| *Dysgonomonas* | 0.001129±0.003386 | 0 | 0 | 0 | 0 | 0 | 0 | 0 |
| *Empedobacter* | 0.002831±0.005763 | 0.000847±0.002810 | 0.000757±0.002509 | 0.000671±0.002225 | 0.003392±0.010175 | 0 | 0 | 0 |
| *Enhydrobacter* | 0.118655±0.128519 | 0.119339±0.097564 | 0.160174±0.113037 | 0.132315±0.102724 | 0.112275±0.072620 | 0.100089±0.068996 | 0.273617±0.366618 | 0.351813±0.442256 |
| *Enterobacter* | 0.007638±0.013062 | 0.016723±0.028336 | 0.014783±0.016920 | 0.399680±1.265296 | 0.003102±0.009306 | 0.011110±0.013997 | 0.079079±0.152533 | 0.025430±0.032686 |
| *Enterococcus* | 0.013029±0.013329 | 0.020052±0.043266 | 0.026642±0.020702 | 0.008500±0.019991 | 0.022729±0.023812 | 0.007164±0.011372 | 0.011145±0.015271 | 0.020584±0.022377 |
| *Epilithonimonas* | 0 | 0.001001±0.003318 | 0.001513±0.005019 | 0.000671±0.002225 | 0 | 0 | 0 | 0 |
| *Erwinia* | 0 | 0 | 0 | 0 | 0.001126±0.003377 | 0 | 0 | 0 |
| *Erysipelothrix* | 0 | 0.000847±0.002810 | 0 | 0.002553±0.008467 | 0 | 0 | 0 | 0 |
| *Escherichia* | 0 | 0.049085±0.093310 | 0.123840±0.234974 | 0.010720±0.030976 | 0.139588±0.166287 | 0.026082±0.039185 | 0.062364±0.139451 | 0.011268±0.014903 |
| *Escherichia/Shigella* | 0 | 0.020291±0.036068 | 0.138410±0.297307 | 0.009378±0.031103 | 0.040012±0.053392 | 0 | 0.044740±0.100041 | 0.002620±0.005239 |
| *Eubacterium* | 0.018386±0.039982 | 0.026652±0.036588 | 0.088507±0.093420 | 0.047866±0.057313 | 0.074192±0.145348 | 0.073099±0.102860 | 0.079586±0.117860 | 0.081419±0.058526 |
| *Ewingella* | 0 | 0 | 0.001513±0.005019 | 0 | 0 | 0 | 0 | 0 |
| *Exiguobacterium* | 0.003087±0.006403 | 0.007357±0.011105 | 0.007538±0.014076 | 0.007776±0.014103 | 0.004105±0.009416 | 0 | 0.001356±0.003032 | 0.005635±0.006539 |
| *Facklamia* | 0.015954±0.015324 | 0.012731±0.019460 | 0.037282±0.047946 | 0.060523±0.128728 | 0.045341±0.080728 | 0.075859±0.139471 | 0.014694±0.014885 | 0.041839±0.040281 |
| *Faecalibacterium* | 0.013449±0.034973 | 0.004380±0.006214 | 0.002185±0.004932 | 0.006448±0.017073 | 0.002251±0.006754 | 0.004828±0.010275 | 0.025543±0.057115 | 0.016507±0.024773 |
| *Fibrobacter* | 0 | 0 | 0.002459±0.008156 | 0 | 0 | 0 | 0 | 0 |
| *Fimbriimonas* | 0 | 0 | 0 | 0 | 0 | 0 | 0 | 0.006818±0.013635 |
| *Finegoldia* | 0 | 0 | 0 | 0.003734±0.010180 | 0 | 0 | 0.006386±0.014279 | 0 |
| *Flavisolibacter* | 0 | 0 | 0.001844±0.006115 | 0.000671±0.002225 | 0 | 0.001589±0.004766 | 0 | 0 |
| *Flavobacterium* | 0.034730±0.046643 | 0.036269±0.020488 | 0.071428±0.071958 | 0.010903±0.013754 | 0.022825±0.025520 | 0.037486±0.039341 | 0.030795±0.022871 | 0.057674±0.042597 |
| *Flavonifractor* | 0.002158±0.006475 | 0.002008±0.006661 | 0.001093±0.003625 | 0.003751±0.012441 | 0 | 0 | 0 | 0 |
| *Fusobacterium* | 0.004515±0.013545 | 0.002494±0.008272 | 0.010141±0.033634 | 0 | 0.003102±0.009306 | 0 | 0 | 0.012057±0.014159 |
| *GW-34* | 0 | 0.002522±0.008364 | 0 | 0.005463±0.012186 | 0.002158±0.006474 | 0.003044±0.009132 | 0 | 0 |
| *Gallionella* | 0 | 0 | 0 | 0 | 0.001510±0.004529 | 0.001589±0.004766 | 0 | 0 |
| *Gemella* | 0.001702±0.005105 | 0.000847±0.002810 | 0.002015±0.004503 | 0.000671±0.002225 | 0 | 0.003177±0.009532 | 0 | 0 |
| *Gemmobacter* | 0.001129±0.003386 | 0.008600±0.018076 | 0 | 0 | 0 | 0 | 0 | 0 |
| *Geobacillus* | 0.001695±0.005084 | 0.000669±0.002220 | 0 | 0 | 0 | 0 | 0.001356±0.003032 | 0 |
| *Geodermatophilus* | 0 | 0 | 0.000922±0.003058 | 0 | 0 | 0 | 0 | 0 |
| *Georgenia* | 0 | 0 | 0 | 0.001876±0.006221 | 0 | 0 | 0 | 0 |
| *Globicatella* | 0 | 0 | 0 | 0 | 0 | 0 | 0.002385±0.005332 | 0 |
| *Gossypium* | 0 | 0 | 0.020265±0.055481 | 0.031145±0.092674 | 0.033079±0.074088 | 0.017177±0.021746 | 0.001356±0.003032 | 0 |
| *Gracilibacillus* | 0 | 0 | 0 | 0 | 0 | 0.003498±0.010495 | 0 | 0 |
| *Granulicatella* | 0.003916±0.011747 | 0 | 0.002046±0.004578 | 0 | 0 | 0.003239±0.009718 | 0 | 0 |
| *Gulosibacter* | 0 | 0 | 0.002459±0.008156 | 0.001112±0.003687 | 0.001003±0.003010 | 0 | 0 | 0 |
| *HTCC* | 0 | 0 | 0.000922±0.003058 | 0 | 0 | 0 | 0.002711±0.006063 | 0 |
| *Haemophilus* | 0.002823±0.005745 | 0.005969±0.019797 | 0.005102±0.008861 | 0.001876±0.006221 | 0.025260±0.064678 | 0.004672±0.014017 | 0.002385±0.005332 | 0.009044±0.006165 |
| *Hafnia* | 0.016166±0.023054 | 0.036373±0.047713 | 0.040059±0.046208 | 0.028396±0.025681 | 0.018649±0.021072 | 0.028979±0.052329 | 0.020058±0.028270 | 0.011664±0.008688 |
| *Halolactibacillus* | 0 | 0 | 0 | 0 | 0.002158±0.006474 | 0 | 0 | 0 |
| *Halomonas* | 0 | 0.001694±0.005619 | 0.003268±0.006005 | 0.010934±0.019747 | 0.025632±0.049852 | 0 | 0.001356±0.003032 | 0.025158±0.027014 |
| *Halospirulina* | 0 | 0 | 0.005531±0.018346 | 0 | 0 | 0 | 0 | 0 |
| *Hansschlegelia* | 0.007039±0.011325 | 0.014595±0.029058 | 0 | 0 | 0 | 0 | 0 | 0 |
| *Helcococcus* | 0 | 0 | 0 | 0.001531±0.005079 | 0 | 0 | 0 | 0.005239±0.010479 |
| *Herbaspirillum* | 0.003087±0.006403 | 0.000393±0.001304 | 0 | 0 | 0 | 0 | 0 | 0 |
| *Howardella* | 0 | 0 | 0 | 0 | 0 | 0 | 0 | 0.005239±0.010479 |
| *Hydrogenophilus* | 0 | 0.000393±0.001304 | 0 | 0.001876±0.006221 | 0 | 0 | 0 | 0 |
| *Hylemonella* | 0.003722±0.008088 | 0.002494±0.008272 | 0 | 0.001531±0.005079 | 0.002129±0.004234 | 0 | 0.012838±0.013703 | 0 |
| *Hyphomicrobium* | 0.024408±0.037337 | 0.044807±0.068287 | 0.001901±0.006305 | 0.004405±0.010675 | 0 | 0 | 0.001356±0.003032 | 0 |
| *Ideonella* | 0.003624±0.007221 | 0 | 0.004372±0.014501 | 0 | 0.002251±0.006754 | 0 | 0 | 0.012455±0.017075 |
| *Ilumatobacter* | 0 | 0 | 0.000922±0.003058 | 0 | 0 | 0 | 0 | 0 |
| *Insolitispirillum* | 0.001129±0.003386 | 0 | 0 | 0 | 0.002251±0.006754 | 0.006479±0.019436 | 0 | 0 |
| *Janibacter* | 0 | 0 | 0.000757±0.002509 | 0 | 0 | 0 | 0 | 0 |
| *Janthinobacterium* | 0.034894±0.044447 | 0.039932±0.052349 | 0.062107±0.057373 | 0.039634±0.042091 | 0.035659±0.039896 | 0.031598±0.032738 | 0.021188±0.027945 | 0.065944±0.050806 |
| *Jeotgalicoccus* | 0.014629±0.030517 | 0.025693±0.043907 | 0.055414±0.075971 | 0.048550±0.065134 | 0.101368±0.146659 | 0.102726±0.102324 | 0.046898±0.055144 | 0.119133±0.094545 |
| *Kandleria* | 0 | 0 | 0 | 0 | 0 | 0 | 0 | 0.002620±0.005239 |
| *Ketogulonicigenium* | 0 | 0 | 0.002459±0.008156 | 0.003063±0.010159 | 0 | 0 | 0 | 0 |
| *Kineococcus* | 0 | 0 | 0.000757±0.002509 | 0 | 0 | 0 | 0 | 0 |
| *Klebsiella* | 0.001129±0.003386 | 0.005526±0.010503 | 0.005072±0.010397 | 0 | 0 | 0 | 0.001356±0.003032 | 0 |
| *Kluyvera* | 0.001702±0.005105 | 0.001517±0.003403 | 0.002248±0.005100 | 0.002765±0.009170 | 0.003019±0.009058 | 0.006282±0.012593 | 0 | 0.002620±0.005239 |
| *Kocuria* | 0 | 0 | 0 | 0.002802±0.009292 | 0 | 0.003239±0.009718 | 0 | 0 |
| *Kurthia* | 0.001666±0.004999 | 0 | 0 | 0.002910±0.009651 | 0.001003±0.003010 | 0 | 0 | 0 |
| *L7A_E11* | 0 | 0 | 0 | 0 | 0 | 0.006738±0.013383 | 0 | 0 |
| *Lachnoanaerobaculum* | 0.002257±0.006772 | 0 | 0 | 0 | 0 | 0 | 0 | 0 |
| *Lactobacillus* | 1.719076±2.408360 | 0.700295±0.434469 | 2.270927±1.851149 | 0.757706±1.370755 | 1.196488±1.820100 | 0.764215±1.728530 | 3.500228±7.081650 | 1.004168±0.873187 |
| *Lactococcus* | 3.355989±1.552764 | 3.808900±1.941060 | 4.205165±2.016218 | 3.436773±1.834589 | 3.496131±1.302357 | 3.213245±1.667318 | 2.998990±1.517096 | 3.916433±1.173410 |
| *Lautropia* | 0 | 0 | 0 | 0.001876±0.006221 | 0.002007±0.006020 | 0 | 0 | 0 |
| *Leclercia* | 0 | 0.002055±0.006817 | 0 | 0 | 0 | 0 | 0.006386±0.014279 | 0 |
| *Legionella* | 0 | 0 | 0 | 0.003023±0.010027 | 0 | 0 | 0 | 0 |
| *Lelliottia* | 0 | 0 | 0 | 0 | 0 | 0.008230±0.024691 | 0 | 0 |
| *Leptolyngbya* | 0 | 0 | 0 | 0 | 0 | 0 | 0.002711±0.006063 | 0 |
| *Leptotrichia* | 0 | 0 | 0 | 0 | 0.006204±0.018612 | 0 | 0 | 0 |
| *Leucobacter* | 0 | 0.002001±0.006637 | 0.001093±0.003625 | 0 | 0 | 0 | 0 | 0.006029±0.007079 |
| *Leuconostoc* | 0.093866±0.093111 | 0.093841±0.075004 | 0.109217±0.079670 | 0.089529±0.051818 | 0.122628±0.137882 | 0.056960±0.045598 | 0.094003±0.073323 | 0.071054±0.085481 |
| *Limnobacter* | 0.719482±0.980348 | 2.717630±2.571015 | 0.002270±0.007528 | 0.001607±0.005329 | 0 | 0 | 0.002711±0.006063 | 0 |
| *Limnohabitans* | 0.001129±0.003386 | 0 | 0.000757±0.002509 | 0 | 0 | 0 | 0 | 0 |
| *Lotus* | 0 | 0 | 0.002766±0.009173 | 0 | 0 | 0 | 0 | 0 |
| *Luteimonas* | 0.003652±0.007272 | 0 | 0.013258±0.019025 | 0.016597±0.024577 | 0.025110±0.029679 | 0.010593±0.016530 | 0.018261±0.020892 | 0.032377±0.051735 |
| *Luteococcus* | 0 | 0.001001±0.003318 | 0 | 0 | 0 | 0 | 0 | 0 |
| *Lutibacterium* | 0 | 0.000847±0.002810 | 0.000922±0.003058 | 0 | 0 | 0 | 0 | 0 |
| *Lysinibacillus* | 0.946027±0.183121 | 0.978156±0.342553 | 1.062348±0.249574 | 0.949047±0.226798 | 0.925773±0.122512 | 0.946518±0.228768 | 0.877723±0.285854 | 1.116524±0.218538 |
| *Lysobacter* | 0.024937±0.040462 | 0.050911±0.058629 | 0.000922±0.003058 | 0 | 0.002158±0.006474 | 0.003925±0.007968 | 0 | 0 |
| *Macellibacteroides* | 0.006807±0.020422 | 0 | 0 | 0 | 0 | 0 | 0 | 0 |
| *Macrococcus* | 0.001129±0.003386 | 0.000847±0.002810 | 0.000757±0.002509 | 0.002873±0.006410 | 0.005628±0.016884 | 0 | 0.005096±0.007002 | 0.011270±0.013078 |
| *Magnetospirillum* | 0.018226±0.026718 | 0.107765±0.110804 | 0 | 0.002765±0.009170 | 0 | 0 | 0 | 0.003409±0.006818 |
| *Mannheimia* | 0 | 0 | 0 | 0 | 0 | 0 | 0.002385±0.005332 | 0 |
| *Marinobacter* | 0.001958±0.005874 | 0 | 0 | 0 | 0 | 0 | 0.001356±0.003032 | 0.011270±0.013078 |
| *Marixanthomonas* | 0 | 0 | 0.001093±0.003625 | 0 | 0 | 0 | 0 | 0.003409±0.006818 |
| *Massilia* | 0.080349±0.097006 | 0.085428±0.074152 | 0.009945±0.017637 | 0.017795±0.030079 | 0.019485±0.017318 | 0 | 0.014520±0.020121 | 0.008255±0.010200 |
| *Megamonas* | 0 | 0 | 0 | 0 | 0 | 0 | 0.019157±0.042837 | 0 |
| *Megasphaera* | 0 | 0 | 0 | 0 | 0 | 0 | 0 | 0.003015±0.006031 |
| *Mesorhizobium* | 0.001129±0.003386 | 0 | 0 | 0 | 0 | 0 | 0.012771±0.028558 | 0 |
| *Methylobacterium* | 0.394877±0.527143 | 1.330379±1.351561 | 0.007577±0.008230 | 0.014604±0.021255 | 0.013453±0.027139 | 0.011089±0.017064 | 0.011809±0.016259 | 0.046145±0.078938 |
| *Methylophilus* | 0 | 0.006025±0.019984 | 0 | 0 | 0 | 0 | 0 | 0 |
| *Methylopila* | 0 | 0 | 0 | 0.002765±0.009170 | 0 | 0 | 0 | 0 |
| *Methyloversatilis* | 0.001885±0.005655 | 0.001339±0.004441 | 0.005531±0.018346 | 0 | 0.001126±0.003377 | 0 | 0 | 0 |
| *Microbacterium* | 0.001885±0.005655 | 0 | 0 | 0.000671±0.002225 | 0 | 0 | 0.004769±0.010664 | 0.003409±0.006818 |
| *Micrococcus* | 0 | 0 | 0 | 0.000671±0.002225 | 0.003546±0.010639 | 0 | 0.003740±0.005435 | 0.002620±0.005239 |
| *Microvirgula* | 0.001666±0.004999 | 0 | 0 | 0 | 0.003546±0.010639 | 0 | 0 | 0.015077±0.030153 |
| *Mitsuaria* | 0 | 0 | 0.003979±0.013195 | 0.004886±0.011759 | 0 | 0 | 0 | 0 |
| *Mobilicoccus* | 0 | 0 | 0 | 0.002910±0.009651 | 0 | 0 | 0 | 0 |
| *Mogibacterium* | 0.004317±0.012950 | 0.001694±0.005619 | 0.009289±0.016886 | 0.010205±0.025361 | 0.002968±0.008903 | 0 | 0.007741±0.013836 | 0 |
| *Moheibacter* | 0.001958±0.005874 | 0 | 0 | 0 | 0 | 0 | 0 | 0 |
| *Moranella* | 0 | 0.011675±0.036348 | 0 | 0 | 0 | 0 | 0 | 0 |
| *Mucispirillum* | 0 | 0 | 0.001326±0.004398 | 0 | 0 | 0 | 0 | 0 |
| *Mycobacterium* | 0 | 0 | 0 | 0 | 0.003102±0.009306 | 0 | 0 | 0 |
| *Mycoplasma* | 0 | 0 | 0 | 0.001876±0.006221 | 0 | 0 | 0 | 0 |
| *Myroides* | 0.041895±0.053285 | 0.040768±0.036140 | 0.068095±0.058658 | 0.041118±0.032800 | 0.068620±0.068796 | 0.038850±0.047802 | 0.073949±0.063893 | 0.040232±0.033099 |
| *Naxibacter* | 0.035397±0.032213 | 0.048260±0.034911 | 0.004080±0.009106 | 0.005076±0.011584 | 0.003546±0.010639 | 0 | 0 | 0 |
| *Nocardioides* | 0 | 0 | 0 | 0 | 0.001126±0.003377 | 0 | 0 | 0 |
| *Noviherbaspirillum* | 0.014005±0.035046 | 0.020339±0.023817 | 0 | 0.005283±0.012959 | 0.003639±0.005586 | 0 | 0 | 0 |
| *Novosphingobium* | 0 | 0.001193±0.003957 | 0 | 0 | 0.002968±0.008903 | 0 | 0 | 0.003015±0.006031 |
| *Nubsella* | 0 | 0 | 0 | 0.002684±0.008901 | 0 | 0 | 0 | 0 |
| *Obesumbacterium* | 0 | 0 | 0 | 0 | 0 | 0.002743±0.008230 | 0 | 0 |
| *Oceanobacillus* | 2.394675±1.208728 | 2.622483±1.156581 | 2.862643±0.771261 | 2.218614±0.434315 | 2.786484±1.376920 | 2.484634±0.936363 | 3.555012±1.551620 | 3.500197±0.714942 |
| *Ochrobactrum* | 0.001702±0.005105 | 0.000669±0.002220 | 0.001093±0.003625 | 0.002278±0.005566 | 0.002007±0.006020 | 0 | 0.002385±0.005332 | 0.006818±0.013635 |
| *Odoribacter* | 0 | 0.001892±0.006275 | 0.000631±0.002094 | 0.000671±0.002225 | 0 | 0 | 0 | 0 |
| *Oligella* | 0 | 0 | 0 | 0 | 0 | 0 | 0 | 0.002620±0.005239 |
| *Oribacterium* | 0.001129±0.003386 | 0 | 0 | 0 | 0 | 0 | 0 | 0 |
| *Ornithinibacillus* | 0 | 0.000847±0.002810 | 0 | 0 | 0 | 0 | 0 | 0 |
| *Ornithinimicrobium* | 0 | 0 | 0 | 0 | 0 | 0 | 0.001356±0.003032 | 0.006818±0.013635 |
| *Oscillibacter* | 0.010498±0.008265 | 0.024328±0.038635 | 0.031197±0.033381 | 0.032408±0.038676 | 0.082043±0.201949 | 0.029216±0.031688 | 0.023291±0.028115 | 0.021890±0.023400 |
| *Oscillospira* | 0.003843±0.007628 | 0.002364±0.005832 | 0.011929±0.026267 | 0.006516±0.014699 | 0.066030±0.175425 | 0.006479±0.019436 | 0 | 0 |
| *Paenalcaligenes* | 0 | 0 | 0.000631±0.002094 | 0 | 0.005126±0.010333 | 0 | 0 | 0 |
| *Paenibacillus* | 0.030626±0.022020 | 0.074695±0.029947 | 0.074008±0.059033 | 0.070528±0.055290 | 0.053014±0.081718 | 0.047317±0.050337 | 0.085484±0.086974 | 0.158462±0.112337 |
| *Paludibacter* | 0.001958±0.005874 | 0.005036±0.011204 | 0.002270±0.007528 | 0.003751±0.012441 | 0 | 0.009412±0.016911 | 0.002711±0.006063 | 0.003015±0.006031 |
| *Pantoea* | 0.006553±0.010036 | 0.010853±0.033022 | 0.009576±0.012277 | 0.014138±0.029213 | 0.032529±0.092601 | 0.001589±0.004766 | 0.543222±1.077585 | 0.029103±0.036500 |
| *Parabacteroides* | 0 | 0 | 0 | 0 | 0.001126±0.003377 | 0.003239±0.009718 | 0 | 0.005239±0.010479 |
| *Paracoccus* | 0.024905±0.044403 | 0.010050±0.017443 | 0.039363±0.034329 | 0.048059±0.070510 | 0.014298±0.015992 | 0.029077±0.028096 | 0.032919±0.025541 | 0.046158±0.022202 |
| *Paraprevotella* | 0 | 0 | 0.002459±0.008156 | 0.004333±0.010134 | 0 | 0.003498±0.010495 | 0 | 0.002620±0.005239 |
| *Parapusillimonas* | 0 | 0 | 0 | 0.002553±0.008467 | 0 | 0 | 0 | 0 |
| *Parasutterella* | 0.004116±0.008180 | 0.003389±0.011239 | 0.001093±0.003625 | 0.004846±0.010397 | 0.012408±0.037223 | 0 | 0 | 0 |
| *Pectinatus* | 0 | 0.001694±0.005619 | 0 | 0 | 0 | 0 | 0 | 0 |
| *Pediococcus* | 0.003916±0.011747 | 0.009615±0.027021 | 0.002279±0.005183 | 0.003213±0.010658 | 0.007604±0.015415 | 0.006479±0.019436 | 0.009490±0.021221 | 0.019422±0.015590 |
| *Pedobacter* | 0.009260±0.019208 | 0.012290±0.019640 | 0.011116±0.019692 | 0.005777±0.016858 | 0.008199±0.011576 | 0 | 0.001356±0.003032 | 0.015470±0.022776 |
| *Pelobacter* | 0.001702±0.005105 | 0 | 0 | 0 | 0 | 0.015205±0.031638 | 0 | 0 |
| *Pelomonas* | 0.010706±0.016115 | 0.021286±0.023590 | 0.008839±0.017450 | 0.004749±0.008242 | 0.001126±0.003377 | 0.001589±0.004766 | 0.002711±0.006063 | 0.002620±0.005239 |
| *Peptoniphilus* | 0 | 0 | 0 | 0.001531±0.005079 | 0 | 0.002743±0.008230 | 0.002385±0.005332 | 0.006424±0.007446 |
| *Peptostreptococcus* | 0.001666±0.004999 | 0 | 0 | 0 | 0 | 0 | 0 | 0 |
| *Perlucidibaca* | 0.001702±0.005105 | 0 | 0 | 0 | 0 | 0 | 0 | 0 |
| *Petrimonas* | 0 | 0.001193±0.003957 | 0 | 0 | 0 | 0 | 0.001356±0.003032 | 0 |
| *Phascolarctobacterium* | 0.007157±0.015574 | 0.019555±0.022148 | 0.039188±0.036308 | 0.034721±0.047671 | 0.047484±0.092539 | 0.016023±0.025646 | 0.020930±0.026519 | 0.012332±0.008988 |
| *Phenylobacterium* | 0 | 0 | 0 | 0 | 0 | 0 | 0 | 0.003409±0.006818 |
| *Phormidium* | 0 | 0 | 0 | 0 | 0 | 0.002336±0.007009 | 0 | 0 |
| *Phycisphaera* | 0 | 0.001001±0.003318 | 0 | 0 | 0 | 0 | 0 | 0 |
| *Pinus* | 0 | 0.001001±0.003318 | 0.001263±0.004188 | 0 | 0.001126±0.003377 | 0 | 0 | 0 |
| *Pirellula* | 0 | 0.002001±0.006637 | 0 | 0 | 0 | 0 | 0 | 0 |
| *Planctomyces* | 0 | 0 | 0.000922±0.003058 | 0 | 0 | 0 | 0 | 0 |
| *Planobacterium* | 0 | 0 | 0 | 0 | 0 | 0 | 0.002385±0.005332 | 0 |
| *Planococcus* | 0.035536±0.090227 | 0.018009±0.026632 | 0.059663±0.092517 | 0.039109±0.040450 | 0.100042±0.143519 | 0.048520±0.061956 | 0.007831±0.012012 | 0.152827±0.214377 |
| *Planomicrobium* | 0.051569±0.075379 | 0.050455±0.129332 | 0.223204±0.339450 | 0.157770±0.167593 | 0.303375±0.421662 | 0.280906±0.425139 | 0.097548±0.087628 | 0.335513±0.387589 |
| *Pleomorphomonas* | 0 | 0.003085±0.007076 | 0 | 0 | 0 | 0 | 0 | 0 |
| *Polaromonas* | 0 | 0.002678±0.008882 | 0 | 0.001342±0.004450 | 0.002007±0.006020 | 0 | 0 | 0.002620±0.005239 |
| *Pontibacter* | 0.001129±0.003386 | 0.001001±0.003318 | 0 | 0 | 0 | 0 | 0 | 0 |
| *Porphyrobacter* | 0.004288±0.008746 | 0 | 0.002450±0.005473 | 0.002553±0.008467 | 0 | 0.004828±0.010275 | 0.001356±0.003032 | 0 |
| *Porphyromonas* | 0 | 0 | 0.000922±0.003058 | 0 | 0 | 0 | 0 | 0 |
| *Prevotella* | 0.003087±0.006403 | 0.009829±0.016626 | 0.048283±0.102337 | 0.010250±0.016314 | 0.007207±0.018478 | 0.006489±0.012877 | 0.011809±0.016259 | 0.018086±0.021238 |
| *Propionibacterium* | 0.026109±0.037999 | 0.008956±0.016832 | 0.029073±0.027870 | 0.034902±0.057761 | 0.061082±0.127601 | 0.023087±0.025185 | 0.033350±0.053914 | 0.025553±0.023299 |
| *Proteiniclasticum* | 0.008937±0.020555 | 0 | 0.007945±0.018277 | 0.006304±0.014332 | 0.004672±0.010753 | 0.003044±0.009132 | 0 | 0.004077±0.008154 |
| *Proteus* | 0 | 0 | 0 | 0 | 0 | 0.005576±0.011249 | 0 | 0 |
| *Providencia* | 0 | 0 | 0 | 0 | 0.002158±0.006474 | 0 | 0 | 0 |
| *Pseudochrobactrum* | 0 | 0 | 0 | 0.002013±0.006676 | 0.001126±0.003377 | 0.001589±0.004766 | 0 | 0.003409±0.006818 |
| *Pseudoflavonifractor* | 0 | 0.002893±0.006799 | 0.002015±0.004503 | 0 | 0.014981±0.027833 | 0.003239±0.009718 | 0 | 0.003015±0.006031 |
| *Pseudomonas* | 4.433029±2.539996 | 6.275579±2.834985 | 3.806089±1.662107 | 3.559977±1.762035 | 3.856588±1.658489 | 3.089011±1.633588 | 3.122765±1.936296 | 4.309378±1.859430 |
| *Pseudoxanthomonas* | 0 | 0.000393±0.001304 | 0.003046±0.006897 | 0.002546±0.006394 | 0 | 0.003498±0.010495 | 0 | 0 |
| *Psychrobacillus* | 0.001129±0.003386 | 0.001586±0.004041 | 0 | 0 | 0 | 0 | 0 | 0 |
| *Psychrobacter* | 0.794024±0.601109 | 0.690759±0.430846 | 0.895574±0.513929 | 0.870298±0.624359 | 0.905316±0.437359 | 0.787998±0.502050 | 0.594144±0.332252 | 0.991136±0.426038 |
| *Rahnella* | 0.014609±0.024469 | 0.017853±0.021414 | 0.024788±0.027802 | 0.014976±0.021625 | 0.015018±0.020444 | 0.024399±0.035432 | 0.020058±0.028270 | 0.052039±0.039774 |
| *Ralstonia* | 0.001702±0.005105 | 0.013443±0.027134 | 0.128639±0.380227 | 0.011506±0.017663 | 0 | 0.003044±0.009132 | 0.002711±0.006063 | 0.006029±0.007079 |
| *Ramlibacter* | 0.007246±0.011555 | 0.002522±0.008364 | 0 | 0 | 0 | 0 | 0.001356±0.003032 | 0 |
| *Raoultella* | 0 | 0 | 0 | 0.000671±0.002225 | 0.003546±0.010639 | 0.005487±0.016461 | 0.002711±0.006063 | 0 |
| *Rheinheimera* | 0.002594±0.007781 | 0.000847±0.002810 | 0.011050±0.015735 | 0.006918±0.012593 | 0 | 0.002336±0.007009 | 0 | 0.006029±0.007079 |
| *Rhizobacter* | 0.005610±0.012203 | 0.005775±0.016489 | 0.001844±0.006115 | 0 | 0.004503±0.013508 | 0 | 0 | 0 |
| *Rhizobium* | 0.003087±0.006403 | 0.017341±0.028516 | 0.006561±0.012917 | 0.006538±0.009847 | 0.006479±0.012871 | 0.007009±0.021026 | 0.004067±0.009095 | 0 |
| *Rhodobacter* | 0.015334±0.034613 | 0.009497±0.018267 | 0.018077±0.022482 | 0.008857±0.019985 | 0.016699±0.041918 | 0.006088±0.018265 | 0.018194±0.028097 | 0.006818±0.013635 |
| *Rhodococcus* | 0 | 0 | 0.001679±0.003757 | 0.001531±0.005079 | 0 | 0 | 0 | 0 |
| *Rhodocytophaga* | 0 | 0 | 0.003067±0.005698 | 0 | 0 | 0.003044±0.009132 | 0 | 0 |
| *Rhodoferax* | 0 | 0 | 0.000631±0.002094 | 0 | 0.003019±0.009058 | 0.004766±0.014298 | 0 | 0 |
| *Rhodopirellula* | 0 | 0 | 0 | 0 | 0 | 0 | 0 | 0.003409±0.006818 |
| *Roseateles* | 0 | 0.001892±0.006275 | 0 | 0 | 0 | 0 | 0 | 0 |
| *Roseburia* | 0.002795±0.005677 | 0.005156±0.011510 | 0.012135±0.011552 | 0.016770±0.037057 | 0.007983±0.019383 | 0.012762±0.029442 | 0.027927±0.056021 | 0.013393±0.016181 |
| *Roseivivax* | 0 | 0 | 0 | 0 | 0 | 0 | 0.012771±0.028558 | 0 |
| *Roseococcus* | 0 | 0 | 0 | 0.001607±0.005329 | 0 | 0 | 0 | 0 |
| *Roseomonas* | 0 | 0 | 0.001326±0.004398 | 0.004594±0.015238 | 0 | 0 | 0.001356±0.003032 | 0 |
| *Rothia* | 0 | 0.001001±0.003318 | 0 | 0.000671±0.002225 | 0 | 0 | 0 | 0 |
| *Rubellimicrobium* | 0 | 0.005523±0.012345 | 0.000922±0.003058 | 0.002873±0.006410 | 0 | 0.006738±0.013383 | 0.005423±0.012126 | 0 |
| *Rubrobacter* | 0 | 0 | 0.000922±0.003058 | 0 | 0 | 0 | 0 | 0 |
| *Ruminobacter* | 0.004525±0.006947 | 0.008274±0.017248 | 0.007251±0.013158 | 0.002553±0.008467 | 0.008096±0.021115 | 0.002743±0.008230 | 0 | 0.007859±0.015718 |
| *Ruminococcus* | 0.001666±0.004999 | 0.010026±0.022662 | 0.035955±0.050519 | 0.024151±0.037054 | 0.042928±0.110137 | 0.010666±0.016620 | 0.001356±0.003032 | 0.034868±0.033265 |
| *Rummeliibacillus* | 0 | 0 | 0 | 0.001112±0.003687 | 0.001003±0.003010 | 0 | 0 | 0 |
| *Salinicoccus* | 0 | 0 | 0 | 0 | 0 | 0 | 0 | 0.004077±0.008154 |
| *Salmonella* | 0 | 0.004002±0.013274 | 0.001124±0.003727 | 0.013758±0.036010 | 0 | 0.003239±0.009718 | 0 | 0.003409±0.006818 |
| *Sandaracinobacter* | 0 | 0 | 0.002186±0.007250 | 0 | 0 | 0 | 0 | 0 |
| *Schlegelella* | 0 | 0 | 0.000922±0.003058 | 0 | 0 | 0 | 0 | 0 |
| *Sediminibacterium* | 0 | 0.003248±0.007532 | 0 | 0 | 0 | 0 | 0 | 0 |
| *Selenomonas* | 0 | 0 | 0 | 0 | 0 | 0.001589±0.004766 | 0 | 0 |
| *Serpens* | 0 | 0.002285±0.006280 | 0 | 0 | 0 | 0 | 0 | 0.003015±0.006031 |
| *Serratia* | 0.009321±0.016018 | 0.015229±0.030055 | 0.018339±0.026069 | 0.011328±0.013487 | 0.014589±0.012430 | 0.039195±0.033286 | 0.010192±0.014004 | 0.010874±0.014840 |
| *Sharpea* | 0 | 0 | 0.002652±0.008797 | 0 | 0 | 0 | 0 | 0 |
| *Shewanella* | 0.002594±0.007781 | 0.002770±0.009187 | 0.000631±0.002094 | 0 | 0 | 0.002336±0.007009 | 0.001356±0.003032 | 0 |
| *Shigella* | 0 | 0 | 0.002019±0.004662 | 0.001876±0.006221 | 0.011216±0.027136 | 0 | 0 | 0 |
| *Shinella* | 0.009161±0.017748 | 0.028373±0.030714 | 0 | 0 | 0 | 0.002743±0.008230 | 0 | 0 |
| *Simplicispira* | 0 | 0 | 0.001093±0.003625 | 0 | 0 | 0.003498±0.010495 | 0 | 0.007486±0.008712 |
| *Sneathia* | 0 | 0 | 0 | 0 | 0.003546±0.010639 | 0 | 0 | 0.003015±0.006031 |
| *Solibacillus* | 0.236037±0.106298 | 0.330515±0.165167 | 0.348011±0.088842 | 0.251275±0.096085 | 0.291369±0.156814 | 0.248062±0.085114 | 0.303996±0.183781 | 0.316799±0.135235 |
| *Solobacterium* | 0 | 0 | 0.002459±0.008156 | 0 | 0 | 0 | 0 | 0 |
| *Sorangium* | 0 | 0 | 0.011985±0.039749 | 0 | 0 | 0 | 0 | 0 |
| *Sphingobacterium* | 0 | 0.003689±0.007233 | 0.004309±0.008610 | 0.011196±0.025675 | 0.006770±0.010775 | 0 | 0 | 0.008648±0.010424 |
| *Sphingobium* | 0.004288±0.008746 | 0.003556±0.009296 | 0 | 0 | 0.001003±0.003010 | 0 | 0 | 0 |
| *Sphingomonas* | 0.020166±0.028149 | 0.033720±0.044319 | 0.035132±0.032596 | 0.021861±0.031739 | 0.016859±0.016413 | 0.031656±0.036288 | 0.016971±0.026696 | 0.016134±0.011656 |
| *Sphingopyxis* | 0.004781±0.007421 | 0.007141±0.011660 | 0.004918±0.016311 | 0.002553±0.008467 | 0.003392±0.010175 | 0 | 0 | 0 |
| *Sphingorhabdus* | 0 | 0.001339±0.004441 | 0 | 0 | 0 | 0 | 0 | 0 |
| *Spinacia* | 0 | 0 | 0.000631±0.002094 | 0.001112±0.003687 | 0 | 0.001589±0.004766 | 0 | 0 |
| *Spiroplasma* | 0 | 0.001892±0.006275 | 0 | 0 | 0 | 0 | 0 | 0 |
| *Sporobacter* | 0 | 0 | 0.002459±0.008156 | 0.000671±0.002225 | 0 | 0 | 0 | 0 |
| *Sporosarcina* | 0.016386±0.015593 | 0.018748±0.033016 | 0.019149±0.024904 | 0.020461±0.024664 | 0.012369±0.020504 | 0.015593±0.029407 | 0.020908±0.023795 | 0.041041±0.022302 |
| *Staphylococcus* | 0.051489±0.062627 | 0.862907±2.542020 | 0.315108±0.883944 | 1.074250±2.227805 | 0.980499±2.432808 | 2.547565±2.818541 | 1.276792±1.616044 | 1.067776±1.775183 |
| *Stenotrophomonas* | 0.097381±0.077997 | 0.107391±0.127629 | 0.217371±0.254762 | 0.195107±0.276393 | 0.114639±0.103708 | 0.063673±0.062818 | 0.371324±0.432924 | 0.723708±0.523018 |
| *Streptococcus* | 0.159471±0.149707 | 0.302679±0.424537 | 0.168029±0.077126 | 2.236082±7.073181 | 0.851315±2.131772 | 0.386244±0.840651 | 0.186608±0.259947 | 0.385071±0.634882 |
| *Succinivibrio* | 0 | 0.007210±0.019609 | 0.006306±0.016145 | 0.002546±0.006394 | 0.002158±0.006474 | 0.002336±0.007009 | 0.008158±0.012172 | 0.006697±0.008091 |
| *Sulfurospirillum* | 0 | 0 | 0 | 0.001876±0.006221 | 0.002007±0.006020 | 0.003239±0.009718 | 0.001356±0.003032 | 0.005635±0.006539 |
| *Sunxiuqinia* | 0.001885±0.005655 | 0 | 0 | 0 | 0 | 0 | 0 | 0 |
| *Sutterella* | 0 | 0.000847±0.002810 | 0.000922±0.003058 | 0 | 0.006204±0.018612 | 0 | 0 | 0.003015±0.006031 |
| *Tardiphaga* | 0 | 0.000393±0.001304 | 0 | 0 | 0 | 0 | 0 | 0 |
| *Terribacillus* | 0 | 0 | 0 | 0 | 0 | 0 | 0 | 0.005239±0.010479 |
| *Terrimonas* | 0 | 0.000669±0.002220 | 0 | 0 | 0 | 0 | 0.006386±0.014279 | 0 |
| *Tessaracoccus* | 0 | 0 | 0 | 0.001876±0.006221 | 0.002158±0.006474 | 0 | 0 | 0 |
| *Tetragenococcus* | 0 | 0.000847±0.002810 | 0 | 0 | 0 | 0 | 0 | 0 |
| *Thermoactinomyces* | 0 | 0 | 0 | 0.002910±0.009651 | 0 | 0 | 0 | 0 |
| *Thermomonas* | 0 | 0.001339±0.004441 | 0 | 0 | 0 | 0 | 0 | 0 |
| *Thermus* | 0 | 0.000669±0.002220 | 0 | 0 | 0 | 0 | 0 | 0 |
| *Tissierella* | 0 | 0 | 0 | 0 | 0 | 0.003044±0.009132 | 0 | 0 |
| *Tissierella_Soehngenia* | 0 | 0 | 0 | 0.001876±0.006221 | 0 | 0 | 0 | 0 |
| *Tolumonas* | 0 | 0 | 0 | 0.003023±0.010027 | 0 | 0 | 0 | 0 |
| *Trabulsiella* | 0 | 0 | 0 | 0.001112±0.003687 | 0 | 0 | 0 | 0 |
| *Treponema* | 0 | 0.002739±0.006614 | 0.008408±0.025250 | 0.011931±0.031407 | 0.003161±0.006790 | 0 | 0.020930±0.026519 | 0.004077±0.008154 |
| *Trichococcus* | 0.002795±0.005677 | 0.015195±0.028034 | 0.041872±0.095514 | 0.026179±0.044592 | 0.065885±0.092271 | 0.031962±0.030263 | 0.006602±0.006612 | 0.014182±0.013601 |
| *Truepera* | 0 | 0 | 0 | 0.000671±0.002225 | 0 | 0 | 0 | 0 |
| *Tumebacillus* | 0 | 0.002522±0.008364 | 0 | 0 | 0 | 0 | 0 | 0 |
| *Turicibacter* | 0.017424±0.031776 | 0.015016±0.027578 | 0.083272±0.181026 | 0.044207±0.045800 | 0.036531±0.047592 | 0.020994±0.021904 | 0.041010±0.044974 | 0.050782±0.037098 |
| *Uruburuella* | 0.002158±0.006475 | 0.001001±0.003318 | 0 | 0.002553±0.008467 | 0.002635±0.005300 | 0.013061±0.021300 | 0 | 0 |
| *Vagococcus* | 0.002594±0.007781 | 0 | 0.004663±0.007138 | 0.000671±0.002225 | 0.003392±0.010175 | 0 | 0.002385±0.005332 | 0 |
| *Vampirovibrio* | 0.001695±0.005084 | 0.013253±0.024176 | 0.005088±0.009729 | 0.002553±0.008467 | 0.015510±0.046529 | 0.005576±0.011249 | 0.022897±0.041053 | 0.007859±0.015718 |
| *Variovorax* | 0.006417±0.010947 | 0.025213±0.033856 | 0.005178±0.011524 | 0.004422±0.012418 | 0.003761±0.007647 | 0.005576±0.011249 | 0.001356±0.003032 | 0 |
| *Veillonella* | 0 | 0.001910±0.003459 | 0.001513±0.005019 | 0 | 0 | 0 | 0 | 0.002620±0.005239 |
| *Vibrio* | 0 | 0.000393±0.001304 | 0.000631±0.002094 | 0 | 0.006204±0.018612 | 0.003539±0.010616 | 0.006802±0.011792 | 0 |
| *Victivallis* | 0 | 0.002561±0.006443 | 0.001326±0.004398 | 0 | 0 | 0 | 0.004769±0.010664 | 0 |
| *Virgibacillus* | 0 | 0 | 0.001783±0.005914 | 0 | 0 | 0 | 0 | 0 |
| *WAL_1855D* | 0 | 0.000847±0.002810 | 0.001326±0.004398 | 0 | 0 | 0.003239±0.009718 | 0 | 0 |
| *Wautersiella* | 0.002594±0.007781 | 0 | 0.000631±0.002094 | 0.003023±0.010027 | 0 | 0 | 0 | 0 |
| *Weissella* | 0 | 0.001001±0.003318 | 0.000631±0.002094 | 0 | 0 | 0 | 0 | 0.003015±0.006031 |
| *Xanthobacter* | 0 | 0.004881±0.009260 | 0.001093±0.003625 | 0 | 0 | 0.003239±0.009718 | 0.006779±0.015158 | 0.009712±0.006927 |
| *Xanthomonas* | 0 | 0.000393±0.001304 | 0 | 0 | 0 | 0 | 0 | 0 |
| *Xenophilus* | 0 | 0.002770±0.009187 | 0 | 0 | 0 | 0 | 0 | 0 |
| *Xylophilus* | 0 | 0 | 0 | 0.001531±0.005079 | 0 | 0 | 0 | 0 |
| *YRC22* | 0 | 0 | 0.006042±0.016364 | 0.005540±0.010130 | 0.002007±0.006020 | 0.005487±0.016461 | 0 | 0.007092±0.008371 |
| *Yaniella* | 0 | 0.003002±0.009955 | 0.002459±0.008156 | 0 | 0.001003±0.003010 | 0 | 0 | 0 |
| *Yeosuana* | 0 | 0 | 0 | 0 | 0 | 0 | 0.001356±0.003032 | 0 |
| *Yersinia* | 0.005618±0.012210 | 0.000787±0.002609 | 0.000631±0.002094 | 0 | 0.010733±0.021628 | 0.002336±0.007009 | 0.001356±0.003032 | 0 |
| *Zhihengliuella* | 0 | 0 | 0.000631±0.002094 | 0 | 0 | 0 | 0 | 0 |
| *gut* | 0 | 0 | 0.001093±0.003625 | 0 | 0 | 0 | 0 | 0 |
| *rc4-4* | 0 | 0.007783±0.017122 | 0.005096±0.009359 | 0.001876±0.006221 | 0 | 0 | 0 | 0 |
| *unclassified* | 1.387132±0.638605 | 1.683214±1.089320 | 2.738079±1.314731 | 2.533275±1.171120 | 2.665688±1.565140 | 1.739747±0.465792 | 1.897730±0.836343 | 2.326966±1.369954 |
| *Acetobacter_ghanensis* | 0 | 0 | 0.001124±0.003727 | 0 | 0 | 0 | 0 | 0 |
| *Acetobacter_malorum* | 0 | 0 | 0 | 0 | 0 | 0 | 0.006386±0.014279 | 0 |
| *Acetobacter_peroxydans* | 0 | 0 | 0 | 0 | 0 | 0 | 0.001356±0.003032 | 0 |
| *Achromobacter_spanius* | 0 | 0 | 0.000631±0.002094 | 0 | 0.003546±0.010639 | 0 | 0 | 0 |
| *Acidovorax_defluvii* | 0 | 0.000787±0.002609 | 0 | 0 | 0 | 0 | 0 | 0 |
| *Acidovorax_facilis* | 0 | 0.000393±0.001304 | 0.001326±0.004398 | 0 | 0 | 0 | 0 | 0 |
| *Acidovorax_radicis* | 0.048764±0.086352 | 0.176406±0.169597 | 0.009416±0.014855 | 0.001863±0.006180 | 0.001003±0.003010 | 0.004828±0.010275 | 0.011482±0.013098 | 0.006818±0.013635 |
| *Acidovorax_soli* | 0.002257±0.006772 | 0.003163±0.009149 | 0 | 0 | 0 | 0 | 0 | 0 |
| *Acidovorax_temperans* | 0.003014±0.006218 | 0.001063±0.002460 | 0.008393±0.018783 | 0.016009±0.036920 | 0.001510±0.004529 | 0.003539±0.010616 | 0.004067±0.009095 | 0.003409±0.006818 |
| *Acidovorax_valerianellae* | 0 | 0.005448±0.010535 | 0 | 0.002553±0.008467 | 0 | 0 | 0 | 0 |
| *Acidovorax_wohlfahrtii* | 0.001129±0.003386 | 0 | 0 | 0.000671±0.002225 | 0.006039±0.018116 | 0 | 0 | 0 |
| *Acinetobacter_baumannii* | 0.016978±0.022800 | 0.035388±0.023402 | 0.046338±0.075658 | 0.026657±0.062913 | 0.020812±0.020427 | 0.020735±0.028035 | 0 | 0.017301±0.022873 |
| *Acinetobacter_calcoaceticus* | 0.002823±0.005745 | 0.004988±0.016544 | 0.005713±0.007386 | 0.011254±0.014931 | 0.007631±0.015479 | 0.008691±0.019472 | 0.003740±0.005435 | 0.002620±0.005239 |
| *Acinetobacter_guillouiae* | 0.002594±0.007781 | 0.000393±0.001304 | 0.007111±0.009204 | 0.001531±0.005079 | 0.003019±0.009058 | 0.001589±0.004766 | 0.063857±0.142789 | 0.009833±0.012941 |
| *Acinetobacter_gyllenbergii* | 0.003825±0.007670 | 0.003033±0.006805 | 0.005208±0.013684 | 0 | 0 | 0 | 0.003740±0.005435 | 0 |
| *Acinetobacter_haemolyticus* | 0.002594±0.007781 | 0 | 0 | 0.006247±0.012328 | 0.012149±0.031673 | 0.012564±0.025187 | 0.008308±0.012260 | 0 |
| *Acinetobacter_indicus* | 0.040832±0.036088 | 0.017043±0.018094 | 0.008382±0.012795 | 0.017072±0.025491 | 0.032486±0.050080 | 0.009846±0.015887 | 0.002385±0.005332 | 0.002620±0.005239 |
| *Acinetobacter_johnsonii* | 0.024660±0.040471 | 0.072893±0.092232 | 0.174514±0.234204 | 0.069873±0.115826 | 0.757186±1.552763 | 0.688745±0.699451 | 0.051677±0.057542 | 0.074179±0.052954 |
| *Acinetobacter_junii* | 0 | 0 | 0.000631±0.002094 | 0.005099±0.009913 | 0 | 0.002743±0.008230 | 0 | 0.005635±0.006539 |
| *Acinetobacter_lwoffii* | 0.189194±0.233278 | 0.187836±0.141155 | 0.091558±0.070279 | 0.096447±0.112132 | 0.398835±0.780152 | 0.245421±0.353030 | 0.088264±0.101522 | 0.121407±0.094250 |
| *Acinetobacter_parvus* | 0 | 0.000847±0.002810 | 0 | 0.001863±0.006180 | 0.006039±0.018116 | 0 | 0 | 0 |
| *Acinetobacter_radioresistens* | 0 | 0 | 0 | 0.003335±0.011061 | 0 | 0 | 0 | 0 |
| *Acinetobacter_rhizosphaerae* | 0 | 0.002186±0.005012 | 0.003543±0.008143 | 0 | 0.009306±0.027917 | 0 | 0 | 0 |
| *Acinetobacter_schindleri* | 0.385958±0.430476 | 0.426597±0.479972 | 0.098490±0.090343 | 0.144125±0.198455 | 0.022210±0.026516 | 0.042840±0.041378 | 0.009097±0.014048 | 0.026736±0.019603 |
| *Acinetobacter_ursingii* | 0 | 0.000393±0.001304 | 0 | 0.001863±0.006180 | 0 | 0 | 0 | 0 |
| *Actinomyces_timonensis* | 0 | 0 | 0.001124±0.003727 | 0 | 0 | 0 | 0 | 0 |
| *Adhaeribacter_terreus* | 0 | 0.001001±0.003318 | 0 | 0 | 0.001126±0.003377 | 0 | 0 | 0 |
| *Aerococcus_suis* | 0 | 0 | 0 | 0.004594±0.015238 | 0 | 0 | 0 | 0 |
| *Aerococcus_urinaeequi* | 0.010325±0.019951 | 0 | 0.005620±0.010334 | 0.006268±0.011580 | 0.060474±0.072701 | 0.013291±0.022397 | 0.038654±0.045840 | 0.080360±0.097087 |
| *Aeromonas_bestiarum* | 0 | 0 | 0.002459±0.008156 | 0 | 0 | 0 | 0 | 0 |
| *Aeromonas_caviae* | 0 | 0 | 0.001326±0.004398 | 0.000671±0.002225 | 0 | 0 | 0 | 0 |
| *Aeromonas_hydrophila* | 0.003722±0.008088 | 0.004089±0.008165 | 0.007531±0.014723 | 0.007570±0.020162 | 0.031918±0.095755 | 0.001589±0.004766 | 0.004769±0.010664 | 0.003409±0.006818 |
| *Aeromonas_molluscorum* | 0.001666±0.004999 | 0 | 0 | 0 | 0 | 0 | 0 | 0 |
| *Aeromonas_punctata* | 0 | 0.001001±0.003318 | 0.003785±0.008870 | 0.001783±0.004111 | 0 | 0 | 0 | 0 |
| *Aeromonas_salmonicida* | 0 | 0.000669±0.002220 | 0.005413±0.009519 | 0.005457±0.010850 | 0.010639±0.031918 | 0.016461±0.049383 | 0.001356±0.003032 | 0.002620±0.005239 |
| *Aeromonas_sharmana* | 0 | 0.000847±0.002810 | 0 | 0 | 0 | 0 | 0.004067±0.009095 | 0 |
| *Aeromonas_sobria* | 0 | 0 | 0.004084±0.013545 | 0 | 0.002158±0.006474 | 0 | 0.001356±0.003032 | 0 |
| *Agromyces_mediolanus* | 0 | 0 | 0.000922±0.003058 | 0 | 0 | 0 | 0 | 0 |
| *Akkermansia_muciniphila* | 0 | 0 | 0.003745±0.009173 | 0.002765±0.009170 | 0.015510±0.046529 | 0 | 0.001356±0.003032 | 0.002620±0.005239 |
| *Albidiferax_ferrireducens* | 0 | 0.002494±0.008272 | 0 | 0.002910±0.009651 | 0.002251±0.006754 | 0 | 0 | 0 |
| *Alcaligenes_faecalis* | 0 | 0 | 0.002186±0.007250 | 0 | 0 | 0 | 0 | 0 |
| *Alcanivorax_venustensis* | 0 | 0.002678±0.008882 | 0 | 0 | 0 | 0 | 0 | 0 |
| *Aliidiomarina_maris* | 0.001695±0.005084 | 0 | 0.002270±0.007528 | 0.004640±0.010554 | 0.002129±0.004234 | 0.008171±0.016427 | 0 | 0.018734±0.029971 |
| *Aliidiomarina_shirensis* | 0 | 0 | 0 | 0 | 0 | 0 | 0 | 0.002620±0.005239 |
| *Alistipes_finegoldii* | 0 | 0 | 0.005402±0.010287 | 0.002553±0.008467 | 0 | 0 | 0.001356±0.003032 | 0 |
| *Alistipes_indistinctus* | 0 | 0 | 0 | 0 | 0 | 0 | 0 | 0.004077±0.008154 |
| *Alistipes_onderdonkii* | 0 | 0 | 0 | 0.001876±0.006221 | 0 | 0 | 0 | 0.002620±0.005239 |
| *Alistipes_putredinis* | 0.007832±0.023495 | 0 | 0 | 0.002553±0.008467 | 0 | 0 | 0 | 0 |
| *Alistipes_shahii* | 0 | 0 | 0 | 0 | 0 | 0 | 0.025543±0.057115 | 0 |
| *Alkalibacterium_iburiense* | 0.001666±0.004999 | 0 | 0.000757±0.002509 | 0.005960±0.010523 | 0.003971±0.009035 | 0.011622±0.014045 | 0 | 0 |
| *Alkalibacterium_olivapovliticus* | 0 | 0 | 0 | 0 | 0 | 0.003239±0.009718 | 0 | 0.002620±0.005239 |
| *Alkalibaculum_bacchi* | 0 | 0 | 0 | 0.005463±0.012186 | 0.002968±0.008903 | 0 | 0 | 0 |
| *Alkanindiges_illinoisensis* | 0.002158±0.006475 | 0.000669±0.002220 | 0.001124±0.003727 | 0.001112±0.003687 | 0.002158±0.006474 | 0.005586±0.011273 | 0 | 0.006031±0.012061 |
| *Alloiococcus_otitis* | 0 | 0.000393±0.001304 | 0 | 0 | 0 | 0 | 0 | 0 |
| *Alloprevotella_rava* | 0 | 0.005083±0.016858 | 0.002802±0.004884 | 0.005570±0.011157 | 0 | 0.005993±0.011947 | 0 | 0 |
| *Altererythrobacter_epoxidivorans* | 0 | 0 | 0 | 0 | 0.001126±0.003377 | 0 | 0 | 0 |
| *Amaricoccus_kaplicensis* | 0 | 0 | 0 | 0.001531±0.005079 | 0 | 0 | 0 | 0 |
| *Aminobacter_niigataensis* | 0.004215±0.008392 | 0.011984±0.027743 | 0 | 0 | 0 | 0 | 0 | 0 |
| *Amphibacillus_xylanus* | 0 | 0 | 0.001093±0.003625 | 0 | 0 | 0 | 0 | 0 |
| *Anaerococcus_octavius* | 0 | 0 | 0.012445±0.025125 | 0.011352±0.026149 | 0.002635±0.005300 | 0.003239±0.009718 | 0 | 0 |
| *Anaerococcus_prevotii* | 0 | 0 | 0 | 0.003063±0.010159 | 0 | 0.006088±0.018265 | 0.007154±0.015997 | 0 |
| *Anaerococcus_vaginalis* | 0 | 0 | 0 | 0.001531±0.005079 | 0 | 0 | 0 | 0 |
| *Anaerofilum_agile* | 0 | 0 | 0.003783±0.012547 | 0 | 0 | 0 | 0 | 0 |
| *Anaerofilum_pentosovorans* | 0 | 0 | 0 | 0 | 0 | 0 | 0.002385±0.005332 | 0 |
| *Anaerofustis_stercorihominis* | 0 | 0 | 0.002459±0.008156 | 0 | 0.003102±0.009306 | 0 | 0 | 0 |
| *Anaeroplasma_abactoclasticum* | 0 | 0 | 0.003090±0.008215 | 0 | 0.003102±0.009306 | 0 | 0 | 0.003409±0.006818 |
| *Anaerovibrio_lipolyticus* | 0.001695±0.005084 | 0.003833±0.008989 | 0.002415±0.006073 | 0.003751±0.012441 | 0 | 0.002336±0.007009 | 0 | 0.006697±0.008091 |
| *Anaerovorax_odorimutans* | 0 | 0 | 0.006964±0.016275 | 0.001876±0.006221 | 0 | 0.003239±0.009718 | 0 | 0 |
| *Angiopteris_evecta* | 0 | 0 | 0.001513±0.005019 | 0 | 0 | 0 | 0 | 0 |
| *Anoxybacillus_flavithermus* | 0.001129±0.003386 | 0.005055±0.010484 | 0.006707±0.017863 | 0.000671±0.002225 | 0.060797±0.157164 | 0 | 0.168531±0.332343 | 0.016530±0.007572 |
| *Anoxybacillus_kamchatkensis* | 0 | 0 | 0 | 0 | 0.002007±0.006020 | 0 | 0.005447±0.012179 | 0 |
| *Anoxybacillus_kestanbolensis* | 0.083465±0.106860 | 0.085226±0.130348 | 0.075616±0.134326 | 0.029398±0.062011 | 0.379083±0.973520 | 0.055198±0.050798 | 1.381164±2.472079 | 0.360944±0.274596 |
| *Anoxybacillus_mongoliensis* | 0 | 0.003784±0.012550 | 0 | 0.001783±0.004111 | 0.003010±0.009030 | 0 | 0.013188±0.015308 | 0.011268±0.014903 |
| *Aquabacterium_citratiphilum* | 0 | 0.001240±0.002977 | 0 | 0.000671±0.002225 | 0 | 0 | 0 | 0 |
| *Aquabacterium_commune* | 0 | 0 | 0.004441±0.008212 | 0.001531±0.005079 | 0 | 0 | 0 | 0 |
| *Aquabacterium_parvum* | 0 | 0 | 0 | 0 | 0.001003±0.003010 | 0 | 0 | 0 |
| *Aquamicrobium_aerolatum* | 0 | 0 | 0 | 0 | 0.004316±0.012947 | 0 | 0 | 0.006818±0.013635 |
| *Aquincola_tertiaricarbonis* | 0.002158±0.006475 | 0 | 0 | 0 | 0 | 0 | 0 | 0 |
| *Aquitalea_denitrificans* | 0.001695±0.005084 | 0 | 0 | 0 | 0 | 0 | 0 | 0 |
| *Arcobacter_butzleri* | 0 | 0 | 0 | 0.003224±0.008537 | 0 | 0 | 0.001356±0.003032 | 0 |
| *Arcobacter_cryaerophilus* | 0 | 0.005016±0.011160 | 0.005799±0.010571 | 0 | 0 | 0 | 0.002385±0.005332 | 0.006818±0.013635 |
| *Arcobacter_venerupis* | 0 | 0 | 0 | 0 | 0.001126±0.003377 | 0 | 0 | 0.005239±0.010479 |
| *Armatimonas_rosea* | 0 | 0 | 0 | 0 | 0.003102±0.009306 | 0 | 0.004067±0.009095 | 0 |
| *Arthrobacter_agilis* | 0 | 0 | 0 | 0.003023±0.010027 | 0 | 0.002336±0.007009 | 0 | 0 |
| *Arthrobacter_antarcticus* | 0.003386±0.010158 | 0.003191±0.008436 | 0.004672±0.009345 | 0.001342±0.004450 | 0.003668±0.007422 | 0.005080±0.010121 | 0.001356±0.003032 | 0.009437±0.012874 |
| *Arthrobacter_arilaitensis* | 0.001695±0.005084 | 0 | 0.001124±0.003727 | 0 | 0 | 0 | 0 | 0.003409±0.006818 |
| *Arthrobacter_bergerei* | 0 | 0 | 0 | 0.001876±0.006221 | 0 | 0.001589±0.004766 | 0 | 0.002620±0.005239 |
| *Arthrobacter_citreus* | 0 | 0 | 0 | 0 | 0.003546±0.010639 | 0 | 0 | 0 |
| *Arthrobacter_psychrochitiniphilus* | 0.090892±0.088392 | 0.099783±0.097608 | 0.116869±0.074783 | 0.084702±0.054465 | 0.109427±0.077735 | 0.091605±0.073742 | 0.100337±0.088316 | 0.128981±0.098295 |
| *Arthrobacter_psychrolactophilus* | 0.004680±0.007236 | 0.003522±0.008684 | 0.004993±0.006179 | 0.001876±0.006221 | 0.002635±0.005300 | 0.003925±0.007968 | 0.002385±0.005332 | 0 |
| *Arthrobacter_ureafaciens* | 0 | 0 | 0.002459±0.008156 | 0 | 0 | 0 | 0.002385±0.005332 | 0 |
| *Atopostipes_suicloacalis* | 0.017469±0.031220 | 0.022437±0.027705 | 0.074003±0.084711 | 0.056862±0.089720 | 0.091241±0.169074 | 0.089126±0.158995 | 0.020996±0.014560 | 0.055747±0.039259 |
| *Aurantimonas_altamirensis* | 0 | 0 | 0 | 0.001112±0.003687 | 0 | 0 | 0 | 0 |
| *Azonexus_fungiphilus* | 0 | 0 | 0 | 0.000671±0.002225 | 0 | 0 | 0 | 0 |
| *Azospira_oryzae* | 0 | 0 | 0 | 0.008281±0.014706 | 0 | 0 | 0 | 0 |
| *Azospirillum_melinis* | 0 | 0 | 0.000631±0.002094 | 0 | 0 | 0 | 0 | 0 |
| *Azospirillum_zeae* | 0.002158±0.006475 | 0.002522±0.008364 | 0.007716±0.021394 | 0.001112±0.003687 | 0 | 0 | 0 | 0 |
| *Bacillus_aerophilus* | 0.195506±0.110898 | 0.185755±0.079210 | 0.190243±0.099721 | 0.184630±0.087972 | 0.197784±0.092379 | 0.175239±0.090932 | 0.173690±0.049430 | 0.155456±0.036231 |
| *Bacillus_akibai* | 0 | 0 | 0.025381±0.052135 | 0 | 0 | 0 | 0 | 0 |
| *Bacillus_amyloliquefaciens* | 0.004260±0.008707 | 0.002814±0.006838 | 0.005195±0.006396 | 0.007724±0.009169 | 0.001510±0.004529 | 0.009620±0.014545 | 0.002385±0.005332 | 0.002620±0.005239 |
| *Bacillus_anthracis* | 0 | 0 | 0 | 0 | 0.003546±0.010639 | 0 | 0 | 0 |
| *Bacillus_asahii* | 0 | 0 | 0.001513±0.005019 | 0 | 0 | 0 | 0 | 0 |
| *Bacillus_badius* | 0.026219±0.026456 | 0.029195±0.022126 | 0.038402±0.032237 | 0.016449±0.022389 | 0.026642±0.017706 | 0.025943±0.026652 | 0.025498±0.024974 | 0.029255±0.012212 |
| *Bacillus_beijingensis* | 0 | 0 | 0 | 0 | 0.001003±0.003010 | 0 | 0 | 0 |
| *Bacillus_cecembensis* | 0.002158±0.006475 | 0.008073±0.017575 | 0 | 0 | 0 | 0.003498±0.010495 | 0.002711±0.006063 | 0 |
| *Bacillus_cereus* | 30.281248±3.343709 | 27.594485±3.800226 | 28.120072±2.724867 | 28.254224±4.131474 | 28.653634±2.737460 | 30.488485±2.903849 | 28.160779±4.224776 | 27.772799±0.746478 |
| *Bacillus_circulans* | 0.085796±0.038977 | 0.061457±0.044585 | 0.081599±0.026698 | 0.105359±0.078916 | 0.106664±0.058592 | 0.075476±0.029127 | 0.092627±0.054656 | 0.051399±0.022544 |
| *Bacillus_coagulans* | 0 | 0 | 0.000631±0.002094 | 0 | 0 | 0 | 0 | 0 |
| *Bacillus_cohnii* | 0 | 0.000669±0.002220 | 0.001124±0.003727 | 0 | 0 | 0 | 0 | 0.002620±0.005239 |
| *Bacillus_firmus* | 0.069892±0.050348 | 0.043529±0.033700 | 0.063567±0.028208 | 0.070631±0.026289 | 0.048427±0.039754 | 0.038288±0.026195 | 0.049049±0.030676 | 0.039857±0.017709 |
| *Bacillus_flexus* | 29.129131±2.760883 | 26.875668±2.401885 | 27.344672±2.092008 | 27.458755±3.992856 | 27.833594±3.400088 | 28.920620±2.493359 | 28.211273±4.133733 | 27.704992±1.327736 |
| *Bacillus_ginsengi* | 0 | 0 | 0.000631±0.002094 | 0 | 0 | 0 | 0 | 0 |
| *Bacillus_gottheilii* | 0 | 0 | 0 | 0 | 0.002968±0.008903 | 0 | 0 | 0 |
| *Bacillus_horikoshii* | 0.004680±0.007236 | 0.016158±0.019832 | 0.003807±0.006550 | 0.011290±0.020069 | 0.017655±0.032156 | 0.001589±0.004766 | 0 | 0.002620±0.005239 |
| *Bacillus_idriensis* | 0 | 0.003495±0.008599 | 0.000922±0.003058 | 0.004594±0.008096 | 0 | 0.006427±0.012755 | 0.003740±0.005435 | 0 |
| *Bacillus_infantis* | 0.005547±0.011351 | 0.019112±0.023373 | 0.004294±0.007589 | 0.008840±0.018667 | 0.002635±0.005300 | 0.006489±0.012877 | 0.007808±0.011964 | 0.009712±0.006927 |
| *Bacillus_kochii* | 0.014714±0.019877 | 0.016091±0.019431 | 0.017780±0.026506 | 0.013768±0.017595 | 0.010784±0.013559 | 0.009667±0.014501 | 0.014232±0.016818 | 0.003015±0.006031 |
| *Bacillus_koreensis* | 0.005055±0.010754 | 0.005839±0.013670 | 0.002705±0.006381 | 0 | 0.003971±0.009035 | 0 | 0 | 0 |
| *Bacillus_licheniformis* | 0.010407±0.017812 | 0.007011±0.010089 | 0.017481±0.026733 | 0.011448±0.015030 | 0.015968±0.018872 | 0.021658±0.037453 | 0.016049±0.013452 | 0.009044±0.006165 |
| *Bacillus_massiliensis* | 0.001695±0.005084 | 0.002001±0.006637 | 0.008551±0.014263 | 0.004022±0.009981 | 0.001510±0.004529 | 0 | 0 | 0.003015±0.006031 |
| *Bacillus_megaterium* | 0.114456±0.036089 | 0.140315±0.055312 | 0.077597±0.044409 | 0.112755±0.054964 | 0.115350±0.054313 | 0.116929±0.064334 | 0.110398±0.041548 | 0.189780±0.041450 |
| *Bacillus_muralis* | 0 | 0.001001±0.003318 | 0 | 0 | 0 | 0 | 0.001356±0.003032 | 0 |
| *Bacillus_mycoides* | 0.003770±0.011311 | 0.009003±0.015163 | 0.007133±0.023657 | 0.006046±0.020054 | 0 | 0 | 0 | 0.004077±0.008154 |
| *Bacillus_niabensis* | 0.003404±0.010211 | 0 | 0 | 0 | 0.002968±0.008903 | 0.003378±0.010135 | 0.004217±0.006358 | 0.010501±0.007217 |
| *Bacillus_oceanisediminis* | 0.091829±0.076775 | 0.094210±0.073987 | 0.131024±0.073601 | 0.071034±0.064782 | 0.095900±0.072088 | 0.153322±0.190250 | 0.166232±0.142894 | 0.184290±0.115186 |
| *Bacillus_odysseyi* | 0.001885±0.005655 | 0.003286±0.006740 | 0 | 0 | 0 | 0 | 0 | 0 |
| *Bacillus_oleronius* | 0 | 0.001586±0.004041 | 0.003082±0.005579 | 0.005354±0.011929 | 0.003392±0.010175 | 0 | 0.002385±0.005332 | 0 |
| *Bacillus_pseudofirmus* | 0.010441±0.015596 | 0.010920±0.019270 | 0.013107±0.015247 | 0.005000±0.008890 | 0.015234±0.024542 | 0.011710±0.021731 | 0.001356±0.003032 | 0 |
| *Bacillus_pseudomycoides* | 0.006955±0.011268 | 0.003526±0.006768 | 0.002532±0.006442 | 0.014299±0.022005 | 0.012006±0.021910 | 0.003498±0.010495 | 0.008584±0.019194 | 0.004077±0.008154 |
| *Bacillus_psychrodurans* | 0 | 0 | 0.001093±0.003625 | 0 | 0.001003±0.003010 | 0 | 0 | 0 |
| *Bacillus_psychrosaccharolyticus* | 0 | 0 | 0.001844±0.006115 | 0 | 0.007093±0.021279 | 0 | 0 | 0 |
| *Bacillus_pumilus* | 2.581173±1.146327 | 2.778633±1.147668 | 2.851043±0.914867 | 2.349688±0.922444 | 2.404833±0.891024 | 2.544713±1.157972 | 2.848238±1.079511 | 2.883906±1.158600 |
| *Bacillus_safensis* | 2.378206±0.937148 | 2.856433±1.451869 | 2.849859±0.973891 | 2.435604±0.644761 | 2.361481±0.928416 | 2.302669±0.647000 | 2.981603±1.471289 | 2.786582±0.915884 |
| *Bacillus_samanii* | 0 | 0.001892±0.006275 | 0.001783±0.005914 | 0 | 0 | 0 | 0.005447±0.012179 | 0.008154±0.016308 |
| *Bacillus_selenatarsenatis* | 0 | 0 | 0.000757±0.002509 | 0 | 0 | 0 | 0.002711±0.006063 | 0 |
| *Bacillus_shackletonii* | 0 | 0 | 0.000631±0.002094 | 0.001112±0.003687 | 0 | 0 | 0 | 0 |
| *Bacillus_simplex* | 0.002594±0.007781 | 0.009833±0.010830 | 0.004084±0.013545 | 0.001531±0.005079 | 0.006182±0.010966 | 0.015871±0.021613 | 0 | 0.003409±0.006818 |
| *Bacillus_subtilis* | 0.012464±0.013886 | 0.017674±0.021683 | 0.018994±0.017062 | 0.018360±0.019313 | 0.031929±0.022924 | 0.016450±0.013288 | 0.011169±0.015302 | 0.026908±0.020243 |
| *Bacillus_thuringiensis* | 0.290429±0.165856 | 0.257710±0.253701 | 0.311570±0.241890 | 0.308209±0.211352 | 0.271921±0.161284 | 0.257711±0.125719 | 0.142895±0.124000 | 0.249424±0.250259 |
| *Bacteroides_caccae* | 0 | 0 | 0.002450±0.005473 | 0 | 0 | 0 | 0 | 0 |
| *Bacteroides_coprocola* | 0 | 0.001694±0.005619 | 0.004384±0.008087 | 0.002546±0.006394 | 0 | 0 | 0 | 0.002620±0.005239 |
| *Bacteroides_dorei* | 0 | 0.000847±0.002810 | 0.003216±0.008290 | 0.002553±0.008467 | 0.022839±0.064805 | 0.003239±0.009718 | 0 | 0 |
| *Bacteroides_finegoldii* | 0.005874±0.017621 | 0 | 0 | 0 | 0 | 0 | 0 | 0 |
| *Bacteroides_fragilis* | 0 | 0.005212±0.010107 | 0.009661±0.016523 | 0.004428±0.009993 | 0.002007±0.006020 | 0 | 0.018587±0.027450 | 0 |
| *Bacteroides_ovatus* | 0 | 0 | 0 | 0 | 0 | 0 | 0.002385±0.005332 | 0 |
| *Bacteroides_paurosaccharolyticus* | 0 | 0 | 0.001124±0.003727 | 0 | 0 | 0 | 0 | 0 |
| *Bacteroides_plebeius* | 0 | 0 | 0 | 0 | 0.006204±0.018612 | 0.003239±0.009718 | 0 | 0.006029±0.007079 |
| *Bacteroides_stercoris* | 0.003916±0.011747 | 0 | 0 | 0 | 0 | 0.002336±0.007009 | 0.002385±0.005332 | 0 |
| *Bacteroides_thetaiotaomicron* | 0 | 0 | 0.007481±0.017534 | 0 | 0.009306±0.027917 | 0 | 0 | 0.002620±0.005239 |
| *Bacteroides_uniformis* | 0 | 0.000669±0.002220 | 0 | 0 | 0 | 0 | 0.012771±0.028558 | 0 |
| *Bacteroides_xylanisolvens* | 0 | 0 | 0.002270±0.007528 | 0 | 0 | 0 | 0 | 0 |
| *Barnesiella_intestinihominis* | 0.002257±0.006772 | 0.005059±0.011328 | 0.015708±0.028955 | 0.003023±0.010027 | 0.012090±0.016179 | 0.002743±0.008230 | 0.002711±0.006063 | 0.011936±0.015103 |
| *Bdellovibrio_bacteriovorus* | 0 | 0 | 0 | 0 | 0 | 0 | 0.001356±0.003032 | 0 |
| *Bhargavaea_cecembensis* | 0 | 0 | 0.001124±0.003727 | 0 | 0 | 0.003044±0.009132 | 0 | 0 |
| *Bifidobacterium_animalis* | 0 | 0 | 0 | 0 | 0 | 0 | 0 | 0.003409±0.006818 |
| *Blastocatella_fastidiosa* | 0.017745±0.035049 | 0.001670±0.003804 | 0.006971±0.011097 | 0.003751±0.012441 | 0.004503±0.013508 | 0.006355±0.019064 | 0 | 0 |
| *Blautia_glucerasea* | 0 | 0 | 0.001093±0.003625 | 0 | 0.003102±0.009306 | 0 | 0 | 0 |
| *Blautia_obeum* | 0 | 0 | 0 | 0 | 0 | 0 | 0.012771±0.028558 | 0 |
| *Blautia_schinkii* | 0 | 0.000669±0.002220 | 0.002247±0.007454 | 0 | 0 | 0 | 0 | 0 |
| *Bosea_genosp.* | 0.003287±0.006922 | 0.003308±0.008508 | 0 | 0 | 0 | 0 | 0 | 0.002620±0.005239 |
| *Brevibacillus_agri* | 0 | 0 | 0 | 0 | 0.001003±0.003010 | 0 | 0 | 0 |
| *Brevibacillus_brevis* | 0.001666±0.004999 | 0.002055±0.006817 | 0 | 0.003470±0.007746 | 0 | 0.003498±0.010495 | 0 | 0 |
| *Brevibacillus_invocatus* | 0 | 0.002285±0.006280 | 0.005985±0.014358 | 0.005226±0.010695 | 0.001003±0.003010 | 0.002743±0.008230 | 0.006386±0.014279 | 0 |
| *Brevibacillus_panacihumi* | 0.004143±0.008263 | 0.000393±0.001304 | 0.009201±0.015249 | 0.003355±0.011126 | 0.005261±0.010579 | 0 | 0.007741±0.013836 | 0.009437±0.012874 |
| *Brevibacillus_parabrevis* | 0 | 0.002055±0.006817 | 0 | 0 | 0 | 0 | 0 | 0 |
| *Brevibacillus_reuszeri* | 0.053308±0.075409 | 0.045239±0.056475 | 0.064867±0.084372 | 0.055705±0.109137 | 0.025203±0.032819 | 0.005087±0.010971 | 0.092897±0.166031 | 0.068544±0.050738 |
| *Brevibacterium_epidermidis* | 0 | 0.001001±0.003318 | 0 | 0 | 0 | 0 | 0 | 0 |
| *Brevundimonas_alba* | 0.001958±0.005874 | 0 | 0 | 0 | 0 | 0 | 0 | 0 |
| *Brevundimonas_aurantiaca* | 0 | 0.002522±0.008364 | 0 | 0 | 0 | 0 | 0 | 0.003409±0.006818 |
| *Brevundimonas_aveniformis* | 0.001702±0.005105 | 0 | 0 | 0 | 0 | 0 | 0 | 0 |
| *Brevundimonas_bullata* | 0.007720±0.015457 | 0.012860±0.012708 | 0.009038±0.008593 | 0.009004±0.016008 | 0.007247±0.011978 | 0.007164±0.011372 | 0.005096±0.007002 | 0.005239±0.010479 |
| *Brevundimonas_diminuta* | 0 | 0.008820±0.019197 | 0.002459±0.008156 | 0.001112±0.003687 | 0 | 0 | 0 | 0 |
| *Brevundimonas_nasdae* | 0.001695±0.005084 | 0.007720±0.018296 | 0 | 0.002765±0.009170 | 0 | 0 | 0 | 0 |
| *Brevundimonas_staleyi* | 0 | 0 | 0 | 0 | 0 | 0 | 0 | 0.003015±0.006031 |
| *Brevundimonas_terrae* | 0 | 0 | 0.001093±0.003625 | 0 | 0 | 0 | 0 | 0 |
| *Brevundimonas_vesicularis* | 0.005045±0.011812 | 0.013419±0.024907 | 0.001326±0.004398 | 0.005777±0.016858 | 0 | 0 | 0 | 0 |
| *Brochothrix_thermosphacta* | 0.745858±0.256549 | 0.733659±0.384864 | 0.835877±0.254348 | 0.753327±0.200200 | 0.685542±0.171964 | 0.705446±0.289264 | 0.638536±0.271354 | 0.799866±0.157428 |
| *Brucella_melitensis* | 0.001695±0.005084 | 0.001694±0.005619 | 0.003713±0.006393 | 0 | 0.006474±0.019421 | 0 | 0 | 0 |
| *Bryobacter_aggregatus* | 0 | 0 | 0 | 0.000671±0.002225 | 0.003102±0.009306 | 0 | 0.019157±0.042837 | 0 |
| *Budvicia_aquatica* | 0 | 0 | 0 | 0 | 0.001510±0.004529 | 0 | 0 | 0 |
| *Burkholderia_lata* | 0 | 0.001193±0.003957 | 0 | 0 | 0 | 0 | 0 | 0 |
| *Buttiauxella_agrestis* | 0 | 0 | 0 | 0.000671±0.002225 | 0 | 0.001589±0.004766 | 0.002385±0.005332 | 0 |
| *Buttiauxella_brennerae* | 0 | 0 | 0.000922±0.003058 | 0 | 0 | 0 | 0 | 0 |
| *Buttiauxella_noackiae* | 0 | 0 | 0 | 0 | 0 | 0.013358±0.025248 | 0 | 0 |
| *Butyricicoccus_pullicaecorum* | 0 | 0.000669±0.002220 | 0.002652±0.008797 | 0.001876±0.006221 | 0.003102±0.009306 | 0 | 0 | 0 |
| *Butyrivibrio_crossotus* | 0.007277±0.012364 | 0.001001±0.003318 | 0.000757±0.002509 | 0.001876±0.006221 | 0 | 0.012958±0.038873 | 0.012838±0.013703 | 0.002620±0.005239 |
| *Byssovorax_cruenta* | 0 | 0 | 0.001844±0.006115 | 0 | 0 | 0 | 0 | 0 |
| *Campylobacter_gracilis* | 0 | 0 | 0 | 0 | 0.003102±0.009306 | 0 | 0 | 0 |
| *Campylobacter_hyointestinalis* | 0.003652±0.007272 | 0 | 0.002270±0.007528 | 0 | 0.006783±0.020350 | 0 | 0 | 0 |
| *Campylobacter_ureolyticus* | 0 | 0 | 0 | 0 | 0 | 0 | 0.006386±0.014279 | 0 |
| *Capnocytophaga_granulosa* | 0 | 0 | 0 | 0.003751±0.012441 | 0 | 0 | 0 | 0 |
| *Carnobacterium_gallinarum* | 0.001129±0.003386 | 0 | 0 | 0 | 0 | 0 | 0 | 0 |
| *Carnobacterium_inhibens* | 0.001702±0.005105 | 0 | 0 | 0 | 0 | 0 | 0 | 0 |
| *Carnobacterium_jeotgali* | 0 | 0 | 0 | 0 | 0 | 0 | 0.006386±0.014279 | 0 |
| *Carnobacterium_maltaromaticum* | 0.003333±0.009998 | 0.002364±0.005832 | 0.002540±0.006189 | 0 | 0.003971±0.009035 | 0.007423±0.011948 | 0.005573±0.007635 | 0.004077±0.008154 |
| *Carnobacterium_mobile* | 0.008509±0.025527 | 0 | 0 | 0 | 0 | 0 | 0 | 0 |
| *Carnobacterium_viridans* | 0 | 0.004109±0.009796 | 0.001326±0.004398 | 0.007183±0.012804 | 0 | 0 | 0 | 0.003409±0.006818 |
| *Caryophanon_latum* | 0.001695±0.005084 | 0 | 0.001513±0.005019 | 0 | 0.017732±0.053197 | 0.003239±0.009718 | 0 | 0.002620±0.005239 |
| *Caryophanon_tenue* | 0 | 0 | 0 | 0.005933±0.013204 | 0.002158±0.006474 | 0.006284±0.012476 | 0 | 0.003015±0.006031 |
| *Caulobacter_leidyi* | 0 | 0 | 0 | 0 | 0 | 0.001589±0.004766 | 0 | 0 |
| *Caulobacter_vibrioides* | 0.001695±0.005084 | 0.002449±0.006811 | 0.002459±0.008156 | 0.001531±0.005079 | 0 | 0 | 0.006386±0.014279 | 0 |
| *Cellulosilyticum_ruminicola* | 0 | 0 | 0.004390±0.007710 | 0.010358±0.018886 | 0.010935±0.012665 | 0.006088±0.018265 | 0.007481±0.010870 | 0.013099±0.026197 |
| *Cellvibrio_ostraviensis* | 0 | 0 | 0 | 0 | 0.002158±0.006474 | 0 | 0 | 0 |
| *Chromohalobacter_beijerinckii* | 0 | 0 | 0.000757±0.002509 | 0 | 0 | 0 | 0 | 0 |
| *Chromohalobacter_canadensis* | 0 | 0.000847±0.002810 | 0 | 0 | 0 | 0 | 0 | 0.003015±0.006031 |
| *Chryseobacterium_antarcticum* | 0 | 0.002522±0.008364 | 0 | 0.002553±0.008467 | 0 | 0 | 0 | 0 |
| *Chryseobacterium_bovis* | 0.002831±0.005763 | 0 | 0.006511±0.011677 | 0.001342±0.004450 | 0.003546±0.010639 | 0.003239±0.009718 | 0 | 0.003409±0.006818 |
| *Chryseobacterium_chaponense* | 0 | 0 | 0.000922±0.003058 | 0 | 0.005935±0.017806 | 0 | 0 | 0.002620±0.005239 |
| *Chryseobacterium_greenlandense* | 0.003386±0.010158 | 0.011005±0.019605 | 0.000631±0.002094 | 0 | 0 | 0 | 0 | 0 |
| *Chryseobacterium_haifense* | 0 | 0 | 0.001093±0.003625 | 0 | 0.001510±0.004529 | 0 | 0 | 0 |
| *Chryseobacterium_hominis* | 0 | 0.002008±0.006661 | 0 | 0.001863±0.006180 | 0 | 0 | 0 | 0.009046±0.018092 |
| *Chryseobacterium_piscicola* | 0.003916±0.011747 | 0 | 0 | 0.001531±0.005079 | 0 | 0 | 0 | 0 |
| *Chryseobacterium_piscium* | 0.004989±0.007835 | 0.000847±0.002810 | 0.007258±0.016239 | 0.001876±0.006221 | 0.003546±0.010639 | 0.006479±0.019436 | 0.017280±0.021488 | 0 |
| *Chryseobacterium_vrystaatense* | 0.001129±0.003386 | 0 | 0.001093±0.003625 | 0.001876±0.006221 | 0 | 0 | 0.002385±0.005332 | 0.006818±0.013635 |
| *Chryseomicrobium_amylolyticum* | 0 | 0 | 0 | 0.001863±0.006180 | 0 | 0 | 0 | 0 |
| *Chryseomicrobium_imtechense* | 0 | 0.000847±0.002810 | 0 | 0.001876±0.006221 | 0 | 0 | 0 | 0 |
| *Chryseomicrobium_imtechense_* | 0 | 0.000669±0.002220 | 0.001357±0.004502 | 0.003751±0.012441 | 0 | 0 | 0 | 0 |
| *Citrobacter_braakii* | 0 | 0 | 0 | 0 | 0 | 0.002743±0.008230 | 0 | 0 |
| *Citrobacter_gillenii* | 0 | 0 | 0 | 0 | 0 | 0.002743±0.008230 | 0 | 0 |
| *Citrobacter_youngae* | 0 | 0 | 0 | 0 | 0 | 0.002743±0.008230 | 0 | 0 |
| *Clostridium_bartlettii* | 0 | 0.001001±0.003318 | 0.004242±0.009584 | 0.001112±0.003687 | 0 | 0.003498±0.010495 | 0 | 0 |
| *Clostridium_bolteae* | 0.003916±0.011747 | 0 | 0 | 0 | 0 | 0 | 0 | 0 |
| *Clostridium_butyricum* | 0 | 0 | 0 | 0.001607±0.005329 | 0.003102±0.009306 | 0 | 0 | 0 |
| *Clostridium_clostridioforme* | 0 | 0 | 0.000922±0.003058 | 0 | 0.006204±0.018612 | 0 | 0 | 0 |
| *Clostridium_cocleatum* | 0 | 0.001001±0.003318 | 0.002270±0.007528 | 0 | 0 | 0 | 0.002711±0.006063 | 0 |
| *Clostridium_disporicum* | 0.008927±0.015710 | 0.030737±0.030271 | 0.081639±0.172917 | 0.065152±0.074482 | 0.038541±0.057452 | 0.049648±0.079657 | 0.040528±0.052713 | 0.033826±0.022763 |
| *Clostridium_glycolicum* | 0 | 0.005969±0.019797 | 0 | 0.000671±0.002225 | 0.003102±0.009306 | 0 | 0 | 0 |
| *Clostridium_hastiforme* | 0 | 0 | 0.001093±0.003625 | 0 | 0.007132±0.010948 | 0 | 0 | 0 |
| *Clostridium_hathewayi* | 0 | 0 | 0 | 0 | 0 | 0 | 0.002711±0.006063 | 0 |
| *Clostridium_jejuense* | 0 | 0 | 0 | 0 | 0 | 0.003044±0.009132 | 0 | 0 |
| *Clostridium_kluyveri* | 0 | 0 | 0.003027±0.010038 | 0 | 0 | 0 | 0 | 0 |
| *Clostridium_lactatifermentans* | 0 | 0.002186±0.005012 | 0.012351±0.020686 | 0.007871±0.018434 | 0.015510±0.046529 | 0.006284±0.012476 | 0 | 0.004077±0.008154 |
| *Clostridium_leptum* | 0 | 0.001339±0.004441 | 0.003139±0.005402 | 0.001876±0.006221 | 0.021714±0.065141 | 0.002743±0.008230 | 0 | 0 |
| *Clostridium_lituseburense* | 0.014781±0.019207 | 0.019043±0.026089 | 0.075380±0.147621 | 0.070767±0.118851 | 0.055615±0.056886 | 0.041191±0.048933 | 0.015312±0.016067 | 0.104768±0.049278 |
| *Clostridium_methylpentosum* | 0 | 0 | 0 | 0.003751±0.012441 | 0 | 0 | 0 | 0 |
| *Clostridium_perfringens* | 0.007126±0.011882 | 0.005881±0.010873 | 0.004564±0.006735 | 0.001531±0.005079 | 0.008672±0.013381 | 0 | 0.008770±0.013937 | 0 |
| *Clostridium_polysaccharolyticum* | 0 | 0.000669±0.002220 | 0 | 0 | 0 | 0 | 0 | 0 |
| *Clostridium_ramosum* | 0 | 0 | 0.001679±0.003757 | 0 | 0.003377±0.010131 | 0 | 0.005423±0.012126 | 0 |
| *Clostridium_ruminantium* | 0 | 0.000393±0.001304 | 0 | 0 | 0 | 0 | 0 | 0 |
| *Clostridium_saccharogumia* | 0 | 0 | 0 | 0 | 0 | 0.002336±0.007009 | 0 | 0 |
| *Clostridium_scindens* | 0 | 0 | 0 | 0 | 0.001003±0.003010 | 0 | 0 | 0 |
| *Clostridium_straminisolvens* | 0 | 0 | 0 | 0.003023±0.010027 | 0 | 0 | 0 | 0 |
| *Clostridium_tagluense* | 0 | 0 | 0 | 0 | 0 | 0 | 0.001356±0.003032 | 0 |
| *Clostridium_tyrobutyricum* | 0.001958±0.005874 | 0 | 0 | 0 | 0 | 0 | 0 | 0 |
| *Comamonas_jiangduensis* | 0.005618±0.012210 | 0.007038±0.010731 | 0.001844±0.006115 | 0.007123±0.011211 | 0.012797±0.031765 | 0 | 0.002385±0.005332 | 0.008650±0.011437 |
| *Comamonas_koreensis* | 0.005526±0.008381 | 0.002710±0.004822 | 0.016236±0.019597 | 0.002643±0.005971 | 0.016430±0.036845 | 0.007423±0.011948 | 0.001356±0.003032 | 0 |
| *Comamonas_terrae* | 0 | 0 | 0 | 0.002910±0.009651 | 0 | 0 | 0 | 0 |
| *Comamonas_testosteroni* | 0 | 0 | 0 | 0 | 0.001510±0.004529 | 0.003539±0.010616 | 0 | 0 |
| *Coprobacillus_cateniformis* | 0 | 0 | 0 | 0 | 0.006204±0.018612 | 0 | 0 | 0 |
| *Coprococcus_catus* | 0 | 0 | 0.000922±0.003058 | 0 | 0 | 0 | 0 | 0 |
| *Coprococcus_comes* | 0 | 0 | 0.000631±0.002094 | 0.001531±0.005079 | 0 | 0 | 0.019157±0.042837 | 0 |
| *Coprococcus_eutactus* | 0 | 0 | 0.001093±0.003625 | 0 | 0.001126±0.003377 | 0 | 0 | 0 |
| *Corynebacterium_accolens* | 0 | 0 | 0.002270±0.007528 | 0.000671±0.002225 | 0 | 0 | 0 | 0 |
| *Corynebacterium_bovis* | 0.005187±0.015562 | 0.002001±0.006637 | 0.001880±0.004280 | 0 | 0.020785±0.034605 | 0.005127±0.011080 | 0 | 0.007859±0.015718 |
| *Corynebacterium_callunae* | 0 | 0 | 0.011224±0.019875 | 0.004428±0.009993 | 0.017084±0.032084 | 0.006284±0.012476 | 0.004769±0.010664 | 0.028589±0.011810 |
| *Corynebacterium_camporealensis* | 0 | 0 | 0 | 0.001531±0.005079 | 0 | 0 | 0 | 0 |
| *Corynebacterium_efficiens* | 0.001695±0.005084 | 0.004236±0.014049 | 0.028188±0.055971 | 0.021185±0.042342 | 0.070852±0.145507 | 0 | 0.014635±0.025822 | 0.031088±0.012249 |
| *Corynebacterium_glucuronolyticum* | 0 | 0 | 0 | 0.000671±0.002225 | 0 | 0.004633±0.009759 | 0 | 0 |
| *Corynebacterium_lipophiloflavum* | 0 | 0 | 0.000631±0.002094 | 0 | 0 | 0.003044±0.009132 | 0 | 0.003015±0.006031 |
| *Corynebacterium_macginleyi* | 0 | 0.001892±0.006275 | 0 | 0 | 0 | 0 | 0 | 0 |
| *Corynebacterium_marinum* | 0 | 0 | 0.002459±0.008156 | 0 | 0.002158±0.006474 | 0 | 0 | 0 |
| *Corynebacterium_maris* | 0 | 0 | 0 | 0.002802±0.009292 | 0 | 0.003539±0.010616 | 0 | 0 |
| *Corynebacterium_pilbarense* | 0 | 0 | 0 | 0 | 0 | 0.003239±0.009718 | 0 | 0 |
| *Corynebacterium_pilosum* | 0 | 0 | 0.000757±0.002509 | 0.001876±0.006221 | 0 | 0.003498±0.010495 | 0 | 0 |
| *Corynebacterium_suicordis* | 0 | 0 | 0 | 0.001531±0.005079 | 0 | 0 | 0 | 0 |
| *Corynebacterium_tuberculostearicum* | 0 | 0.002494±0.008272 | 0.002247±0.007454 | 0 | 0.001126±0.003377 | 0.006417±0.012734 | 0 | 0.002620±0.005239 |
| *Corynebacterium_tuscaniense* | 0 | 0 | 0 | 0 | 0 | 0.006479±0.019436 | 0 | 0 |
| *Cupriavidus_gilardii* | 0.002158±0.006475 | 0 | 0.007060±0.019001 | 0.001531±0.005079 | 0.002007±0.006020 | 0 | 0.001356±0.003032 | 0.006697±0.008091 |
| *Cystobacter_badius* | 0 | 0 | 0.001513±0.005019 | 0 | 0 | 0 | 0 | 0 |
| *Deinococcus_aetherius* | 0 | 0.002494±0.008272 | 0 | 0 | 0 | 0 | 0 | 0 |
| *Deinococcus_caeni* | 0 | 0 | 0 | 0 | 0 | 0 | 0.001356±0.003032 | 0 |
| *Deinococcus_daejeonensis* | 0.002257±0.006772 | 0 | 0.001783±0.005914 | 0.003895±0.009163 | 0 | 0.002743±0.008230 | 0 | 0.003015±0.006031 |
| *Deinococcus_ficus* | 0.001885±0.005655 | 0 | 0 | 0 | 0 | 0 | 0.001356±0.003032 | 0.002620±0.005239 |
| *Deinococcus_geothermalis* | 0.004788±0.007432 | 0.031599±0.062056 | 0.007380±0.014425 | 0.003895±0.009163 | 0.013823±0.026338 | 0.009501±0.015853 | 0.049518±0.068793 | 0 |
| *Deinococcus_grandis* | 0.018986±0.034877 | 0.001787±0.004011 | 0.032511±0.050829 | 0.043421±0.065897 | 0.025627±0.027197 | 0.025398±0.030334 | 0.039110±0.045637 | 0.095680±0.065002 |
| *Deinococcus_hopiensis* | 0 | 0 | 0.000631±0.002094 | 0 | 0 | 0 | 0 | 0 |
| *Deinococcus_murrayi* | 0 | 0 | 0 | 0.003023±0.010027 | 0 | 0 | 0.012771±0.028558 | 0 |
| *Delftia_lacustris* | 0.005881±0.009448 | 0.004782±0.011304 | 0.001388±0.003103 | 0.001531±0.005079 | 0.002007±0.006020 | 0 | 0 | 0.003409±0.006818 |
| *Desemzia_incerta* | 0.001695±0.005084 | 0.008364±0.022314 | 0.014159±0.033331 | 0.017770±0.023430 | 0.012796±0.032175 | 0.009587±0.019896 | 0 | 0.022732±0.028980 |
| *Desulfomicrobium_baculatum* | 0 | 0 | 0 | 0 | 0 | 0 | 0.001356±0.003032 | 0 |
| *Desulfonispora_thiosulfatigenes* | 0 | 0 | 0 | 0.003063±0.010159 | 0 | 0 | 0 | 0 |
| *Desulfovibrio_idahonensis* | 0 | 0.000393±0.001304 | 0.001513±0.005019 | 0 | 0 | 0 | 0 | 0 |
| *Devosia_limi* | 0 | 0 | 0 | 0.003407±0.007627 | 0 | 0 | 0 | 0 |
| *Devosia_riboflavina* | 0 | 0 | 0 | 0.001876±0.006221 | 0 | 0 | 0 | 0 |
| *Dietzia_cercidiphylli* | 0 | 0 | 0 | 0 | 0 | 0 | 0 | 0.002620±0.005239 |
| *Dietzia_maris* | 0 | 0 | 0 | 0 | 0 | 0.003498±0.010495 | 0 | 0 |
| *Dolosigranulum_pigrum* | 0 | 0.005356±0.017763 | 0 | 0 | 0 | 0 | 0 | 0 |
| *Dorea_longicatena* | 0 | 0.000847±0.002810 | 0.000631±0.002094 | 0 | 0 | 0 | 0 | 0.002620±0.005239 |
| *Duganella_zoogloeoides* | 0 | 0 | 0 | 0 | 0.001510±0.004529 | 0 | 0 | 0 |
| *Dysgonomonas_mossii* | 0.001129±0.003386 | 0 | 0 | 0 | 0 | 0 | 0 | 0 |
| *Empedobacter_brevis* | 0.002831±0.005763 | 0.000847±0.002810 | 0.000757±0.002509 | 0.000671±0.002225 | 0.003392±0.010175 | 0 | 0 | 0 |
| *Enhydrobacter_aerosaccus* | 0.118655±0.128519 | 0.119339±0.097564 | 0.160174±0.113037 | 0.132315±0.102724 | 0.112275±0.072620 | 0.100089±0.068996 | 0.273617±0.366618 | 0.351813±0.442256 |
| *Enterobacter_amnigenus* | 0 | 0 | 0 | 0.001876±0.006221 | 0 | 0 | 0 | 0 |
| *Enterobacter_asburiae* | 0 | 0 | 0 | 0 | 0 | 0 | 0.006386±0.014279 | 0 |
| *Enterobacter_cancerogenus* | 0 | 0 | 0 | 0.001342±0.004450 | 0 | 0 | 0 | 0 |
| *Enterobacter_cowanii* | 0 | 0 | 0 | 0 | 0 | 0 | 0 | 0.002620±0.005239 |
| *Enterobacter_hormaechei* | 0 | 0.002001±0.006637 | 0 | 0.001342±0.004450 | 0 | 0 | 0.001356±0.003032 | 0.002620±0.005239 |
| *Enterobacter_ludwigii* | 0.001958±0.005874 | 0 | 0 | 0.001342±0.004450 | 0 | 0 | 0.012771±0.028558 | 0.002620±0.005239 |
| *Enterococcus_aquimarinus* | 0 | 0 | 0.000757±0.002509 | 0 | 0.005045±0.010145 | 0.002336±0.007009 | 0 | 0.006029±0.007079 |
| *Enterococcus_durans* | 0.003087±0.006403 | 0 | 0.006543±0.015096 | 0 | 0.001126±0.003377 | 0 | 0 | 0.005239±0.010479 |
| *Enterococcus_faecalis* | 0.001129±0.003386 | 0.006203±0.017705 | 0.005717±0.010670 | 0 | 0 | 0 | 0.004067±0.009095 | 0.002620±0.005239 |
| *Enterococcus_faecium* | 0 | 0 | 0.004621±0.008505 | 0 | 0 | 0.003239±0.009718 | 0 | 0 |
| *Enterococcus_gallinarum* | 0 | 0 | 0 | 0.001342±0.004450 | 0.002251±0.006754 | 0 | 0 | 0 |
| *Enterococcus_hirae* | 0.002594±0.007781 | 0.000669±0.002220 | 0.001724±0.004001 | 0 | 0.008452±0.014588 | 0 | 0.001356±0.003032 | 0.002620±0.005239 |
| *Enterococcus_italicus* | 0 | 0 | 0 | 0 | 0 | 0 | 0 | 0.004077±0.008154 |
| *Enterococcus_sulfureus* | 0 | 0 | 0.001357±0.004502 | 0 | 0 | 0 | 0 | 0 |
| *Epilithonimonas_lactis* | 0 | 0.001001±0.003318 | 0.001513±0.005019 | 0.000671±0.002225 | 0 | 0 | 0 | 0 |
| *Erwinia_soli* | 0 | 0 | 0 | 0 | 0.001126±0.003377 | 0 | 0 | 0 |
| *Erysipelothrix_tonsillarum* | 0 | 0 | 0 | 0.002553±0.008467 | 0 | 0 | 0 | 0 |
| *Escherichia/Shigella_dysenteriae* | 0 | 0.020291±0.036068 | 0.138410±0.297307 | 0.009378±0.031103 | 0.040012±0.053392 | 0 | 0.044740±0.100041 | 0.002620±0.005239 |
| *Escherichia/Shigella_fergusonii* | 0 | 0.005850±0.016549 | 0.051566±0.151772 | 0 | 0.017270±0.022139 | 0.004766±0.014298 | 0.043384±0.097009 | 0 |
| *Escherichia/Shigella_flexneri* | 0 | 0.012238±0.023765 | 0.062088±0.125634 | 0.005627±0.018662 | 0.084089±0.140268 | 0.008005±0.016252 | 0.033894±0.075789 | 0.002620±0.005239 |
| *Escherichia/Shigella_vulneris* | 0 | 0.001848±0.004128 | 0.001093±0.003625 | 0 | 0 | 0 | 0 | 0 |
| *Escherichia_coli* | 0 | 0.049085±0.093310 | 0.123209±0.234590 | 0.010720±0.030976 | 0.138079±0.165692 | 0.026082±0.039185 | 0.061009±0.136420 | 0.011268±0.014903 |
| *Escherichia_vulneris* | 0 | 0 | 0.000631±0.002094 | 0 | 0.001510±0.004529 | 0 | 0.001356±0.003032 | 0 |
| *Eubacterium_coprostanoligenes* | 0.001885±0.005655 | 0.003735±0.008946 | 0.007967±0.013978 | 0.010554±0.021283 | 0.013533±0.036953 | 0.013448±0.019904 | 0.008770±0.013937 | 0.008255±0.010200 |
| *Eubacterium_desmolans* | 0 | 0.000847±0.002810 | 0 | 0 | 0 | 0 | 0 | 0 |
| *Eubacterium_eligens* | 0 | 0 | 0 | 0 | 0.003102±0.009306 | 0 | 0 | 0.006818±0.013635 |
| *Eubacterium_hallii* | 0 | 0 | 0.002459±0.008156 | 0 | 0.002158±0.006474 | 0.003239±0.009718 | 0.002711±0.006063 | 0 |
| *Eubacterium_rectale* | 0.007832±0.023495 | 0.001670±0.003804 | 0.005422±0.006460 | 0.005989±0.011720 | 0.003102±0.009306 | 0.009718±0.029155 | 0.053470±0.113016 | 0.020705±0.023912 |
| *Eubacterium_siraeum* | 0 | 0 | 0 | 0 | 0.009306±0.027917 | 0 | 0 | 0 |
| *Eubacterium_tenue* | 0.002795±0.005677 | 0.015850±0.023029 | 0.060115±0.080180 | 0.030652±0.038160 | 0.027482±0.038488 | 0.046693±0.089057 | 0.014634±0.022825 | 0.030296±0.023641 |
| *Eubacterium_tortuosum* | 0 | 0.001694±0.005619 | 0.006608±0.011401 | 0.000671±0.002225 | 0 | 0 | 0 | 0.011268±0.014903 |
| *Eubacterium_ventriosum* | 0.005874±0.017621 | 0 | 0 | 0 | 0 | 0 | 0 | 0 |
| *Eubacterium_xylanophilum* | 0 | 0.002008±0.006661 | 0.003979±0.013195 | 0 | 0.006204±0.018612 | 0 | 0 | 0 |
| *Ewingella_americana* | 0 | 0 | 0.001513±0.005019 | 0 | 0 | 0 | 0 | 0 |
| *Exiguobacterium_acetylicum* | 0 | 0 | 0 | 0 | 0 | 0 | 0 | 0.002620±0.005239 |
| *Exiguobacterium_aurantiacum* | 0 | 0.006018±0.011015 | 0.004084±0.013545 | 0.001112±0.003687 | 0 | 0 | 0 | 0 |
| *Exiguobacterium_indicum* | 0 | 0 | 0.000631±0.002094 | 0 | 0.003102±0.009306 | 0 | 0 | 0 |
| *Exiguobacterium_mexicanum* | 0 | 0 | 0 | 0.001112±0.003687 | 0 | 0 | 0 | 0 |
| *Facklamia_languida* | 0 | 0 | 0.004084±0.013545 | 0 | 0 | 0 | 0 | 0 |
| *Facklamia_tabacinasalis* | 0.008606±0.008529 | 0.006474±0.012385 | 0.017436±0.025888 | 0.037694±0.085919 | 0.025328±0.051725 | 0.059050±0.096495 | 0 | 0.041839±0.040281 |
| *Faecalibacterium_prausnitzii* | 0.013449±0.034973 | 0.004380±0.006214 | 0.002185±0.004932 | 0.006448±0.017073 | 0.002251±0.006754 | 0.004828±0.010275 | 0.025543±0.057115 | 0.016507±0.024773 |
| *Fibrobacter_intestinalis* | 0 | 0 | 0.002459±0.008156 | 0 | 0 | 0 | 0 | 0 |
| *Fimbriimonas_ginsengisoli* | 0 | 0 | 0 | 0 | 0 | 0 | 0 | 0.006818±0.013635 |
| *Finegoldia_magna* | 0 | 0 | 0 | 0.003734±0.010180 | 0 | 0 | 0.006386±0.014279 | 0 |
| *Flavisolibacter_ginsengiterrae* | 0 | 0 | 0.000922±0.003058 | 0 | 0 | 0 | 0 | 0 |
| *Flavisolibacter_sp._HY-50R* | 0 | 0 | 0 | 0 | 0 | 0.001589±0.004766 | 0 | 0 |
| *Flavobacterium_antarcticum* | 0 | 0 | 0.000631±0.002094 | 0 | 0 | 0 | 0 | 0 |
| *Flavobacterium_frigidarium* | 0.001695±0.005084 | 0.000847±0.002810 | 0.004012±0.008400 | 0 | 0.006182±0.010966 | 0.009740±0.021568 | 0.002711±0.006063 | 0.003015±0.006031 |
| *Flavobacterium_segetis* | 0 | 0 | 0.001263±0.004188 | 0 | 0.001003±0.003010 | 0 | 0.002385±0.005332 | 0.003409±0.006818 |
| *Flavobacterium_succinicans* | 0.021455±0.034584 | 0.028139±0.020036 | 0.027912±0.029278 | 0.006918±0.012593 | 0.005231±0.009469 | 0.010276±0.015432 | 0.007481±0.010870 | 0.023727±0.040768 |
| *Flavobacterium_ummariense* | 0 | 0.002695±0.006234 | 0.004309±0.008610 | 0 | 0 | 0.010460±0.016370 | 0.011832±0.016287 | 0.002620±0.005239 |
| *Flavonifractor_plautii* | 0.002158±0.006475 | 0.002008±0.006661 | 0.001093±0.003625 | 0.003751±0.012441 | 0 | 0 | 0 | 0 |
| *Fusobacterium_nucleatum* | 0.004515±0.013545 | 0.002494±0.008272 | 0.010141±0.033634 | 0 | 0.003102±0.009306 | 0 | 0 | 0.009437±0.012874 |
| *Gemella_haemolysans* | 0.001702±0.005105 | 0.000847±0.002810 | 0.000922±0.003058 | 0.000671±0.002225 | 0 | 0.003177±0.009532 | 0 | 0 |
| *Gemella_sanguinis* | 0 | 0 | 0.001093±0.003625 | 0 | 0 | 0 | 0 | 0 |
| *Geobacillus_stearothermophilus* | 0.001695±0.005084 | 0.000669±0.002220 | 0 | 0 | 0 | 0 | 0.001356±0.003032 | 0 |
| *Georgenia_satyanarayanai* | 0 | 0 | 0 | 0.001876±0.006221 | 0 | 0 | 0 | 0 |
| *Globicatella_sulfidifaciens* | 0 | 0 | 0 | 0 | 0 | 0 | 0.002385±0.005332 | 0 |
| *Glycine_max* | 0 | 0 | 0.002459±0.008156 | 0 | 0 | 0 | 0 | 0 |
| *Granulicatella_adiacens* | 0.003916±0.011747 | 0 | 0 | 0 | 0 | 0.003239±0.009718 | 0 | 0 |
| *Gulosibacter_molinativorax* | 0 | 0 | 0.002459±0.008156 | 0.001112±0.003687 | 0.001003±0.003010 | 0 | 0 | 0 |
| *Haemophilus_influenzae* | 0 | 0.005969±0.019797 | 0.000922±0.003058 | 0 | 0 | 0 | 0 | 0 |
| *Haemophilus_parainfluenzae* | 0.002823±0.005745 | 0 | 0.003258±0.007372 | 0 | 0.025260±0.064678 | 0.004672±0.014017 | 0.002385±0.005332 | 0.005635±0.006539 |
| *Haemophilus_paraphrohaemolyticus* | 0 | 0 | 0 | 0.001876±0.006221 | 0 | 0 | 0 | 0.003409±0.006818 |
| *Hafnia_alvei* | 0 | 0 | 0 | 0.001863±0.006180 | 0.003102±0.009306 | 0 | 0 | 0 |
| *Hafnia_paralvei* | 0.016166±0.023054 | 0.036373±0.047713 | 0.040059±0.046208 | 0.025861±0.022541 | 0.014038±0.015878 | 0.028979±0.052329 | 0.020058±0.028270 | 0.011664±0.008688 |
| *Halolactibacillus_halophilus* | 0 | 0 | 0 | 0 | 0.002158±0.006474 | 0 | 0 | 0 |
| *Halomonas_boliviensis* | 0 | 0 | 0 | 0.002553±0.008467 | 0 | 0 | 0 | 0.013099±0.026197 |
| *Halomonas_muralis* | 0 | 0 | 0.001388±0.003103 | 0 | 0 | 0 | 0 | 0 |
| *Halospirulina_tapeticola* | 0 | 0 | 0.005531±0.018346 | 0 | 0 | 0 | 0 | 0 |
| *Hansschlegelia_zhihuaiae* | 0.007039±0.011325 | 0.014595±0.029058 | 0 | 0 | 0 | 0 | 0 | 0 |
| *Helcococcus_ovis* | 0 | 0 | 0 | 0.001531±0.005079 | 0 | 0 | 0 | 0 |
| *Herbaspirillum_huttiense* | 0.001958±0.005874 | 0 | 0 | 0 | 0 | 0 | 0 | 0 |
| *Herbaspirillum_seropedicae* | 0.001129±0.003386 | 0.000393±0.001304 | 0 | 0 | 0 | 0 | 0 | 0 |
| *Howardella_ureilytica* | 0 | 0 | 0 | 0 | 0 | 0 | 0 | 0.005239±0.010479 |
| *Hydrogenophilus_hirschii* | 0 | 0 | 0 | 0.001876±0.006221 | 0 | 0 | 0 | 0 |
| *Hyphomicrobium_vulgare* | 0 | 0.000393±0.001304 | 0.001901±0.006305 | 0 | 0 | 0 | 0 | 0 |
| *Hyphomicrobium_zavarzinii* | 0.010389±0.020840 | 0.031595±0.055512 | 0 | 0 | 0 | 0 | 0 | 0 |
| *Ideonella_dechloratans* | 0.003624±0.007221 | 0 | 0.004372±0.014501 | 0 | 0.002251±0.006754 | 0 | 0 | 0.012455±0.017075 |
| *Ilumatobacter_fluminis* | 0 | 0 | 0.000922±0.003058 | 0 | 0 | 0 | 0 | 0 |
| *Insolitispirillum_peregrinum* | 0.001129±0.003386 | 0 | 0 | 0 | 0.002251±0.006754 | 0.006479±0.019436 | 0 | 0 |
| *Janibacter_limosus* | 0 | 0 | 0.000757±0.002509 | 0 | 0 | 0 | 0 | 0 |
| *Janthinobacterium_lividum* | 0.034894±0.044447 | 0.039539±0.052623 | 0.062107±0.057373 | 0.032274±0.033978 | 0.035659±0.039896 | 0.031598±0.032738 | 0.021188±0.027945 | 0.065944±0.050806 |
| *Jeotgalicoccus_psychrophilus* | 0.009545±0.016868 | 0.024500±0.044457 | 0.052955±0.068371 | 0.042923±0.048810 | 0.091733±0.126278 | 0.093807±0.093157 | 0.044513±0.054713 | 0.106801±0.087300 |
| *Kandleria_vitulina* | 0 | 0 | 0 | 0 | 0 | 0 | 0 | 0.002620±0.005239 |
| *Klebsiella_oxytoca* | 0 | 0.002001±0.006637 | 0.002435±0.005610 | 0 | 0 | 0 | 0.001356±0.003032 | 0 |
| *Klebsiella_pneumoniae* | 0.001129±0.003386 | 0.003525±0.009044 | 0.002637±0.005945 | 0 | 0 | 0 | 0 | 0 |
| *Kluyvera_cryocrescens* | 0 | 0 | 0 | 0 | 0 | 0.003539±0.010616 | 0 | 0 |
| *Kocuria_carniphila* | 0 | 0 | 0 | 0.002802±0.009292 | 0 | 0 | 0 | 0 |
| *Kocuria_rosea* | 0 | 0 | 0 | 0 | 0 | 0.003239±0.009718 | 0 | 0 |
| *Kurthia_gibsonii* | 0.001666±0.004999 | 0 | 0 | 0.002910±0.009651 | 0 | 0 | 0 | 0 |
| *Lachnoanaerobaculum_orale* | 0.002257±0.006772 | 0 | 0 | 0 | 0 | 0 | 0 | 0 |
| *Lactobacillus_acetotolerans* | 0.030225±0.025671 | 0.152103±0.283056 | 0.067543±0.082486 | 0.039621±0.035050 | 0.021782±0.031759 | 0.023802±0.033861 | 0.046101±0.051948 | 0.032940±0.018312 |
| *Lactobacillus_amylovorus* | 0 | 0 | 0 | 0.000671±0.002225 | 0 | 0 | 0 | 0 |
| *Lactobacillus_animalis* | 0 | 0.001001±0.003318 | 0.010609±0.035187 | 0 | 0.031019±0.093058 | 0.003239±0.009718 | 0.004067±0.009095 | 0 |
| *Lactobacillus_brevis* | 0.003624±0.007221 | 0.002040±0.004618 | 0.009120±0.016286 | 0.001863±0.006180 | 0.010431±0.027699 | 0.003239±0.009718 | 0.004067±0.009095 | 0.003015±0.006031 |
| *Lactobacillus_buchneri* | 0.005098±0.010823 | 0.014235±0.023478 | 0.007380±0.014425 | 0.001863±0.006180 | 0.002251±0.006754 | 0.002336±0.007009 | 0 | 0 |
| *Lactobacillus_delbrueckii* | 0.138166±0.321612 | 0.088937±0.143137 | 0.045291±0.040588 | 0.054459±0.109365 | 0.071772±0.193647 | 0.082717±0.221033 | 0.475434±1.019766 | 0.142862±0.122799 |
| *Lactobacillus_equi* | 0 | 0 | 0.001844±0.006115 | 0 | 0 | 0 | 0 | 0 |
| *Lactobacillus_frumenti* | 0 | 0 | 0 | 0 | 0 | 0.002336±0.007009 | 0 | 0 |
| *Lactobacillus_gallinarum* | 0 | 0 | 0.000922±0.003058 | 0 | 0 | 0 | 0 | 0 |
| *Lactobacillus_helveticus* | 0.021302±0.019555 | 0.042301±0.081939 | 0.011189±0.014937 | 0.022877±0.040158 | 0.022336±0.020504 | 0.026252±0.030102 | 0.047871±0.068493 | 0.017692±0.014438 |
| *Lactobacillus_iners* | 0.001129±0.003386 | 0 | 0 | 0.001531±0.005079 | 0 | 0 | 0 | 0 |
| *Lactobacillus_kisonensis* | 0 | 0.002678±0.008882 | 0.001679±0.003757 | 0 | 0 | 0 | 0 | 0 |
| *Lactobacillus_namurensis* | 0 | 0.008009±0.019659 | 0.007783±0.014856 | 0 | 0 | 0.003239±0.009718 | 0 | 0.003015±0.006031 |
| *Lactobacillus_panis* | 0 | 0 | 0.001093±0.003625 | 0 | 0 | 0 | 0 | 0 |
| *Lactobacillus_parabrevis* | 0.001958±0.005874 | 0.009069±0.027131 | 0 | 0 | 0 | 0 | 0 | 0.004077±0.008154 |
| *Lactobacillus_paracasei* | 0.055983±0.138223 | 0.016918±0.031242 | 0.010675±0.011794 | 0.011754±0.025560 | 0.034523±0.066250 | 0.046638±0.097938 | 0.209628±0.313529 | 0.041663±0.059280 |
| *Lactobacillus_parafarraginis* | 0 | 0.002541±0.008429 | 0.001388±0.003103 | 0 | 0.002251±0.006754 | 0 | 0 | 0 |
| *Lactobacillus_paraplantarum* | 0.051791±0.087947 | 0.015202±0.028696 | 0.087164±0.086288 | 0.012849±0.020618 | 0.037360±0.068888 | 0.003239±0.009718 | 0.006125±0.010332 | 0.018088±0.012331 |
| *Lactobacillus_pentosus* | 0.612256±0.956230 | 0.147730±0.132408 | 1.083629±0.996956 | 0.232033±0.441772 | 0.392263±0.578957 | 0.225425±0.531146 | 0.987200±2.038376 | 0.311302±0.329397 |
| *Lactobacillus_plantarum* | 0.503411±0.836821 | 0.127743±0.150909 | 0.654715±0.662242 | 0.221681±0.481506 | 0.385805±0.606506 | 0.239376±0.655448 | 1.295761±2.792542 | 0.258588±0.252555 |
| *Lactobacillus_pobuzihii* | 0.005874±0.017621 | 0 | 0 | 0 | 0 | 0 | 0 | 0 |
| *Lactobacillus_reuteri* | 0 | 0.000847±0.002810 | 0.000922±0.003058 | 0 | 0 | 0 | 0 | 0 |
| *Lactobacillus_rogosae* | 0 | 0.001001±0.003318 | 0.002247±0.007454 | 0 | 0.001126±0.003377 | 0 | 0.006386±0.014279 | 0.003409±0.006818 |
| *Lactobacillus_ruminis* | 0.001958±0.005874 | 0 | 0 | 0 | 0 | 0 | 0 | 0 |
| *Lactobacillus_sunkii* | 0 | 0 | 0 | 0 | 0 | 0 | 0.001356±0.003032 | 0 |
| *Lactobacillus_vaginalis* | 0.105727±0.317181 | 0.014290±0.023574 | 0.017253±0.027861 | 0.095110±0.303835 | 0.084878±0.250857 | 0.070893±0.183264 | 0.375467±0.825349 | 0.098830±0.090817 |
| *Lactobacillus_versmoldensis* | 0 | 0 | 0.000922±0.003058 | 0 | 0.001003±0.003010 | 0 | 0 | 0.003015±0.006031 |
| *Lactococcus_chungangensis* | 0.081825±0.022854 | 0.057696±0.039557 | 0.075749±0.063410 | 0.057183±0.037384 | 0.079442±0.053953 | 0.081140±0.062267 | 0.090256±0.092192 | 0.090219±0.034525 |
| *Lactococcus_garvieae* | 0 | 0.000393±0.001304 | 0.000631±0.002094 | 0 | 0.002888±0.008663 | 0 | 0.006386±0.014279 | 0 |
| *Lactococcus_lactis* | 0.745432±0.492017 | 0.940767±0.524074 | 0.976514±0.502562 | 0.703167±0.411135 | 0.749686±0.352579 | 0.748053±0.468616 | 0.671797±0.399203 | 0.936101±0.403140 |
| *Lactococcus_piscium* | 2.245191±1.014386 | 2.508988±1.322771 | 2.821999±1.379739 | 2.378212±1.324037 | 2.290751±0.844158 | 2.090058±1.014925 | 1.994249±0.959642 | 2.502100±0.668699 |
| *Lactococcus_plantarum* | 0.031985±0.024616 | 0.029323±0.037680 | 0.030109±0.029856 | 0.022131±0.029138 | 0.027249±0.024705 | 0.040632±0.025970 | 0.042300±0.037594 | 0.040523±0.022866 |
| *Lactococcus_raffinolactis* | 0.187481±0.117238 | 0.239949±0.123220 | 0.270096±0.107030 | 0.203396±0.107851 | 0.279037±0.121709 | 0.216178±0.169718 | 0.160769±0.105939 | 0.303280±0.139184 |
| *Leclercia_adecarboxylata* | 0 | 0.002055±0.006817 | 0 | 0 | 0 | 0 | 0.006386±0.014279 | 0 |
| *Legionella_beliardensis* | 0 | 0 | 0 | 0.003023±0.010027 | 0 | 0 | 0 | 0 |
| *Lelliottia_amnigena* | 0 | 0 | 0 | 0 | 0 | 0.008230±0.024691 | 0 | 0 |
| *Leucobacter_aridicollis* | 0 | 0 | 0 | 0 | 0 | 0 | 0 | 0.003409±0.006818 |
| *Leuconostoc_carnosum* | 0 | 0 | 0.000922±0.003058 | 0.000671±0.002225 | 0 | 0 | 0 | 0 |
| *Leuconostoc_citreum* | 0.008905±0.012467 | 0.008862±0.020004 | 0.001388±0.003103 | 0.002765±0.009170 | 0.001003±0.003010 | 0.005080±0.010121 | 0.006802±0.011792 | 0 |
| *Leuconostoc_fallax* | 0.002158±0.006475 | 0 | 0 | 0.003023±0.010027 | 0 | 0 | 0 | 0 |
| *Leuconostoc_gasicomitatum* | 0.020644±0.026494 | 0.016959±0.025344 | 0.019493±0.017898 | 0.009256±0.011217 | 0.015465±0.021940 | 0.012510±0.021842 | 0.023380±0.013650 | 0.026481±0.046241 |
| *Leuconostoc_lactis* | 0.009638±0.025328 | 0.016415±0.020626 | 0.014958±0.015241 | 0.028512±0.039979 | 0.048825±0.120499 | 0.012155±0.024504 | 0.016026±0.016498 | 0.009044±0.006165 |
| *Leuconostoc_mesenteroides* | 0.048662±0.045774 | 0.050213±0.041573 | 0.061481±0.065999 | 0.043024±0.036745 | 0.051660±0.036851 | 0.023964±0.027014 | 0.037779±0.067153 | 0.035529±0.041954 |
| *Leuconostoc_pseudomesenteroides* | 0.003860±0.007728 | 0 | 0.003566±0.011829 | 0.001607±0.005329 | 0.001003±0.003010 | 0.003250±0.009749 | 0 | 0 |
| *Limnobacter_litoralis* | 0.001695±0.005084 | 0.008910±0.017038 | 0 | 0 | 0 | 0 | 0 | 0 |
| *Limnobacter_thiooxidans* | 0.717787±0.980634 | 2.707934±2.562951 | 0.002270±0.007528 | 0.001607±0.005329 | 0 | 0 | 0.002711±0.006063 | 0 |
| *Limnohabitans_planktonicus* | 0 | 0 | 0.000757±0.002509 | 0 | 0 | 0 | 0 | 0 |
| *Lotus_japonicus* | 0 | 0 | 0.002766±0.009173 | 0 | 0 | 0 | 0 | 0 |
| *Luteimonas_terricola* | 0 | 0 | 0 | 0 | 0 | 0 | 0.006386±0.014279 | 0 |
| *Luteococcus_peritonei* | 0 | 0.001001±0.003318 | 0 | 0 | 0 | 0 | 0 | 0 |
| *Lysinibacillus_boronitolerans* | 0.186858±0.108872 | 0.296271±0.193092 | 0.238517±0.123139 | 0.177848±0.162284 | 0.197674±0.112780 | 0.190561±0.130431 | 0.165698±0.137527 | 0.279041±0.107108 |
| *Lysinibacillus_fusiformis* | 0.505539±0.158264 | 0.525637±0.220534 | 0.616937±0.171640 | 0.499542±0.134595 | 0.517062±0.120620 | 0.485461±0.200170 | 0.530431±0.213007 | 0.634666±0.111176 |
| *Lysinibacillus_meyeri* | 0.008076±0.014014 | 0.010365±0.022553 | 0.011111±0.018948 | 0.006394±0.012985 | 0.006514±0.012992 | 0.003239±0.009718 | 0 | 0.011268±0.014903 |
| *Lysinibacillus_sphaericus* | 0.035496±0.026731 | 0.040180±0.040368 | 0.037601±0.030673 | 0.059080±0.029543 | 0.032628±0.023203 | 0.064717±0.035929 | 0.058293±0.037669 | 0.045610±0.042125 |
| *Lysobacter_brunescens* | 0.024937±0.040462 | 0.046949±0.052504 | 0 | 0 | 0 | 0 | 0 | 0 |
| *Lysobacter_ginsengisoli* | 0 | 0 | 0 | 0 | 0 | 0.002336±0.007009 | 0 | 0 |
| *Lysobacter_pocheonensis* | 0 | 0 | 0.000922±0.003058 | 0 | 0.002158±0.006474 | 0 | 0 | 0 |
| *Macellibacteroides_fermentans* | 0.006807±0.020422 | 0 | 0 | 0 | 0 | 0 | 0 | 0 |
| *Macrococcus_brunensis* | 0 | 0 | 0 | 0.001531±0.005079 | 0 | 0 | 0 | 0 |
| *Macrococcus_caseolyticus* | 0.001129±0.003386 | 0.000847±0.002810 | 0.000757±0.002509 | 0.001342±0.004450 | 0.004503±0.013508 | 0 | 0.005096±0.007002 | 0.011270±0.013078 |
| *Magnetospirillum_magnetotacticum* | 0.018226±0.026718 | 0.107765±0.110804 | 0 | 0.002765±0.009170 | 0 | 0 | 0 | 0.003409±0.006818 |
| *Marinobacter_adhaerens* | 0.001958±0.005874 | 0 | 0 | 0 | 0 | 0 | 0 | 0 |
| *Marinobacter_algicola* | 0 | 0 | 0 | 0 | 0 | 0 | 0 | 0.005239±0.010479 |
| *Marinobacter_bryozoorum* | 0 | 0 | 0 | 0 | 0 | 0 | 0 | 0.003015±0.006031 |
| *Marixanthomonas_ophiurae* | 0 | 0 | 0.001093±0.003625 | 0 | 0 | 0 | 0 | 0.003409±0.006818 |
| *Massilia_aurea* | 0.008730±0.020431 | 0.005387±0.009939 | 0.002651±0.006527 | 0.005706±0.015315 | 0.006204±0.018612 | 0 | 0.004067±0.009095 | 0 |
| *Massilia_niabensis* | 0 | 0 | 0 | 0 | 0.003392±0.010175 | 0 | 0 | 0 |
| *Massilia_plicata* | 0 | 0 | 0 | 0 | 0 | 0 | 0 | 0.002620±0.005239 |
| *Massilia_tieshanensis* | 0.005661±0.011526 | 0.002001±0.006637 | 0.000631±0.002094 | 0.003407±0.007627 | 0 | 0 | 0 | 0 |
| *Massilia_timonae* | 0.037709±0.045233 | 0.047008±0.048496 | 0.004012±0.008400 | 0.003355±0.011126 | 0 | 0 | 0.007741±0.013836 | 0.005635±0.006539 |
| *Massilia_varians* | 0.001695±0.005084 | 0.005219±0.010764 | 0 | 0.000671±0.002225 | 0 | 0 | 0 | 0 |
| *Massilia_yuzhufengensis* | 0.009161±0.017748 | 0.010728±0.025703 | 0 | 0.001342±0.004450 | 0 | 0 | 0.001356±0.003032 | 0 |
| *Megamonas_rupellensis* | 0 | 0 | 0 | 0 | 0 | 0 | 0.019157±0.042837 | 0 |
| *Methylobacterium_adhaesivum* | 0.001702±0.005105 | 0 | 0 | 0 | 0 | 0 | 0 | 0 |
| *Methylobacterium_aminovorans* | 0.001695±0.005084 | 0.002739±0.006614 | 0 | 0 | 0 | 0 | 0 | 0 |
| *Methylobacterium_aquaticum* | 0 | 0 | 0 | 0 | 0 | 0 | 0 | 0.003409±0.006818 |
| *Methylobacterium_dankookense* | 0.009296±0.017122 | 0.009344±0.017803 | 0 | 0 | 0 | 0 | 0 | 0 |
| *Methylobacterium_goesingense* | 0 | 0 | 0.002186±0.007250 | 0 | 0 | 0 | 0.001356±0.003032 | 0 |
| *Methylobacterium_gregans* | 0 | 0.002494±0.008272 | 0 | 0 | 0 | 0 | 0 | 0 |
| *Methylobacterium_hispanicum* | 0 | 0 | 0.002589±0.005762 | 0 | 0.005935±0.017806 | 0 | 0.001356±0.003032 | 0 |
| *Methylobacterium_jeotgali* | 0.005045±0.011812 | 0 | 0 | 0 | 0 | 0.003239±0.009718 | 0.009097±0.014048 | 0 |
| *Methylobacterium_marchantiae* | 0.001129±0.003386 | 0.001193±0.003957 | 0 | 0 | 0 | 0 | 0 | 0 |
| *Methylobacterium_zatmanii* | 0.001695±0.005084 | 0.013894±0.029317 | 0 | 0 | 0 | 0 | 0 | 0 |
| *Methylophilus_leisingeri* | 0 | 0.006025±0.019984 | 0 | 0 | 0 | 0 | 0 | 0 |
| *Methyloversatilis_universalis* | 0.001885±0.005655 | 0.001339±0.004441 | 0.005531±0.018346 | 0 | 0.001126±0.003377 | 0 | 0 | 0 |
| *Microbacterium_chocolatum* | 0 | 0 | 0 | 0 | 0 | 0 | 0.002385±0.005332 | 0.003409±0.006818 |
| *Microbacterium_testaceum* | 0.001885±0.005655 | 0 | 0 | 0 | 0 | 0 | 0 | 0 |
| *Microbacterium_trichothecenolyticum* | 0 | 0 | 0 | 0.000671±0.002225 | 0 | 0 | 0 | 0 |
| *Micrococcus_antarcticus* | 0 | 0 | 0 | 0 | 0 | 0 | 0.001356±0.003032 | 0 |
| *Micrococcus_endophyticus* | 0 | 0 | 0 | 0.000671±0.002225 | 0 | 0 | 0 | 0 |
| *Micrococcus_flavus* | 0 | 0 | 0 | 0 | 0 | 0 | 0 | 0.002620±0.005239 |
| *Micrococcus_terreus* | 0 | 0 | 0 | 0 | 0.003546±0.010639 | 0 | 0.002385±0.005332 | 0 |
| *Microvirgula_aerodenitrificans* | 0.001666±0.004999 | 0 | 0 | 0 | 0.003546±0.010639 | 0 | 0 | 0.015077±0.030153 |
| *Mitsuaria_chitosanitabida* | 0 | 0 | 0.003979±0.013195 | 0.004886±0.011759 | 0 | 0 | 0 | 0 |
| *Mobilicoccus_pelagius* | 0 | 0 | 0 | 0.002910±0.009651 | 0 | 0 | 0 | 0 |
| *Moheibacter_sediminis* | 0.001958±0.005874 | 0 | 0 | 0 | 0 | 0 | 0 | 0 |
| *Moranella_endobia* | 0 | 0.011675±0.036348 | 0 | 0 | 0 | 0 | 0 | 0 |
| *Mucispirillum_schaedleri* | 0 | 0 | 0.001326±0.004398 | 0 | 0 | 0 | 0 | 0 |
| *Mycobacterium_monacense* | 0 | 0 | 0 | 0 | 0.003102±0.009306 | 0 | 0 | 0 |
| *Myroides_odoratimimus* | 0.031007±0.036387 | 0.027949±0.031244 | 0.049738±0.053600 | 0.031940±0.027213 | 0.052689±0.053680 | 0.029735±0.041108 | 0.067170±0.057164 | 0.034597±0.027304 |
| *Myroides_profundi* | 0.010889±0.020420 | 0.012819±0.020617 | 0.018357±0.016579 | 0.009177±0.014375 | 0.015931±0.019523 | 0.009114±0.013958 | 0.006779±0.015158 | 0.005635±0.006539 |
| *Naxibacter_varians* | 0.035397±0.032213 | 0.043719±0.036621 | 0.001263±0.004188 | 0.005076±0.011584 | 0.003546±0.010639 | 0 | 0 | 0 |
| *Noviherbaspirillum_malthae* | 0 | 0 | 0 | 0 | 0.001126±0.003377 | 0 | 0 | 0 |
| *Noviherbaspirillum_soli* | 0 | 0 | 0 | 0.001531±0.005079 | 0 | 0 | 0 | 0 |
| *Noviherbaspirillum_suwonense* | 0 | 0 | 0 | 0.001876±0.006221 | 0 | 0 | 0 | 0 |
| *Novosphingobium_taihuense* | 0 | 0.001193±0.003957 | 0 | 0 | 0 | 0 | 0 | 0 |
| *Nubsella_zeaxanthinifaciens* | 0 | 0 | 0 | 0.002684±0.008901 | 0 | 0 | 0 | 0 |
| *Obesumbacterium_proteus* | 0 | 0 | 0 | 0 | 0 | 0.002743±0.008230 | 0 | 0 |
| *Oceanobacillus_chironomi* | 0.003825±0.007670 | 0 | 0.004441±0.008212 | 0.003751±0.012441 | 0.003546±0.010639 | 0 | 0 | 0 |
| *Oceanobacillus_profundus* | 2.339611±1.200381 | 2.572415±1.158759 | 2.812652±0.773033 | 2.137892±0.419737 | 2.743066±1.383793 | 2.409274±0.937884 | 3.504458±1.552259 | 3.437557±0.749671 |
| *Ochrobactrum_lupini* | 0.001702±0.005105 | 0.000669±0.002220 | 0.001093±0.003625 | 0.001607±0.005329 | 0 | 0 | 0.002385±0.005332 | 0.006818±0.013635 |
| *Ochrobactrum_pseudogrignonense* | 0 | 0 | 0 | 0.000671±0.002225 | 0 | 0 | 0 | 0 |
| *Odoribacter_splanchnicus* | 0 | 0 | 0.000631±0.002094 | 0.000671±0.002225 | 0 | 0 | 0 | 0 |
| *Oribacterium_sinus* | 0.001129±0.003386 | 0 | 0 | 0 | 0 | 0 | 0 | 0 |
| *Ornithinibacillus_californiensis* | 0 | 0.000847±0.002810 | 0 | 0 | 0 | 0 | 0 | 0 |
| *Oscillibacter_valericigenes* | 0.010498±0.008265 | 0.024328±0.038635 | 0.031197±0.033381 | 0.032408±0.038676 | 0.082043±0.201949 | 0.029216±0.031688 | 0.023291±0.028115 | 0.021890±0.023400 |
| *Paenalcaligenes_hominis* | 0 | 0 | 0.000631±0.002094 | 0 | 0.005126±0.010333 | 0 | 0 | 0 |
| *Paenibacillus_alvei* | 0.001958±0.005874 | 0 | 0 | 0 | 0 | 0 | 0 | 0 |
| *Paenibacillus_amylolyticus* | 0.004532±0.010348 | 0.015765±0.024848 | 0.016156±0.015219 | 0.008657±0.014856 | 0.005219±0.010481 | 0.011544±0.017694 | 0.002711±0.006063 | 0.009439±0.011710 |
| *Paenibacillus_barcinonensis* | 0.003404±0.010211 | 0.005365±0.010840 | 0.011455±0.010902 | 0.008450±0.012661 | 0.004550±0.010689 | 0.012762±0.029442 | 0.006602±0.006612 | 0.025299±0.029847 |
| *Paenibacillus_edaphicus* | 0 | 0 | 0 | 0 | 0 | 0 | 0 | 0.002620±0.005239 |
| *Paenibacillus_humicus* | 0 | 0 | 0 | 0 | 0.001003±0.003010 | 0 | 0 | 0 |
| *Paenibacillus_illinoisensis* | 0 | 0 | 0.001357±0.004502 | 0.003436±0.009217 | 0 | 0 | 0 | 0 |
| *Paenibacillus_lactis* | 0 | 0.001670±0.003804 | 0 | 0.003063±0.010159 | 0.001510±0.004529 | 0 | 0.004769±0.010664 | 0 |
| *Paenibacillus_larvae* | 0.008778±0.011779 | 0.033065±0.037118 | 0.004528±0.007068 | 0.019213±0.021593 | 0.005126±0.010333 | 0.003250±0.009749 | 0.002385±0.005332 | 0.005635±0.006539 |
| *Paenibacillus_lautus* | 0 | 0.001339±0.004441 | 0 | 0 | 0.003377±0.010131 | 0.003250±0.009749 | 0 | 0 |
| *Paenibacillus_nanensis* | 0 | 0 | 0 | 0.000671±0.002225 | 0 | 0 | 0 | 0 |
| *Paenibacillus_pasadenensis* | 0 | 0 | 0.001263±0.004188 | 0 | 0 | 0 | 0 | 0.005239±0.010479 |
| *Paenibacillus_validus* | 0.010826±0.016738 | 0.006988±0.008873 | 0.028946±0.059521 | 0.018935±0.023411 | 0.016176±0.044854 | 0.006355±0.019064 | 0.062238±0.073746 | 0.110229±0.080105 |
| *Paenibacillus_xylanexedens* | 0 | 0 | 0.002937±0.006790 | 0.000671±0.002225 | 0.002129±0.004234 | 0.003239±0.009718 | 0.001356±0.003032 | 0 |
| *Paludibacter_propionicigenes* | 0.001958±0.005874 | 0.001694±0.005619 | 0.002270±0.007528 | 0.003751±0.012441 | 0 | 0.009412±0.016911 | 0.002711±0.006063 | 0.003015±0.006031 |
| *Pantoea_agglomerans* | 0.001129±0.003386 | 0.007004±0.023229 | 0.001958±0.004679 | 0.001342±0.004450 | 0.021714±0.065141 | 0 | 0.495418±1.072447 | 0.006818±0.013635 |
| *Pantoea_eucrina* | 0.001129±0.003386 | 0 | 0.003279±0.010876 | 0 | 0 | 0 | 0 | 0 |
| *Parabacteroides_merdae* | 0 | 0 | 0 | 0 | 0.001126±0.003377 | 0.003239±0.009718 | 0 | 0 |
| *Paracoccus_aminovorans* | 0 | 0 | 0 | 0.003023±0.010027 | 0 | 0 | 0 | 0 |
| *Paracoccus_chinensis* | 0 | 0 | 0.001679±0.003757 | 0.001531±0.005079 | 0 | 0 | 0.006386±0.014279 | 0.004077±0.008154 |
| *Paracoccus_marcusii* | 0.004260±0.008707 | 0.001456±0.003252 | 0.022314±0.019059 | 0.029258±0.051319 | 0.008622±0.015035 | 0.020871±0.021816 | 0.017346±0.026386 | 0.026613±0.026644 |
| *Paracoccus_marinus* | 0.017822±0.046625 | 0.000669±0.002220 | 0 | 0.001876±0.006221 | 0 | 0.003378±0.010135 | 0 | 0 |
| *Paracoccus_sphaerophysae* | 0 | 0 | 0 | 0 | 0 | 0 | 0.001356±0.003032 | 0 |
| *Paracoccus_tibetensis* | 0.001129±0.003386 | 0.000847±0.002810 | 0.008813±0.019206 | 0.003407±0.007627 | 0 | 0 | 0.007831±0.012012 | 0 |
| *Paracoccus_yeei* | 0 | 0.000393±0.001304 | 0 | 0 | 0 | 0 | 0 | 0 |
| *Parapusillimonas_granuli* | 0 | 0 | 0 | 0.002553±0.008467 | 0 | 0 | 0 | 0 |
| *Parasutterella_excrementihominis* | 0.001958±0.005874 | 0 | 0 | 0 | 0 | 0 | 0 | 0 |
| *Parasutterella_secunda* | 0.002158±0.006475 | 0.003389±0.011239 | 0.001093±0.003625 | 0.004846±0.010397 | 0.012408±0.037223 | 0 | 0 | 0 |
| *Pectinatus_portalensis* | 0 | 0.001694±0.005619 | 0 | 0 | 0 | 0 | 0 | 0 |
| *Pediococcus_acidilactici* | 0 | 0 | 0 | 0 | 0 | 0.003239±0.009718 | 0 | 0.002620±0.005239 |
| *Pediococcus_cellicola* | 0 | 0.002055±0.006817 | 0 | 0 | 0 | 0 | 0 | 0 |
| *Pediococcus_ethanolidurans* | 0.003916±0.011747 | 0.007560±0.020277 | 0.002279±0.005183 | 0.003213±0.010658 | 0.007604±0.015415 | 0.003239±0.009718 | 0.009490±0.021221 | 0.012725±0.009007 |
| *Pedobacter_bauzanensis* | 0 | 0.009704±0.020331 | 0.008657±0.014892 | 0.005106±0.016934 | 0.002968±0.008903 | 0 | 0 | 0.015470±0.022776 |
| *Pedobacter_duraquae* | 0.003087±0.006403 | 0.000393±0.001304 | 0 | 0.000671±0.002225 | 0 | 0 | 0 | 0 |
| *Pedobacter_steynii* | 0 | 0 | 0 | 0 | 0.004228±0.009494 | 0 | 0 | 0 |
| *Pelobacter_propionicus* | 0.001702±0.005105 | 0 | 0 | 0 | 0 | 0.015205±0.031638 | 0 | 0 |
| *Pelomonas_puraquae* | 0 | 0 | 0.001326±0.004398 | 0.000671±0.002225 | 0 | 0 | 0 | 0 |
| *Pelomonas_saccharophila* | 0.003087±0.006403 | 0.006326±0.018298 | 0 | 0 | 0 | 0 | 0 | 0 |
| *Peptoniphilus_gorbachii* | 0 | 0 | 0 | 0 | 0 | 0 | 0.002385±0.005332 | 0.003409±0.006818 |
| *Peptoniphilus_indolicus* | 0 | 0 | 0 | 0 | 0 | 0 | 0 | 0.003015±0.006031 |
| *Peptoniphilus_ivorii* | 0 | 0 | 0 | 0.001531±0.005079 | 0 | 0 | 0 | 0 |
| *Peptostreptococcus_anaerobius* | 0.001666±0.004999 | 0 | 0 | 0 | 0 | 0 | 0 | 0 |
| *Perlucidibaca_piscinae* | 0.001702±0.005105 | 0 | 0 | 0 | 0 | 0 | 0 | 0 |
| *Petrimonas_sulfuriphila* | 0 | 0.001193±0.003957 | 0 | 0 | 0 | 0 | 0.001356±0.003032 | 0 |
| *Phascolarctobacterium_faecium* | 0.007157±0.015574 | 0.019555±0.022148 | 0.039188±0.036308 | 0.034721±0.047671 | 0.047484±0.092539 | 0.016023±0.025646 | 0.020930±0.026519 | 0.012332±0.008988 |
| *Phenylobacterium_haematophilum* | 0 | 0 | 0 | 0 | 0 | 0 | 0 | 0.003409±0.006818 |
| *Phycisphaera_mikurensis* | 0 | 0.001001±0.003318 | 0 | 0 | 0 | 0 | 0 | 0 |
| *Pinus_koraiensis* | 0 | 0.001001±0.003318 | 0.001263±0.004188 | 0 | 0.001126±0.003377 | 0 | 0 | 0 |
| *Pirellula_staleyi* | 0 | 0.002001±0.006637 | 0 | 0 | 0 | 0 | 0 | 0 |
| *Planobacterium_taklimakanense* | 0 | 0 | 0 | 0 | 0 | 0 | 0.002385±0.005332 | 0 |
| *Planococcus_antarcticus* | 0.001702±0.005105 | 0 | 0.013920±0.034049 | 0.004078±0.007622 | 0.017732±0.053197 | 0.003044±0.009132 | 0 | 0.006818±0.013635 |
| *Planococcus_maitriensis* | 0 | 0.002055±0.006817 | 0 | 0.001607±0.005329 | 0 | 0 | 0 | 0 |
| *Planococcus_pelagicus* | 0 | 0 | 0 | 0 | 0 | 0 | 0 | 0.004077±0.008154 |
| *Planococcus_rifietoensis* | 0.001666±0.004999 | 0 | 0 | 0 | 0 | 0 | 0 | 0 |
| *Planococcus_salinarum* | 0 | 0 | 0 | 0.000671±0.002225 | 0 | 0 | 0 | 0 |
| *Planomicrobium_chinense* | 0 | 0.000847±0.002810 | 0 | 0 | 0 | 0 | 0 | 0 |
| *Planomicrobium_flavidum* | 0 | 0 | 0.001513±0.005019 | 0.001863±0.006180 | 0 | 0.002336±0.007009 | 0 | 0.004077±0.008154 |
| *Planomicrobium_koreense* | 0 | 0 | 0 | 0 | 0 | 0 | 0 | 0.002620±0.005239 |
| *Planomicrobium_okeanokoites* | 0 | 0.000847±0.002810 | 0 | 0 | 0 | 0 | 0 | 0.003409±0.006818 |
| *Polaromonas_aquatica* | 0 | 0 | 0 | 0 | 0.001003±0.003010 | 0 | 0 | 0 |
| *Polaromonas_naphthalenivorans* | 0 | 0 | 0 | 0.000671±0.002225 | 0.001003±0.003010 | 0 | 0 | 0.002620±0.005239 |
| *Pontibacter_akesuensis* | 0.001129±0.003386 | 0.001001±0.003318 | 0 | 0 | 0 | 0 | 0 | 0 |
| *Porphyrobacter_donghaensis* | 0.002594±0.007781 | 0 | 0.002450±0.005473 | 0.002553±0.008467 | 0 | 0.004828±0.010275 | 0.001356±0.003032 | 0 |
| *Porphyromonas_catoniae* | 0 | 0 | 0.000922±0.003058 | 0 | 0 | 0 | 0 | 0 |
| *Prevotella_copri* | 0.001958±0.005874 | 0.006067±0.013610 | 0.023069±0.056655 | 0 | 0.001003±0.003010 | 0 | 0.011809±0.016259 | 0.009437±0.012874 |
| *Prevotella_heparinolytica* | 0 | 0 | 0.001844±0.006115 | 0 | 0 | 0 | 0 | 0 |
| *Prevotella_melaninogenica* | 0 | 0 | 0 | 0.001531±0.005079 | 0 | 0 | 0 | 0 |
| *Prevotella_stercorea* | 0 | 0 | 0.001093±0.003625 | 0 | 0 | 0 | 0 | 0 |
| *Propionibacterium_acnes* | 0.026109±0.037999 | 0.008956±0.016832 | 0.029073±0.027870 | 0.034902±0.057761 | 0.061082±0.127601 | 0.023087±0.025185 | 0.033350±0.053914 | 0.025553±0.023299 |
| *Proteiniclasticum_ruminis* | 0.008937±0.020555 | 0 | 0.007945±0.018277 | 0.006304±0.014332 | 0.004672±0.010753 | 0.003044±0.009132 | 0 | 0.004077±0.008154 |
| *Proteus_hauseri* | 0 | 0 | 0 | 0 | 0 | 0.003239±0.009718 | 0 | 0 |
| *Proteus_vulgaris* | 0 | 0 | 0 | 0 | 0 | 0.002336±0.007009 | 0 | 0 |
| *Providencia_heimbachae* | 0 | 0 | 0 | 0 | 0.002158±0.006474 | 0 | 0 | 0 |
| *Pseudochrobactrum_kiredjianiae* | 0 | 0 | 0 | 0.002013±0.006676 | 0.001126±0.003377 | 0 | 0 | 0.003409±0.006818 |
| *Pseudoflavonifractor_capillosus* | 0 | 0.002893±0.006799 | 0.002015±0.004503 | 0 | 0.014981±0.027833 | 0.003239±0.009718 | 0 | 0.003015±0.006031 |
| *Pseudomonas_alcaligenes* | 0.797843±1.118260 | 2.672193±2.754731 | 0.003164±0.007212 | 0.003544±0.007974 | 0 | 0 | 0.001356±0.003032 | 0.029890±0.053025 |
| *Pseudomonas_alcaliphila* | 0.035759±0.035101 | 0.011185±0.015425 | 0.032250±0.033021 | 0.053865±0.048989 | 0.040354±0.029575 | 0.012872±0.022166 | 0.007154±0.015997 | 0.009316±0.010924 |
| *Pseudomonas_azotoformans* | 0.003587±0.007130 | 0.001193±0.003957 | 0.000631±0.002094 | 0 | 0 | 0 | 0 | 0 |
| *Pseudomonas_balearica* | 0 | 0 | 0 | 0.000671±0.002225 | 0 | 0 | 0 | 0 |
| *Pseudomonas_bauzanensis* | 0 | 0.001193±0.003957 | 0.006294±0.012772 | 0.011278±0.018411 | 0.007335±0.014845 | 0.003498±0.010495 | 0.003740±0.005435 | 0.023226±0.008838 |
| *Pseudomonas_beteli* | 0.035943±0.039658 | 0.077989±0.064257 | 0.104584±0.122960 | 0.116048±0.163416 | 0.024506±0.034446 | 0.046001±0.038586 | 0.236371±0.298642 | 0.532527±0.398911 |
| *Pseudomonas_brenneri* | 0 | 0 | 0 | 0.004628±0.010533 | 0.005319±0.012921 | 0.006583±0.013109 | 0.001356±0.003032 | 0.004077±0.008154 |
| *Pseudomonas_caeni* | 0 | 0.002634±0.004544 | 0.010431±0.011653 | 0 | 0.008409±0.014527 | 0.013487±0.026790 | 0.005423±0.012126 | 0.002620±0.005239 |
| *Pseudomonas_cedrina* | 0.016272±0.015258 | 0.026056±0.019132 | 0.029207±0.031069 | 0.010932±0.021298 | 0.048043±0.041737 | 0.035909±0.026849 | 0.029565±0.024187 | 0.024905±0.022470 |
| *Pseudomonas_chlororaphis* | 0.024513±0.020623 | 0.006305±0.009695 | 0.012687±0.012982 | 0.025893±0.020549 | 0.010799±0.013583 | 0.011750±0.023934 | 0.010968±0.018592 | 0.009712±0.006927 |
| *Pseudomonas_deceptionensis* | 0.001129±0.003386 | 0 | 0.001679±0.003757 | 0 | 0.003546±0.010639 | 0 | 0.002861±0.006398 | 0.008255±0.010200 |
| *Pseudomonas_duriflava* | 0 | 0.004236±0.014049 | 0 | 0 | 0.001003±0.003010 | 0 | 0 | 0 |
| *Pseudomonas_extremaustralis* | 0.001666±0.004999 | 0.001193±0.003957 | 0 | 0 | 0 | 0.003250±0.009749 | 0 | 0.004077±0.008154 |
| *Pseudomonas_fragi* | 1.717911±1.072960 | 1.533698±0.822691 | 1.893200±1.071306 | 1.631083±0.954538 | 1.826428±0.995458 | 1.405177±0.861193 | 1.480601±0.950355 | 1.856058±0.814231 |
| *Pseudomonas_fulva* | 0.001129±0.003386 | 0.001848±0.004128 | 0 | 0.001531±0.005079 | 0.001510±0.004529 | 0.005983±0.011924 | 0.019157±0.042837 | 0 |
| *Pseudomonas_gessardii* | 0.016246±0.018713 | 0.011062±0.012347 | 0.004464±0.007797 | 0.014836±0.020641 | 0.022902±0.028882 | 0.018378±0.031010 | 0.002385±0.005332 | 0.005239±0.010479 |
| *Pseudomonas_graminis* | 0 | 0 | 0 | 0 | 0 | 0 | 0.002385±0.005332 | 0 |
| *Pseudomonas_hibiscicola* | 0 | 0.001193±0.003957 | 0 | 0.003023±0.010027 | 0 | 0.003250±0.009749 | 0 | 0.002620±0.005239 |
| *Pseudomonas_lurida* | 0.017634±0.016723 | 0.005527±0.009904 | 0.020629±0.019151 | 0.014273±0.022327 | 0.010809±0.016463 | 0.012895±0.029519 | 0.008837±0.012163 | 0.035386±0.024064 |
| *Pseudomonas_luteola* | 0 | 0.000847±0.002810 | 0 | 0 | 0 | 0 | 0 | 0 |
| *Pseudomonas_oleovorans* | 0 | 0.003478±0.007007 | 0.002532±0.006442 | 0.017981±0.029111 | 0 | 0 | 0 | 0 |
| *Pseudomonas_oryzihabitans* | 0 | 0.001517±0.003403 | 0.006253±0.014509 | 0 | 0.017082±0.031921 | 0.021375±0.022510 | 0.002385±0.005332 | 0 |
| *Pseudomonas_pelagia* | 0.002158±0.006475 | 0 | 0 | 0 | 0 | 0 | 0 | 0 |
| *Pseudomonas_peli* | 0 | 0 | 0.002459±0.008156 | 0 | 0.003546±0.010639 | 0 | 0 | 0 |
| *Pseudomonas_pertucinogena* | 0.001695±0.005084 | 0.000393±0.001304 | 0 | 0 | 0.007862±0.015697 | 0.003378±0.010135 | 0 | 0.003015±0.006031 |
| *Pseudomonas_plecoglossicida* | 0.003287±0.006922 | 0.004369±0.008760 | 0.003785±0.008870 | 0.006653±0.011636 | 0 | 0 | 0.015483±0.027672 | 0 |
| *Pseudomonas_proteolytica* | 0.008385±0.011284 | 0.005128±0.008638 | 0.006619±0.009558 | 0.015730±0.025157 | 0.002007±0.006020 | 0.006417±0.012734 | 0.006125±0.010332 | 0.005239±0.010479 |
| *Pseudomonas_psychrophila* | 0.008834±0.015168 | 0.006081±0.010106 | 0.000757±0.002509 | 0.002987±0.006907 | 0.014346±0.015408 | 0.008586±0.020936 | 0 | 0 |
| *Pseudomonas_putida* | 0 | 0 | 0 | 0 | 0.001510±0.004529 | 0.001589±0.004766 | 0 | 0 |
| *Pseudomonas_rhizosphaerae* | 0 | 0 | 0 | 0.001531±0.005079 | 0 | 0 | 0 | 0.002620±0.005239 |
| *Pseudomonas_stutzeri* | 0.012052±0.020116 | 0.011298±0.018818 | 0.080942±0.066356 | 0.086894±0.125410 | 0.025905±0.018353 | 0.016202±0.012989 | 0.047373±0.036023 | 0.048384±0.023874 |
| *Pseudomonas_syringae* | 0 | 0.002055±0.006817 | 0.002310±0.004021 | 0.004547±0.009495 | 0.002635±0.005300 | 0 | 0 | 0 |
| *Pseudomonas_umsongensis* | 0.001885±0.005655 | 0 | 0.001989±0.004771 | 0.006023±0.010593 | 0 | 0 | 0 | 0.002620±0.005239 |
| *Pseudomonas_vancouverensis* | 0 | 0.000393±0.001304 | 0 | 0 | 0 | 0 | 0 | 0 |
| *Pseudomonas_veronii* | 0.534332±0.412810 | 0.449599±0.258600 | 0.539768±0.302410 | 0.440871±0.287148 | 0.566138±0.242964 | 0.535303±0.359979 | 0.424194±0.338810 | 0.565414±0.297377 |
| *Pseudomonas_viridiflava* | 0.001129±0.003386 | 0 | 0 | 0.001531±0.005079 | 0.003019±0.009058 | 0 | 0 | 0 |
| *Pseudomonas_xanthomarina* | 0 | 0.000847±0.002810 | 0.006543±0.015096 | 0.003581±0.009685 | 0.003546±0.010639 | 0.008597±0.020970 | 0.006386±0.014279 | 0.005635±0.006539 |
| *Pseudomonas_xinjiangensis* | 0 | 0 | 0 | 0.000671±0.002225 | 0 | 0 | 0 | 0 |
| *Pseudoxanthomonas_mexicana* | 0 | 0 | 0.001263±0.004188 | 0.000671±0.002225 | 0 | 0 | 0 | 0 |
| *Psychrobacillus_psychrodurans* | 0.001129±0.003386 | 0 | 0 | 0 | 0 | 0 | 0 | 0 |
| *Psychrobacter_alimentarius* | 0.004116±0.008180 | 0.000847±0.002810 | 0.005347±0.013772 | 0.004519±0.007965 | 0.004672±0.010753 | 0.007376±0.011592 | 0 | 0 |
| *Psychrobacter_aquimaris* | 0 | 0 | 0 | 0 | 0.004477±0.009471 | 0 | 0 | 0 |
| *Psychrobacter_arcticus* | 0.001129±0.003386 | 0 | 0 | 0 | 0 | 0 | 0 | 0 |
| *Psychrobacter_arenosus* | 0.069069±0.066379 | 0.066069±0.075019 | 0.069843±0.049921 | 0.049077±0.046735 | 0.054833±0.038715 | 0.050524±0.050427 | 0.047620±0.060146 | 0.062538±0.038743 |
| *Psychrobacter_cibarius* | 0.545397±0.466404 | 0.444693±0.284199 | 0.611679±0.371268 | 0.616761±0.463593 | 0.562787±0.283500 | 0.500133±0.337408 | 0.443654±0.230908 | 0.715907±0.347623 |
| *Psychrobacter_cryohalolentis* | 0.005710±0.008636 | 0.006189±0.010785 | 0.007868±0.008898 | 0.005516±0.010807 | 0.010516±0.023274 | 0 | 0.006125±0.010332 | 0.003015±0.006031 |
| *Psychrobacter_faecalis* | 0.003660±0.007285 | 0.003770±0.009451 | 0.007963±0.009659 | 0.002553±0.008467 | 0.072716±0.087724 | 0.044388±0.056131 | 0.005447±0.012179 | 0.026119±0.023149 |
| *Psychrobacter_fozii* | 0.012828±0.016541 | 0.005865±0.013349 | 0.010461±0.012371 | 0.005218±0.009964 | 0.004258±0.008467 | 0.004332±0.008980 | 0 | 0.003409±0.006818 |
| *Psychrobacter_fulvigenes* | 0.001958±0.005874 | 0 | 0 | 0 | 0 | 0.007912±0.016027 | 0 | 0.005635±0.006539 |
| *Psychrobacter_luti* | 0 | 0 | 0 | 0 | 0.003392±0.010175 | 0 | 0 | 0 |
| *Psychrobacter_maritimus* | 0.020706±0.024456 | 0.019489±0.027715 | 0.039914±0.037601 | 0.020189±0.023130 | 0.022377±0.034078 | 0.068659±0.097275 | 0.008134±0.018189 | 0.028444±0.037609 |
| *Psychrobacter_namhaensis* | 0 | 0 | 0 | 0.002765±0.009170 | 0 | 0 | 0 | 0 |
| *Psychrobacter_sanguinis* | 0.011247±0.018767 | 0.000393±0.001304 | 0.002705±0.006381 | 0.001342±0.004450 | 0.003161±0.006790 | 0.007077±0.021231 | 0 | 0.009712±0.006927 |
| *Psychrobacter_urativorans* | 0.020884±0.025960 | 0.022282±0.024924 | 0.017808±0.019386 | 0.019685±0.022090 | 0.006893±0.011218 | 0.017718±0.019679 | 0.019706±0.020126 | 0.021101±0.018493 |
| *Rahnella_aquatilis* | 0.014609±0.024469 | 0.017853±0.021414 | 0.024788±0.027802 | 0.014976±0.021625 | 0.015018±0.020444 | 0.024399±0.035432 | 0.020058±0.028270 | 0.052039±0.039774 |
| *Ralstonia_mannitolilytica* | 0 | 0.000669±0.002220 | 0.013193±0.037527 | 0 | 0 | 0 | 0 | 0 |
| *Ralstonia_pickettii* | 0.001702±0.005105 | 0.012773±0.025116 | 0.115446±0.343154 | 0.011506±0.017663 | 0 | 0.003044±0.009132 | 0.002711±0.006063 | 0.006029±0.007079 |
| *Raoultella_terrigena* | 0 | 0 | 0 | 0.000671±0.002225 | 0.003546±0.010639 | 0.005487±0.016461 | 0.002711±0.006063 | 0 |
| *Rheinheimera_aquimaris* | 0 | 0 | 0 | 0.003023±0.010027 | 0 | 0 | 0 | 0 |
| *Rheinheimera_chironomi* | 0 | 0 | 0.000757±0.002509 | 0 | 0 | 0 | 0 | 0 |
| *Rheinheimera_longhuensis* | 0 | 0 | 0 | 0 | 0 | 0.002336±0.007009 | 0 | 0 |
| *Rheinheimera_perlucida* | 0 | 0 | 0 | 0 | 0 | 0 | 0 | 0.006029±0.007079 |
| *Rheinheimera_tangshanensis* | 0 | 0.000847±0.002810 | 0.000922±0.003058 | 0 | 0 | 0 | 0 | 0 |
| *Rheinheimera_texasensis* | 0 | 0 | 0.007289±0.013382 | 0.001342±0.004450 | 0 | 0 | 0 | 0 |
| *Rhizobacter_dauci* | 0.005610±0.012203 | 0.005775±0.016489 | 0 | 0 | 0 | 0 | 0 | 0 |
| *Rhizobium_borbori* | 0 | 0 | 0 | 0 | 0.005353±0.010793 | 0 | 0 | 0 |
| *Rhizobium_daejeonense* | 0 | 0.002449±0.006811 | 0 | 0 | 0 | 0 | 0 | 0 |
| *Rhizobium_giardinii* | 0.001129±0.003386 | 0.007565±0.025091 | 0 | 0 | 0 | 0 | 0 | 0 |
| *Rhizobium_larrymoorei* | 0 | 0.003556±0.009296 | 0 | 0 | 0 | 0 | 0 | 0 |
| *Rhizobium_radiobacter* | 0.001958±0.005874 | 0.001001±0.003318 | 0.004712±0.010516 | 0.003314±0.006058 | 0 | 0 | 0 | 0 |
| *Rhizobium_rosettiformans* | 0 | 0 | 0.001093±0.003625 | 0.000671±0.002225 | 0.001126±0.003377 | 0 | 0 | 0 |
| *Rhodobacter_blasticus* | 0 | 0 | 0.000922±0.003058 | 0 | 0 | 0 | 0 | 0 |
| *Rhodobacter_capsulatus* | 0.011491±0.029174 | 0.000847±0.002810 | 0.004729±0.010531 | 0.003751±0.012441 | 0.002513±0.005115 | 0 | 0.004067±0.009095 | 0 |
| *Rhodococcus_qingshengii* | 0 | 0 | 0.000922±0.003058 | 0.001531±0.005079 | 0 | 0 | 0 | 0 |
| *Rhodococcus_ruber* | 0 | 0 | 0.000757±0.002509 | 0 | 0 | 0 | 0 | 0 |
| *Rhodocytophaga_aerolata* | 0 | 0 | 0.003067±0.005698 | 0 | 0 | 0.003044±0.009132 | 0 | 0 |
| *Rhodopirellula_baltica* | 0 | 0 | 0 | 0 | 0 | 0 | 0 | 0.003409±0.006818 |
| *Roseburia_faecis* | 0 | 0 | 0.000631±0.002094 | 0 | 0.001510±0.004529 | 0.006479±0.019436 | 0.012771±0.028558 | 0 |
| *Roseburia_hominis* | 0 | 0 | 0.000757±0.002509 | 0 | 0 | 0 | 0 | 0 |
| *Roseburia_intestinalis* | 0.001129±0.003386 | 0 | 0 | 0 | 0 | 0 | 0 | 0 |
| *Roseburia_inulinivorans* | 0 | 0 | 0 | 0.000671±0.002225 | 0 | 0 | 0 | 0 |
| *Roseomonas_pecuniae* | 0 | 0 | 0 | 0.004594±0.015238 | 0 | 0 | 0 | 0 |
| *Rothia_dentocariosa* | 0 | 0 | 0 | 0.000671±0.002225 | 0 | 0 | 0 | 0 |
| *Rothia_nasimurium* | 0 | 0.001001±0.003318 | 0 | 0 | 0 | 0 | 0 | 0 |
| *Rubellimicrobium_mesophilum* | 0 | 0.001001±0.003318 | 0 | 0 | 0 | 0 | 0 | 0 |
| *Rubellimicrobium_roseum* | 0 | 0 | 0 | 0.001531±0.005079 | 0 | 0.003498±0.010495 | 0 | 0 |
| *Rubrobacter_radiotolerans* | 0 | 0 | 0.000922±0.003058 | 0 | 0 | 0 | 0 | 0 |
| *Ruminobacter_amylophilus* | 0.004525±0.006947 | 0.008274±0.017248 | 0.007251±0.013158 | 0.002553±0.008467 | 0.008096±0.021115 | 0.002743±0.008230 | 0 | 0.007859±0.015718 |
| *Ruminococcus_albus* | 0 | 0 | 0 | 0 | 0.003102±0.009306 | 0 | 0 | 0.003409±0.006818 |
| *Ruminococcus_bromii* | 0 | 0.007484±0.014961 | 0.020173±0.020214 | 0.011645±0.015817 | 0.002158±0.006474 | 0.008330±0.012663 | 0 | 0.020191±0.022553 |
| *Ruminococcus_callidus* | 0 | 0 | 0.000631±0.002094 | 0 | 0 | 0 | 0 | 0 |
| *Ruminococcus_faecis* | 0 | 0.000847±0.002810 | 0 | 0 | 0 | 0 | 0 | 0 |
| *Ruminococcus_flavefaciens* | 0 | 0 | 0.001326±0.004398 | 0.002802±0.009292 | 0.015510±0.046529 | 0 | 0 | 0.003409±0.006818 |
| *Ruminococcus_gnavus* | 0 | 0 | 0 | 0.001876±0.006221 | 0.003102±0.009306 | 0 | 0 | 0 |
| *Rummeliibacillus_pycnus* | 0 | 0 | 0 | 0.001112±0.003687 | 0 | 0 | 0 | 0 |
| *Salinicoccus_kunmingensis* | 0 | 0 | 0 | 0 | 0 | 0 | 0 | 0.004077±0.008154 |
| *Salmonella_enterica* | 0 | 0.003002±0.009955 | 0.001124±0.003727 | 0.010735±0.035603 | 0 | 0.003239±0.009718 | 0 | 0.003409±0.006818 |
| *Sandaracinobacter_sibiricus* | 0 | 0 | 0.002186±0.007250 | 0 | 0 | 0 | 0 | 0 |
| *Schlegelella_aquatica* | 0 | 0 | 0.000922±0.003058 | 0 | 0 | 0 | 0 | 0 |
| *Sediminibacterium_salmoneum* | 0 | 0.001193±0.003957 | 0 | 0 | 0 | 0 | 0 | 0 |
| *Selenomonas_bovis* | 0 | 0 | 0 | 0 | 0 | 0.001589±0.004766 | 0 | 0 |
| *Serpens_flexibilis* | 0 | 0.002285±0.006280 | 0 | 0 | 0 | 0 | 0 | 0.003015±0.006031 |
| *Serratia_marcescens* | 0 | 0.000847±0.002810 | 0 | 0 | 0.001003±0.003010 | 0.007423±0.011948 | 0 | 0.003015±0.006031 |
| *Serratia_proteamaculans* | 0 | 0.000847±0.002810 | 0 | 0 | 0 | 0 | 0 | 0 |
| *Serratia_quinivorans* | 0.005105±0.015316 | 0 | 0.005402±0.010287 | 0.003694±0.010051 | 0 | 0 | 0.001356±0.003032 | 0 |
| *Sharpea_azabuensis* | 0 | 0 | 0.002652±0.008797 | 0 | 0 | 0 | 0 | 0 |
| *Shewanella_putrefaciens* | 0 | 0 | 0.000631±0.002094 | 0 | 0 | 0.002336±0.007009 | 0 | 0 |
| *Shewanella_xiamenensis* | 0 | 0.002770±0.009187 | 0 | 0 | 0 | 0 | 0 | 0 |
| *Shigella_boydii* | 0 | 0 | 0.000631±0.002094 | 0.001876±0.006221 | 0.009706±0.022761 | 0 | 0 | 0 |
| *Shigella_dysenteriae* | 0 | 0 | 0.000757±0.002509 | 0 | 0.001510±0.004529 | 0 | 0 | 0 |
| *Shinella_granuli* | 0.007002±0.017523 | 0.022167±0.025056 | 0 | 0 | 0 | 0.002743±0.008230 | 0 | 0 |
| *Shinella_kummerowiae* | 0.002158±0.006475 | 0.005812±0.009755 | 0 | 0 | 0 | 0 | 0 | 0 |
| *Simplicispira_psychrophila* | 0 | 0 | 0.001093±0.003625 | 0 | 0 | 0.003498±0.010495 | 0 | 0.007486±0.008712 |
| *Sneathia_sanguinegens* | 0 | 0 | 0 | 0 | 0.003546±0.010639 | 0 | 0 | 0.003015±0.006031 |
| *Solibacillus_silvestris* | 0.222931±0.103967 | 0.330515±0.165167 | 0.348011±0.088842 | 0.250604±0.095488 | 0.278572±0.138560 | 0.248062±0.085114 | 0.302640±0.181089 | 0.304863±0.145173 |
| *Solobacterium_moorei* | 0 | 0 | 0.002459±0.008156 | 0 | 0 | 0 | 0 | 0 |
| *Sorangium_cellulosum* | 0 | 0 | 0.011985±0.039749 | 0 | 0 | 0 | 0 | 0 |
| *Sorghum_bicolor* | 0 | 0 | 0 | 0 | 0.003546±0.010639 | 0 | 0 | 0 |
| *Sphingobacterium_alimentarium* | 0 | 0 | 0 | 0.001876±0.006221 | 0 | 0 | 0 | 0 |
| *Sphingobacterium_daejeonense* | 0 | 0 | 0 | 0.000671±0.002225 | 0 | 0 | 0 | 0 |
| *Sphingobacterium_faecium* | 0 | 0 | 0.002459±0.008156 | 0 | 0.004612±0.009827 | 0 | 0 | 0.003409±0.006818 |
| *Sphingobacterium_multivorum* | 0 | 0 | 0.001093±0.003625 | 0.003751±0.012441 | 0 | 0 | 0 | 0 |
| *Sphingobium_estrogenivorans* | 0.001695±0.005084 | 0 | 0 | 0 | 0 | 0 | 0 | 0 |
| *Sphingobium_yanoikuyae* | 0.002594±0.007781 | 0 | 0 | 0 | 0 | 0 | 0 | 0 |
| *Sphingomonas_asaccharolytica* | 0 | 0 | 0 | 0 | 0 | 0 | 0.001356±0.003032 | 0 |
| *Sphingomonas_astaxanthinifaciens* | 0 | 0 | 0.000757±0.002509 | 0 | 0 | 0 | 0 | 0 |
| *Sphingomonas_dokdonensis* | 0 | 0 | 0 | 0.002013±0.006676 | 0.003546±0.010639 | 0.002336±0.007009 | 0 | 0 |
| *Sphingomonas_faeni* | 0 | 0 | 0.002459±0.008156 | 0 | 0 | 0 | 0.001356±0.003032 | 0 |
| *Sphingomonas_melonis* | 0 | 0.002522±0.008364 | 0 | 0 | 0 | 0 | 0 | 0 |
| *Sphingomonas_panni* | 0.002158±0.006475 | 0.001193±0.003957 | 0.002459±0.008156 | 0.000671±0.002225 | 0 | 0 | 0 | 0 |
| *Sphingomonas_pseudosanguinis* | 0.001958±0.005874 | 0 | 0.001326±0.004398 | 0 | 0 | 0 | 0.002385±0.005332 | 0 |
| *Sphingomonas_yanoikuyae* | 0 | 0.000393±0.001304 | 0.001124±0.003727 | 0.002202±0.005338 | 0.001510±0.004529 | 0.002336±0.007009 | 0 | 0 |
| *Sphingopyxis_alaskensis* | 0 | 0.000393±0.001304 | 0.004918±0.016311 | 0.002553±0.008467 | 0 | 0 | 0 | 0 |
| *Sphingopyxis_chilensis* | 0.004781±0.007421 | 0.006354±0.011518 | 0 | 0 | 0 | 0 | 0 | 0 |
| *Sphingopyxis_panaciterrae* | 0 | 0.000393±0.001304 | 0 | 0 | 0 | 0 | 0 | 0 |
| *Sphingorhabdus_planktonica* | 0 | 0.001339±0.004441 | 0 | 0 | 0 | 0 | 0 | 0 |
| *Spinacia_oleracea* | 0 | 0 | 0.000631±0.002094 | 0.001112±0.003687 | 0 | 0.001589±0.004766 | 0 | 0 |
| *Sporobacter_termitidis* | 0 | 0 | 0.002459±0.008156 | 0.000671±0.002225 | 0 | 0 | 0 | 0 |
| *Sporosarcina_aquimarina* | 0 | 0 | 0 | 0.000671±0.002225 | 0 | 0 | 0 | 0 |
| *Sporosarcina_ginsengi* | 0.002831±0.005763 | 0.005044±0.016727 | 0.008951±0.014710 | 0.002765±0.009170 | 0.001510±0.004529 | 0 | 0.002385±0.005332 | 0.005635±0.006539 |
| *Sporosarcina_koreensis* | 0 | 0.003056±0.007277 | 0 | 0.001531±0.005079 | 0.001003±0.003010 | 0 | 0.002861±0.006398 | 0.004077±0.008154 |
| *Sporosarcina_luteola* | 0 | 0.000393±0.001304 | 0 | 0 | 0 | 0.005875±0.011967 | 0 | 0 |
| *Sporosarcina_newyorkensis* | 0 | 0.002848±0.006944 | 0 | 0.001607±0.005329 | 0 | 0 | 0 | 0 |
| *Sporosarcina_soli* | 0.004288±0.008746 | 0 | 0 | 0 | 0.003102±0.009306 | 0 | 0 | 0.006818±0.013635 |
| *Staphylococcus_aureus* | 0.026260±0.047866 | 0.729896±2.209942 | 0.272944±0.834596 | 0.905007±2.018294 | 0.103821±0.228363 | 1.692672±2.565786 | 0.010846±0.024252 | 0.880473±1.760946 |
| *Staphylococcus_auricularis* | 0 | 0 | 0 | 0.002802±0.009292 | 0 | 0 | 0 | 0.002620±0.005239 |
| *Staphylococcus_capitis* | 0.002158±0.006475 | 0.001339±0.004441 | 0.004832±0.010933 | 0.017692±0.040159 | 0.006494±0.012902 | 0.012462±0.029287 | 0.024973±0.034211 | 0 |
| *Staphylococcus_chromogenes* | 0 | 0 | 0.008526±0.022552 | 0.004820±0.015987 | 0.676589±1.997550 | 0.485859±1.061862 | 1.016293±1.462381 | 0.097847±0.195695 |
| *Staphylococcus_cohnii* | 0 | 0.002522±0.008364 | 0 | 0 | 0 | 0.002336±0.007009 | 0 | 0 |
| *Staphylococcus_devriesei* | 0 | 0 | 0 | 0 | 0 | 0.003239±0.009718 | 0 | 0.003409±0.006818 |
| *Staphylococcus_epidermidis* | 0.011535±0.019197 | 0.005403±0.012433 | 0.005673±0.010247 | 0.005426±0.009830 | 0.009596±0.020064 | 0.021025±0.057907 | 0.020513±0.042181 | 0.006029±0.007079 |
| *Staphylococcus_haemolyticus* | 0 | 0 | 0 | 0 | 0 | 0.003239±0.009718 | 0.003740±0.005435 | 0.010226±0.020453 |
| *Staphylococcus_hominis* | 0 | 0.003617±0.009334 | 0.002083±0.004841 | 0.018629±0.055431 | 0.010699±0.013763 | 0.017881±0.029086 | 0 | 0.002620±0.005239 |
| *Staphylococcus_saprophyticus* | 0.001695±0.005084 | 0.000787±0.002609 | 0 | 0.001112±0.003687 | 0.004612±0.009827 | 0.003378±0.010135 | 0 | 0 |
| *Staphylococcus_schleiferi* | 0 | 0 | 0 | 0.001531±0.005079 | 0 | 0 | 0.005447±0.012179 | 0 |
| *Staphylococcus_sciuri* | 0 | 0 | 0 | 0 | 0 | 0 | 0.007154±0.015997 | 0 |
| *Staphylococcus_simiae* | 0 | 0.002753±0.009130 | 0 | 0 | 0 | 0 | 0 | 0 |
| *Staphylococcus_succinus* | 0 | 0 | 0.001326±0.004398 | 0 | 0.001003±0.003010 | 0 | 0 | 0 |
| *Staphylococcus_warneri* | 0.001958±0.005874 | 0.007899±0.017223 | 0 | 0 | 0 | 0 | 0 | 0 |
| *Stenotrophomonas_geniculata* | 0.014513±0.013337 | 0.008740±0.014575 | 0.020624±0.030935 | 0.025523±0.052214 | 0.003516±0.007067 | 0.003239±0.009718 | 0.027708±0.028426 | 0.070135±0.051047 |
| *Stenotrophomonas_humi* | 0 | 0 | 0.001124±0.003727 | 0 | 0 | 0 | 0 | 0 |
| *Stenotrophomonas_koreensis* | 0 | 0.000787±0.002609 | 0.002459±0.008156 | 0.002553±0.008467 | 0.011044±0.018912 | 0.009977±0.020903 | 0.001356±0.003032 | 0.018086±0.021238 |
| *Stenotrophomonas_maltophilia* | 0.009029±0.015595 | 0.010076±0.018479 | 0.061731±0.120842 | 0.017340±0.025927 | 0.024018±0.045932 | 0.012400±0.020747 | 0.010453±0.014889 | 0.022681±0.027241 |
| *Stenotrophomonas_nitritireducens* | 0 | 0 | 0 | 0.000671±0.002225 | 0 | 0 | 0 | 0 |
| *Stenotrophomonas_retroflexus* | 0 | 0 | 0.001901±0.006305 | 0 | 0 | 0 | 0 | 0 |
| *Stenotrophomonas_rhizophila* | 0.014051±0.018708 | 0.009069±0.015352 | 0.005593±0.009723 | 0.009642±0.013877 | 0.009843±0.018078 | 0.011048±0.017195 | 0.007831±0.012012 | 0 |
| *Stenotrophomonas_terrae* | 0.001129±0.003386 | 0.000669±0.002220 | 0.002419±0.005413 | 0.000671±0.002225 | 0.002251±0.006754 | 0.001589±0.004766 | 0.002385±0.005332 | 0.003015±0.006031 |
| *Streptococcus_agalactiae* | 0.084245±0.085826 | 0.192585±0.394875 | 0.076713±0.055202 | 2.142298±7.057177 | 0.660744±1.846369 | 0.300214±0.773612 | 0.108209±0.157543 | 0.298944±0.546113 |
| *Streptococcus_australis* | 0 | 0 | 0 | 0 | 0 | 0 | 0.001356±0.003032 | 0 |
| *Streptococcus_cristatus* | 0 | 0 | 0 | 0 | 0 | 0 | 0 | 0.002620±0.005239 |
| *Streptococcus_dysgalactiae* | 0.002158±0.006475 | 0.000669±0.002220 | 0.001093±0.003625 | 0.005112±0.010370 | 0.005353±0.010793 | 0.002743±0.008230 | 0 | 0 |
| *Streptococcus_gallolyticus* | 0 | 0.018039±0.039981 | 0 | 0 | 0.003102±0.009306 | 0 | 0 | 0.010226±0.020453 |
| *Streptococcus_infantarius* | 0 | 0 | 0.002459±0.008156 | 0 | 0 | 0 | 0.001356±0.003032 | 0 |
| *Streptococcus_infantis* | 0.001958±0.005874 | 0 | 0.003082±0.005579 | 0 | 0.006204±0.018612 | 0 | 0.001356±0.003032 | 0 |
| *Streptococcus_mitis* | 0.004552±0.009144 | 0.001670±0.003804 | 0.013847±0.026653 | 0.015388±0.028585 | 0.083035±0.199668 | 0.010616±0.013231 | 0 | 0.006029±0.007079 |
| *Streptococcus_parasanguinis* | 0 | 0 | 0 | 0 | 0 | 0.003239±0.009718 | 0 | 0 |
| *Streptococcus_parauberis* | 0.016735±0.018262 | 0.020164±0.026542 | 0.033987±0.018268 | 0.022737±0.027641 | 0.028149±0.027389 | 0.029337±0.024756 | 0.003740±0.005435 | 0.028316±0.027110 |
| *Streptococcus_salivarius* | 0.026277±0.027262 | 0.059011±0.056590 | 0.019840±0.026600 | 0.024406±0.027711 | 0.039169±0.042729 | 0.025420±0.037077 | 0.049862±0.080600 | 0.019664±0.032716 |
| *Streptococcus_sanguinis* | 0 | 0 | 0.000631±0.002094 | 0.003023±0.010027 | 0 | 0 | 0.002385±0.005332 | 0 |
| *Streptococcus_thermophilus* | 0.002594±0.007781 | 0 | 0 | 0 | 0 | 0 | 0 | 0 |
| *Streptococcus_uberis* | 0.003916±0.011747 | 0.003211±0.008499 | 0.000631±0.002094 | 0.001863±0.006180 | 0 | 0 | 0 | 0.003409±0.006818 |
| *Succinivibrio_dextrinosolvens* | 0 | 0.007210±0.019609 | 0.006306±0.016145 | 0.002546±0.006394 | 0.002158±0.006474 | 0.002336±0.007009 | 0.008158±0.012172 | 0.006697±0.008091 |
| *Sulfurospirillum_cavolei* | 0 | 0 | 0 | 0.001876±0.006221 | 0.002007±0.006020 | 0.003239±0.009718 | 0.001356±0.003032 | 0.005635±0.006539 |
| *Sunxiuqinia_faeciviva* | 0.001885±0.005655 | 0 | 0 | 0 | 0 | 0 | 0 | 0 |
| *Sutterella_stercoricanis* | 0 | 0 | 0 | 0 | 0.006204±0.018612 | 0 | 0 | 0 |
| *Tardiphaga_robiniae* | 0 | 0.000393±0.001304 | 0 | 0 | 0 | 0 | 0 | 0 |
| *Terribacillus_saccharophilus* | 0 | 0 | 0 | 0 | 0 | 0 | 0 | 0.005239±0.010479 |
| *Tessaracoccus_flavescens* | 0 | 0 | 0 | 0.001876±0.006221 | 0 | 0 | 0 | 0 |
| *Tessaracoccus_lubricantis* | 0 | 0 | 0 | 0 | 0.002158±0.006474 | 0 | 0 | 0 |
| *Tetragenococcus_halophilus* | 0 | 0.000847±0.002810 | 0 | 0 | 0 | 0 | 0 | 0 |
| *Thermoactinomyces_vulgaris* | 0 | 0 | 0 | 0.002910±0.009651 | 0 | 0 | 0 | 0 |
| *Thermomonas_haemolytica* | 0 | 0.001339±0.004441 | 0 | 0 | 0 | 0 | 0 | 0 |
| *Thermus_scotoductus* | 0 | 0.000669±0.002220 | 0 | 0 | 0 | 0 | 0 | 0 |
| *Tissierella_creatinophila* | 0 | 0 | 0 | 0 | 0 | 0.003044±0.009132 | 0 | 0 |
| *Tolumonas_osonensis* | 0 | 0 | 0 | 0.003023±0.010027 | 0 | 0 | 0 | 0 |
| *Trabulsiella_farmeri* | 0 | 0 | 0 | 0.001112±0.003687 | 0 | 0 | 0 | 0 |
| *Trichococcus_palustris* | 0 | 0.000847±0.002810 | 0 | 0 | 0 | 0.003250±0.009749 | 0 | 0 |
| *Trichococcus_pasteurii* | 0 | 0.000393±0.001304 | 0.000757±0.002509 | 0.004428±0.009993 | 0.021044±0.042425 | 0.003044±0.009132 | 0 | 0 |
| *Truepera_radiovictrix* | 0 | 0 | 0 | 0.000671±0.002225 | 0 | 0 | 0 | 0 |
| *Tumebacillus_ginsengisoli* | 0 | 0.002522±0.008364 | 0 | 0 | 0 | 0 | 0 | 0 |
| *Turicibacter_sanguinis* | 0.017424±0.031776 | 0.015016±0.027578 | 0.083272±0.181026 | 0.044207±0.045800 | 0.036531±0.047592 | 0.020994±0.021904 | 0.041010±0.044974 | 0.050782±0.037098 |
| *Uruburuella_suis* | 0.002158±0.006475 | 0.001001±0.003318 | 0 | 0.002553±0.008467 | 0.002635±0.005300 | 0.013061±0.021300 | 0 | 0 |
| *Vagococcus_salmoninarum* | 0.002594±0.007781 | 0 | 0.004663±0.007138 | 0.000671±0.002225 | 0.003392±0.010175 | 0 | 0.002385±0.005332 | 0 |
| *Vampirovibrio_chlorellavorus* | 0.001695±0.005084 | 0.013253±0.024176 | 0.005088±0.009729 | 0.002553±0.008467 | 0.015510±0.046529 | 0.005576±0.011249 | 0.022897±0.041053 | 0.007859±0.015718 |
| *Variovorax_boronicumulans* | 0 | 0 | 0.000631±0.002094 | 0 | 0 | 0 | 0 | 0 |
| *Variovorax_ginsengisoli* | 0.001885±0.005655 | 0 | 0.000631±0.002094 | 0 | 0 | 0 | 0 | 0 |
| *Variovorax_paradoxus* | 0.004532±0.010348 | 0.020157±0.032555 | 0.003915±0.009357 | 0.004422±0.012418 | 0.003761±0.007647 | 0.005576±0.011249 | 0.001356±0.003032 | 0 |
| *Veillonella_dispar* | 0 | 0.000847±0.002810 | 0.000757±0.002509 | 0 | 0 | 0 | 0 | 0 |
| *Veillonella_parvula* | 0 | 0.000669±0.002220 | 0 | 0 | 0 | 0 | 0 | 0 |
| *Vibrio_cincinnatiensis* | 0 | 0.000393±0.001304 | 0.000631±0.002094 | 0 | 0.006204±0.018612 | 0.003539±0.010616 | 0.006802±0.011792 | 0 |
| *Victivallis_vadensis* | 0 | 0.002561±0.006443 | 0.001326±0.004398 | 0 | 0 | 0 | 0.004769±0.010664 | 0 |
| *Virgibacillus_halodenitrificans* | 0 | 0 | 0.001783±0.005914 | 0 | 0 | 0 | 0 | 0 |
| *Wautersiella_falsenii* | 0 | 0 | 0.000631±0.002094 | 0 | 0 | 0 | 0 | 0 |
| *Weissella_confusa* | 0 | 0.001001±0.003318 | 0.000631±0.002094 | 0 | 0 | 0 | 0 | 0.003015±0.006031 |
| *Xenophilus_aerolatus* | 0 | 0.002770±0.009187 | 0 | 0 | 0 | 0 | 0 | 0 |
| *Xylophilus_ampelinus* | 0 | 0 | 0 | 0.001531±0.005079 | 0 | 0 | 0 | 0 |
| *Yeosuana_aromativorans* | 0 | 0 | 0 | 0 | 0 | 0 | 0.001356±0.003032 | 0 |
| *Yersinia_aleksiciae* | 0 | 0.000393±0.001304 | 0.000631±0.002094 | 0 | 0.004529±0.013587 | 0.002336±0.007009 | 0.001356±0.003032 | 0 |
| *Yersinia_enterocolitica* | 0 | 0 | 0 | 0 | 0.006204±0.018612 | 0 | 0 | 0 |
| *Zhihengliuella_aestuarii* | 0 | 0 | 0.000631±0.002094 | 0 | 0 | 0 | 0 | 0 |
| *gut_metagenome* | 0 | 0 | 0.001093±0.003625 | 0 | 0 | 0 | 0 | 0 |
| *unclassified* | 18.816550±5.639278 | 17.997623±7.351247 | 19.444032±5.932187 | 21.992940±5.384319 | 19.535343±4.302635 | 18.585216±4.740165 | 16.833369±6.548091 | 18.882422±7.724053 |
| *uncultured_eubacterium_WCHB1-25* | 0 | 0.001892±0.006275 | 0.000922±0.003058 | 0 | 0.003392±0.010175 | 0.003239±0.009718 | 0 | 0 |
