# Supplementary figures and images for: A Novel Lactobacilli-Based Teat Disinfectant for Improving Bacterial Communities in the Milks of Cow Teats with Subclinical Mastitis
Source: Front Microbiol. 2017 Sep 26;8:1782. doi: 10.3389/fmicb.2017.01782 (PMC5622921; doi:10.3389/fmicb.2017.01782)

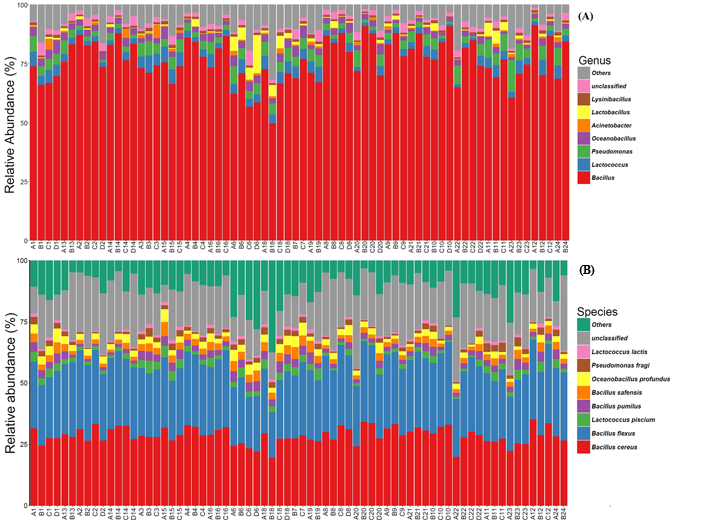

Supplement: Supplementary file 4 [file Image_1.tif]
